# Selective inhibition of IL-6 trans-signalling reveals distinct mechanisms of human classic and trans-signalling receptor-complex formation

**DOI:** 10.1038/s42003-026-11014-0

**Published:** 2026-10-14

**Authors:** Emelie Rieneck, Jan René Haak, Kamal Asgarov, Jean-René Pallandre, Nicole Wundrack, Julia Ettich, Niloufarsadat Miri, Doreen M. Floss, Jürgen Scheller, Christoph Garbers, Andy Clark, John Wijdenes, Fred Schaper, Anna Dittrich

**Affiliations:** 1 Otto von Guericke University Magdeburg, Institute of Biology, Chair of Systems Biology, Magdeburg, Germany; 2 INSERM U1098, Université de Franche-Comté, Besançon, France; 3 Institute for Biochemistry and Molecular Biology II, Medical Faculty and University Hospital Düsseldorf, Heinrich Heine University Düsseldorf, Düsseldorf, Germany; 4 Institute of Clinical Biochemistry, Hannover Medical School, Hannover, Germany; 5 Mabdesigns Ltd, London, UK; 6 Center for Dynamic Systems: Systems Engineering (CDS), Magdeburg, Germany; 7 Magdeburg Center for Systems Biology (MACS), Magdeburg, Germany; 8 Center for Health and Medical Prevention (CHaMP), Magdeburg, Germany; 9 Present Address: Qpliance GmbH, Brieselang, Germany; 10 Present Address: RNACoreX, Avanne-Aveney, France; 11 Present Address: Etablissement Français du Sang - Bourgogne Franche-Comté (EFS BFC), Besançon, France; 12 Present Address: Otto von Guericke University Magdeburg, Faculty of Medicine, Department of Microgravity and Translational Regenerative Medicine, Magdeburg, Germany

**Keywords:** Interleukins, Membrane proteins, Cell signalling

## Abstract

The cytokine interleukin-6 orchestrates inflammation through two distinct pathways of receptor activation: anti-inflammatory classic signalling via membrane-bound interleukin-6 receptor and pro-inflammatory trans-signalling via soluble interleukin-6 receptor. Current interleukin-6-targeted therapies block both pathways, thereby disrupting essential homeostatic functions initiated by classic signalling. We here show a high-affinity, human-specific antibody that selectively inhibits interleukin-6 trans-signalling. This antibody maintains classic signalling-induced expression of hepatic acute phase genes while completely blocking trans-signalling-induced activation of endothelial cells. By targeting the domain D2 of membrane-bound and soluble human interleukin-6 receptor, the antibody selectively prevents formation of the trans-signalling receptor complex without interfering with ligand binding. This selective blockade demonstrates that human interleukin-6 classic and trans-signalling proceed through distinct receptor assemblies. Our findings describe mechanistic insight into interleukin-6 receptor assembly and provide a strategy to suppress inflammatory interleukin-6 activity while preserving regenerative and anti-inflammatory signalling, thus offering both conceptual and potential therapeutic advances in the targeted modulation of cytokine signalling networks.

## Abstract (web summary)

Rieneck et al. use a human specific interleukin-6 receptor antibody to investigate the molecular basis of human interleukin-6 receptor complex formation in classic and trans-signalling. We find that the two pathways require distinct receptor assemblies, enabling selective inhibition of trans-signalling without affecting classic signalling.

## Introduction

The cytokine interleukin 6 (IL-6) is involved in the regulation of pro- and anti-inflammatory responses, but also has metabolic and regenerative functions^1,2^. Whether IL-6 triggers pro-inflammatory or anti-inflammatory and regenerative responses, depends on the mode in which IL-6 signalling is activated. Classic signalling is initiated by binding of IL-6 to membrane-bound IL-6 receptor α (IL-6R)^3^, whilst trans-signalling is initiated by binding of IL-6 to a soluble form of the IL-6R (sIL-6R), generated by shedding or alternative splicing^4,5^. IL-6 classic signalling is mostly associated with the anti-inflammatory and regenerative functions of IL-6. Classic signalling is one of the strongest activators of the hepatic acute-phase response (APR) and plays a major role during liver regeneration^6,7^. Trans-signalling is primarily associated with the pro-inflammatory functions of  IL-6. IL-6 trans-signalling is central in the aetiology of, e.g., intestinal inflammation^8^, colon cancer^9^, sepsis^10,11^, nephrotoxic nephritis^12^, perioperative neurocognitive disorder^13^, depression^14^, long COVID, and endotheliopathy^15^.

**Fig. 1:**
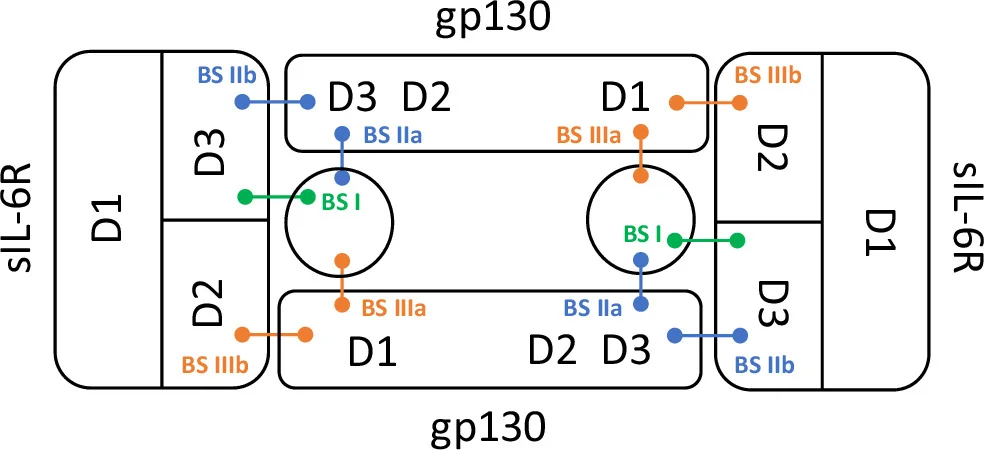
Five binding sites facilitate formation of an active IL-6 receptor-complex. Schematic representation of the interacting domains and binding sites within the hexameric active IL-6 receptor-complex (BS-I (green), BS-IIa and b (blue), BS-IIIa and b (orange)).

Binding of IL-6:IL-6R or IL-6:sIL-6R complexes to gp130 results in formation of a hexameric active IL-6 receptor-complex^16^. The extracellular part of IL-6R consists of three domains (D1–D3)^17^. Domain D1 is not involved in ligand recognition^17^. D2 and D3 form the cytokine binding homology region (CHR)^16^. The five binding sites (BS) I, IIa, IIb, IIIa, and IIIb connect IL-6, IL-6R and gp130 (Fig. 1)^16,18^. The IL-6:(s)IL-6R dimer is formed through BS-I which connects IL-6 with the CHR in IL-6R. Within the CHR D3 provides most of the contact surface with IL-6^18^. The IL-6:(s)IL-6R dimer binds to D1 of the first gp130. D1 of gp130 is connected to IL-6 via BS-IIIa and to D2 of the IL-6R via BS-IIIb. D2 and D3 of the second gp130 interact with IL-6 via BS-IIa and the D3 domains of gp130 and IL-6R interact with each other via BS-IIb^18^.

Formation of an active IL-6 receptor-complex results in activation of gp130-associated Janus kinases (Jaks). Jaks phosphorylate tyrosine motifs in the intracellular region of gp130. These phosphorylated motifs recruit proteins with Src-homology 2 (SH2) domains such as signal transducer and activator of transcription 3 (STAT3). STAT3 is phosphorylated by Jaks, dimerizes and translocates into the nucleus where it acts as a transcription factor^19,20^.

Only a small number of cells, such as hepatocytes and some leukocyte subsets express membrane-bound IL-6R^21,22^. In contrast, gp130 is almost ubiquitously expressed^23,24^ and is also utilized by other members of the IL-6 cytokine super-family including IL-11^25^. Thus, classic signalling is limited to specific cell types, while trans-signalling can be initiated on nearly all cells. In cells expressing lower numbers of IL-6R than gp130, trans-signalling is stronger than classic signalling^26^.

Under physiological conditions IL-6 levels in human serum are low (<5 pg ml^−1^), but rapidly increase to ng or even µg per ml upon immune activation^27^. The sIL-6R is constitutively present in human serum at high concentrations (20–150 ng ml^−1^), with further elevation during inflammation^28^. Consequently, in vivo classic signalling always occurs in the presence of sIL-6R. Soluble gp130 (sgp130) antagonizes trans-signalling and fulfils a buffer function that restricts systemic signalling^29^.

All clinically used antibodies to treat IL-6-associated diseases simultaneously block classic and trans-signalling^30^. The most commonly prescribed drug, Tocilizumab (Toc) (Actemra, Hoffmann-La Roche), a humanized anti-IL-6R monoclonal antibody (mAb), is approved for treatment of rheumatoid arthritis, systemic juvenile idiopathic arthritis and cytokine storms induced by chimeric antigen receptor (CAR) T cell therapy and COVID-19^1,31^. Tocilizumab interferes with binding of IL-6 to IL-6R and sIL-6R by blocking BS-I^32^. Serious side effects of Tocilizumab, such as an increased risk for bacterial infections, gut perforation, neutropenia, increased levels of cholesterol and weight gain are, at least in part, attributed to inhibition of classic signalling^33–36^. The selective inhibition of trans-signalling as a therapeutic approach against IL-6-related diseases poses a major challenge, but offers the prospect of effective therapy with reduced side effects. Of note, also protective effects of trans-signalling on the function of intestinal epithelial cells have been reported^37^. This highlights the need to understand differences between classic and trans-signalling in a disease and cell type-specific context.

Thus far, the only selective inhibitor of human trans-signalling is sgp130Fc (Olamkicept (Olam)), that completed two phase 2 clinical trials for treatment of IBD and ulcerative colitis^38,39^. Olamkicept is based on the endogenous sgp130^29^. It inhibits classic signalling to some extent, probably through trapping IL-6 in IL-6:sIL-6R:sgp130Fc complexes^40^. Additionally, Olamkicept interferes with other members of the IL-6 cytokine super-family including IL-11^41^.

The IL-6R mAb 25F10 selectively inhibits murine trans-signalling, presumably by interfering with the formation of an active murine IL-6 receptor-complex^42^. 25F10 binds to BS-IIb within D3 of murine IL-6R^42^. Interestingly, mAb NI-1201, which targets the corresponding epitope in the human IL-6R, was unable to selectively inhibit human trans-signalling^43^. This underscores that the structure of the IL-6 receptor-complex in mice and humans is different^44^.

In summary, we here present an anti-human IL-6R antibody that selectively blocks human IL-6 trans-signalling with high cytokine and species specificity. This offers the potential of therapeutic blockade of inflammatory trans-signalling without inhibiting the protective functions of classic signalling. In addition, we show that human classic and trans-signalling operate via distinct receptor-assemblies.

## Results

### Identifying anti-IL-6R Fabs that preferentially block IL-6 trans-signalling

Selective inhibition of human IL-6 trans-signalling is a major challenge in the development of anti-inflammatory drugs. We initially aimed to generate antibodies that selectively bind sIL-6R. A subtractive immunisation protocol was used to produce such antibodies. This immunisation protocol eliminates B-cell responses against dominant epitopes, thereby redirecting the immune response toward less immunogenic functionally distinct epitopes that would likely be missed using conventional immunisation protocols (Supplementary Note 1). Mice were first immunised with JV1 cells expressing membrane-bound human IL-6R. Subsequently, mice were treated with cyclophosphamide, which depletes reactive B-cells, to obtain tolerance to membrane-bound IL-6R. Following four cycles of immunisation and depletion, mice were immunised with sIL-6R. Subsequently, mice were killed and total RNA was isolated from splenocytes. A fragment antigen-binding region (Fab) library was created using phage display technology. Fab-containing periplasmic extracts were collected from 27 single clones. To identify Fabs that selectively inhibit trans-signalling, we analysed classic and trans-signalling-induced proliferation (Supplementary Table 1A) and STAT3 phosphorylation (Supplementary Table 1B) in the absence and presence of specific Fab-containing periplasmic extracts. Fabs C07, C09, C12, C19, and C20 were identified as potentially selective inhibitors of IL-6 trans-signalling. These five anti-IL-6R Fabs have highly similar but not identical complementarity-determining regions (CDR) suggesting that they bind to overlapping epitopes (Supplementary Fig. 1). For further characterisation these Fabs were chimerised with human IgG1 and purified.

### IL-6 trans-signalling-induced proliferation is selectively inhibited by anti-IL-6R mAbs

Proliferation of cells of the murine pro B-cell line Ba/F3 depends on the presence of cytokines^45^. As Ba/F3 wildtype (Ba/F3_wt) cells express neither gp130 nor IL-6R (Supplementary Fig. 2A), they do not respond to IL-6. Stable expression of human (hu) gp130 alone (Ba/F3_hugp130) (Supplementary Fig. 2B) or together with human IL-6R (Ba/F3_hugp130_huIL-6R) (Supplementary Fig. 2C) enables proliferation in response to human IL-6 classic and trans-signalling^46,47^, which is a proven tool in IL-6-biology research^26,29,40^.

**Fig. 2:**
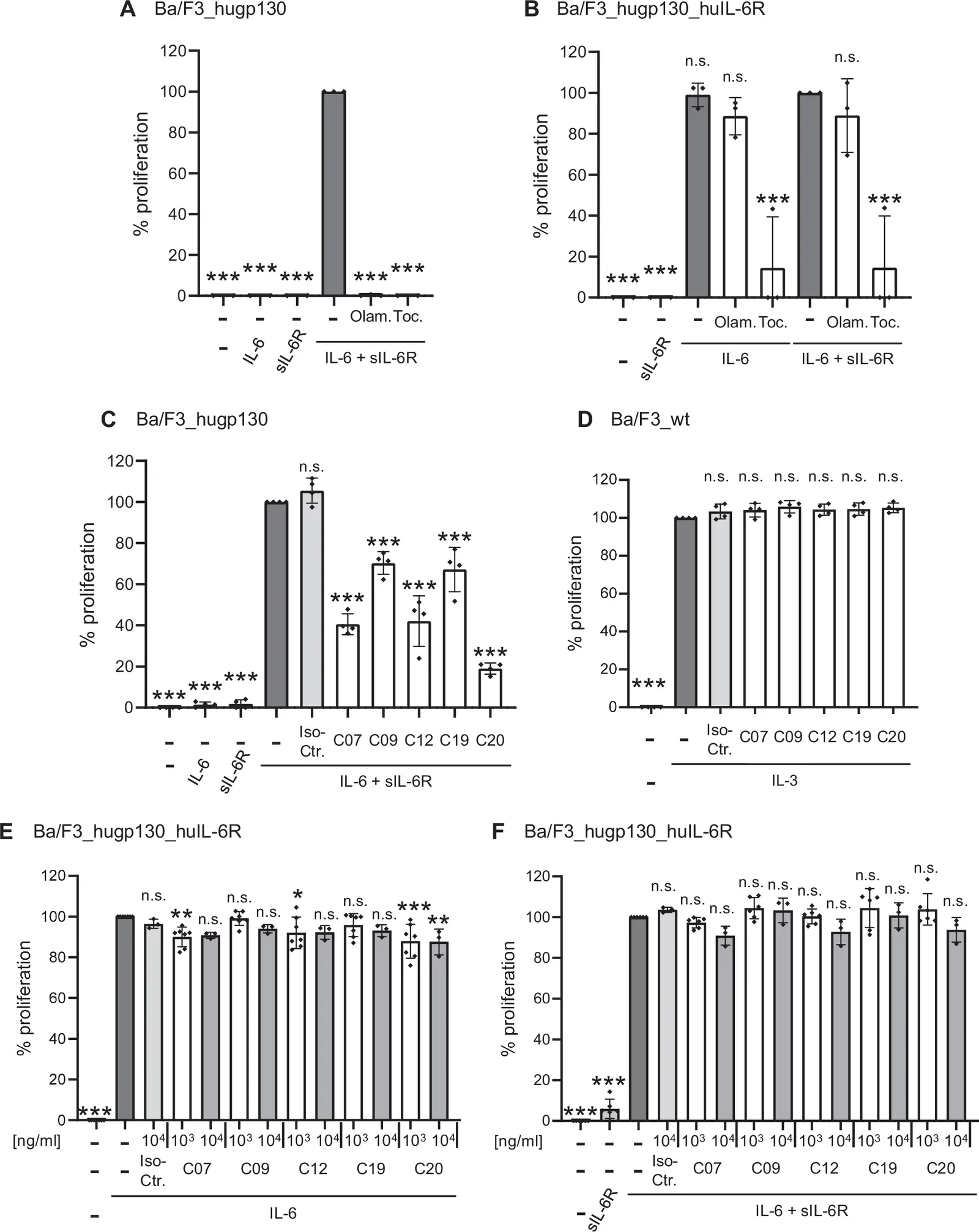
IL-6-trans-signalling-induced proliferation is selectively inhibited by anti-IL-6R mAbs. **A** Ba/F3_hugp130 cells or **B** Ba/F3_hugp130_huIL-6R cells were cultured with huIL-6 (10 ng ml^−1^), husIL-6R (500 ng ml^−1^) alone or with a combination of huIL-6 and husIL-6R. Cells were additionally incubated with Olamkicept (1.6 µg ml^−1^; 6.7 nM) or Tocilizumab (10 µg ml^−1^; 66.7 nM). **C** Ba/F3_hugp130 cells were cultured with huIL-6 (10 ng ml^−1^), husIL-6R (500 ng ml^−1^) alone or with a combination of huIL-6 and husIL-6R. Cells were additionally incubated with an IgG isotype control (1H7) (1 µg ml^−1^; 6.7 nM) or with the indicated anti-IL-6R mAb (1 µg ml^−1^; 6.7 nM). **D** Ba/F3_wt cells were cultured with muIL-3 (10 ng ml^−1^). Cells were additionally incubated with an IgG isotype control (1H7) (1 µg ml^−1^; 6.7 nM) or with the indicated anti-IL-6R mAb (1 µg ml^−1^; 6.7 nM). **E** Ba/F3_hugp130_huIL-6R cells were cultured with huIL-6 (10 ng ml^−1^). Cells were additionally incubated with an IgG isotype control (1H7) (10 µg ml^−1^; 66.7 nM) or with the indicated anti-IL-6R mAb (1 or 10 µg ml^−1^; 6.7 nM or 66.7 nM). **F** Ba/F3_hugp130_huIL-6R cells were cultured with husIL-6R (500 ng ml^−1^) or with a mixture of huIL-6 (10 ng ml^−1^) and husIL-6R. Cells were additionally incubated with an IgG isotype control (1H7) (10 µg ml^−1^; 66.7 nM) or with the indicated anti-IL-6R mAb (1 or 10 µg ml^−1^; 6.7 nM or 66.7 nM). Viable cells were determined using Cell Titer Blue reagent. The absorbance of cell suspensions stimulated with cytokine but without mAbs was set to 100%. Mean and SD of *n* = 3–6 independent experiments are shown. One-way ANOVA with multiple comparisons: n.s. non-significant, *p < 0.05, **p < 0.01, ***p < 0.001. *P*-values indicate differences to cells incubated with cytokine but without mAbs.

First, we confirmed the applicability of this model to analyse selective inhibition of IL-6 trans- and classic signalling. As a model for trans-signalling Ba/F3_hugp130 cells were treated with human IL-6 together with human sIL-6R and either Olamkicept or Tocilizumab. As control, cells were left untreated or incubated with IL-6 or sIL-6R alone. After 48 h the relative number of viable cells was determined. Ba/F3_hugp130 cells do not proliferate in response to IL-6 or sIL-6R alone, while stimulation with IL-6 and sIL-6R results in trans-signalling-induced proliferation (Fig. 2A). As described earlier^40^, trans-signalling is blocked by Olamkicept and Tocilizumab.

To study IL-6 classic signalling Ba/F3_hugp130_huIL-6R cells were stimulated with only IL-6. To mimic physiological conditions, cells were stimulated with IL-6 together with sIL-6R. We confirmed that classic signalling in the absence and the presence of sIL-6R is blocked by Tocilizumab, but not by Olamkicept (Fig. 2B)^40,48^.

Next, the ability of the purified anti-IL-6R mAbs C07, C09, C12, C19, and C20 to block human trans-signalling-induced proliferation was determined. Ba/F3_hugp130 cells were stimulated with IL-6 and sIL-6R. Where indicated, the cells were additionally incubated with one of the purified anti-IL-6R mAbs (1 µg ml^−1^). The IgG isotype control mAb^49^, that was produced using a similar protocol as the anti-IL-6R mAbs, serves as a control for unspecific inhibition of proliferation by human IgGs. All anti-IL-6R mAbs reduce trans-signalling-induced proliferation, with C20 showing the best inhibitory effect (Fig. 2C).

None of the mAbs interfere with IL-3-induced proliferation (Fig. 2D), suggesting that the inhibition of IL-6 trans-signalling-induced proliferation (Fig. 2C) depends on IL-6 signalling.

Next, we investigated whether the anti-IL-6R mAbs block classic signalling. Ba/F3_hugp130_huIL-6R cells were incubated with IL-6 and the indicated mAbs. In contrast to the strong inhibition of IL-6 trans-signalling-induced proliferation at already 1 µg ml^−1^ (Fig. 2C) the anti-IL-6R mAbs only slightly impair classic signalling-induced proliferation even at a 10-fold higher concentration (Fig. 2E).

To determine whether the anti-IL-6R mAbs interfere with classic signalling in a more physiological environment, Ba/F3_hugp130_huIL-6R cells were incubated with IL-6 together with sIL-6R and the indicated anti-IL-6R mAbs. The anti-IL-6R mAbs do not inhibit classic signalling in the presence of sIL-6R (Fig. 2F).

In summary, the anti-IL-6R mAbs reduce trans-signalling-induced proliferation without or with only marginally affecting classic signalling-induced proliferation.

### Proliferation induced by IL-6 trans-signalling is inhibited by anti-IL-6R mAbs in a dose-dependent manner

**Fig. 3:**
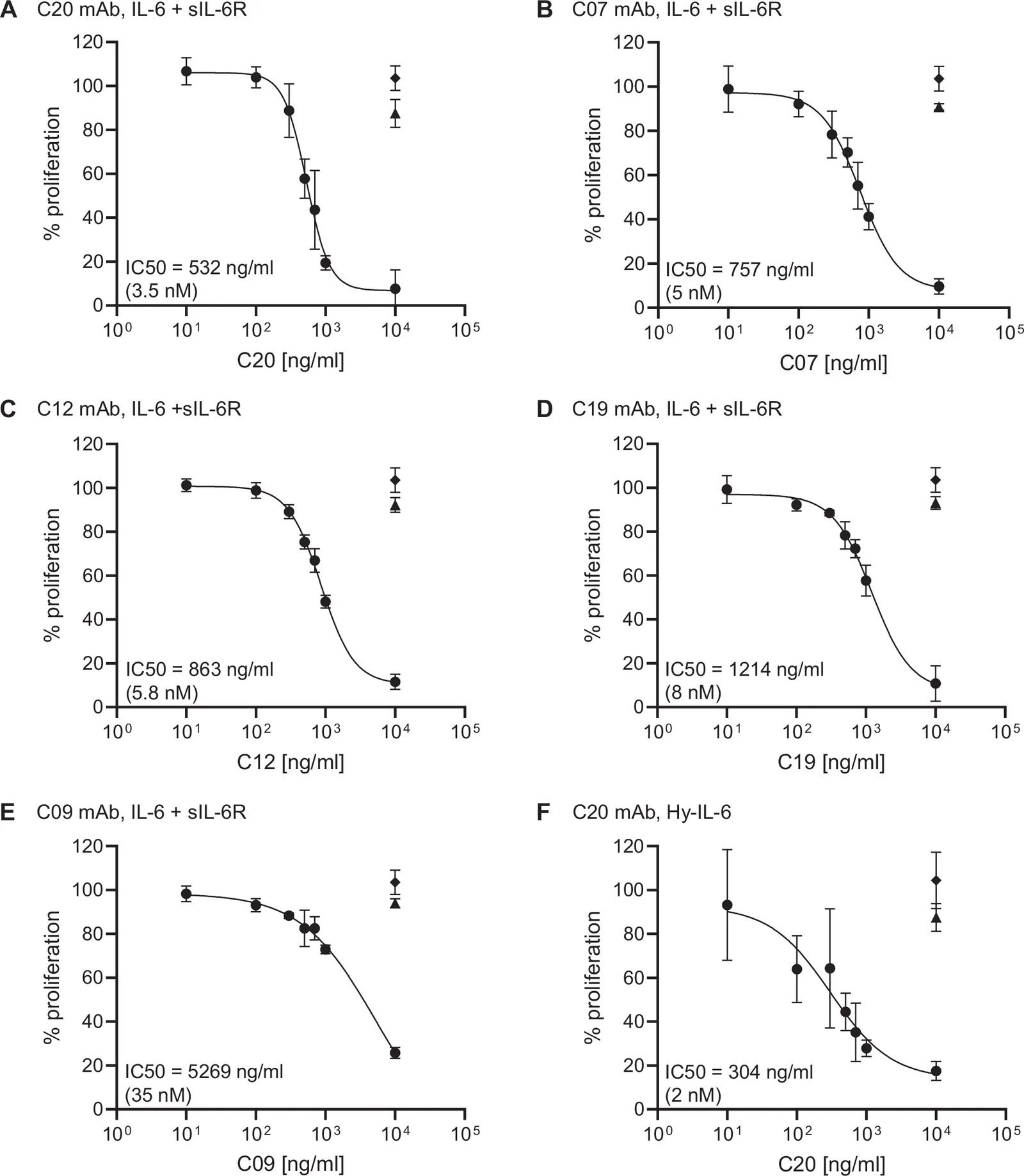
Proliferation induced by IL-6 trans-signalling is inhibited by anti-IL-6R mAbs in a dose-dependent manner. Ba/F3_hugp130 cells were cultured with a mixture of huIL-6 (10 ng ml^−1^) and husIL-6R (200 ng ml^−1^), increasing amounts of the indicated anti-IL-6R mAbs C20 (**A**), C07 (**B**), C12 (**C**), C19 (**D**), C09 (**E**) or with an IgG isotype control (1H7) (diamond, 10 µg ml^−1^; 66.7 nM). The percentage of classic signalling-induced proliferation in the presence of the indicated mAbs in Ba/F3_hugp130_huIL-6R cells is shown in triangles. **F** Ba/F3_hugp130 cells were cultured with Hy-IL-6 (28 ng ml^−1^) and increasing amounts of the anti-IL-6R mAb C20 or with an IgG isotype control (1H7) (diamond, 10 µg ml^−1^; 66.7 nM). The percentage of classic signalling-induced proliferation in the presence of the C20 mAb in Ba/F3_hugp130_huIL-6R cells is shown as a triangle. Viable cells were determined using Cell Titer Blue reagent. The absorbance of cell suspensions grown with huIL-6 and husIL-6R or Hy-IL-6 in the absence of mAbs was set to 100%. Mean and SD of *n* = 3 independent experiments are shown. Curves were fitted with a four-parameter non-linear fit.

The anti-IL-6R mAbs C07, C09, C12, C19, and C20 reduce trans-signalling-induced proliferation to varying degrees (Fig. 2C). To determine which mAbs have the greatest potential for inhibiting IL-6 trans-signalling-induced proliferation, IC50s were determined for each anti-IL-6R mAb. Ba/F3_hugp130 cells were incubated with IL-6, sIL-6R and increasing amounts of the specified mAb. As a negative control, Ba/F3_hugp130 cells stimulated with IL-6 and sIL-6R were treated with the IgG isotype control (Fig. 3A–E, diamonds). Proliferation was determined and IC50 values for the inhibition of IL-6 trans-signalling, were calculated. The anti-IL-6R mAb C20 (Fig. 3A) is the most efficient inhibitor of the antibodies tested, with an IC50 of 3.5 nM. For C07 (Fig. 3B), C12 (Fig. 3C), and C19 (Fig. 3D) IC50 values ≥ 5 nM were calculated, while C09 (Fig. 3E) has a 10-fold higher IC50 value compared to C20. In the same experimental setting Tocilizumab and Olamkicept have an IC50 of 0.27 nM and 0.003 nM, respectively^40^.

To validate the selectivity of the anti-IL-6R mAbs for trans-signalling, the percentage of classic signalling-induced proliferation of Ba/F3_hugp130_huIL-6R cells in the presence of the indicated mAbs is shown (Fig. 3A–F, triangles).

Replacing IL-6 and sIL-6R by hyper IL-6 (Hy-IL-6)^50^, which is a fusion protein composed of sIL-6R and IL-6, resulted in similar IC50 values for all anti-IL-6R mAbs (Fig. 3F, Supplementary Fig. 3). An overview of all determined IC50 values is given in Supplementary Table 2.

### Jak/STAT signalling induced by IL-6 trans-signalling is selectively inhibited by the anti-IL-6R mAbs

Proliferation is a highly disease-associated cellular response to IL-6. However, analysis of early signalling events such as STAT3 phosphorylation is a more direct measure of the effects of the anti-IL-6R mAbs on IL-6 signalling. We therefore, tested whether the anti-IL-6R mAbs disrupt early intracellular IL-6-induced trans- and classic signalling in a human cell model.

**Fig. 4:**
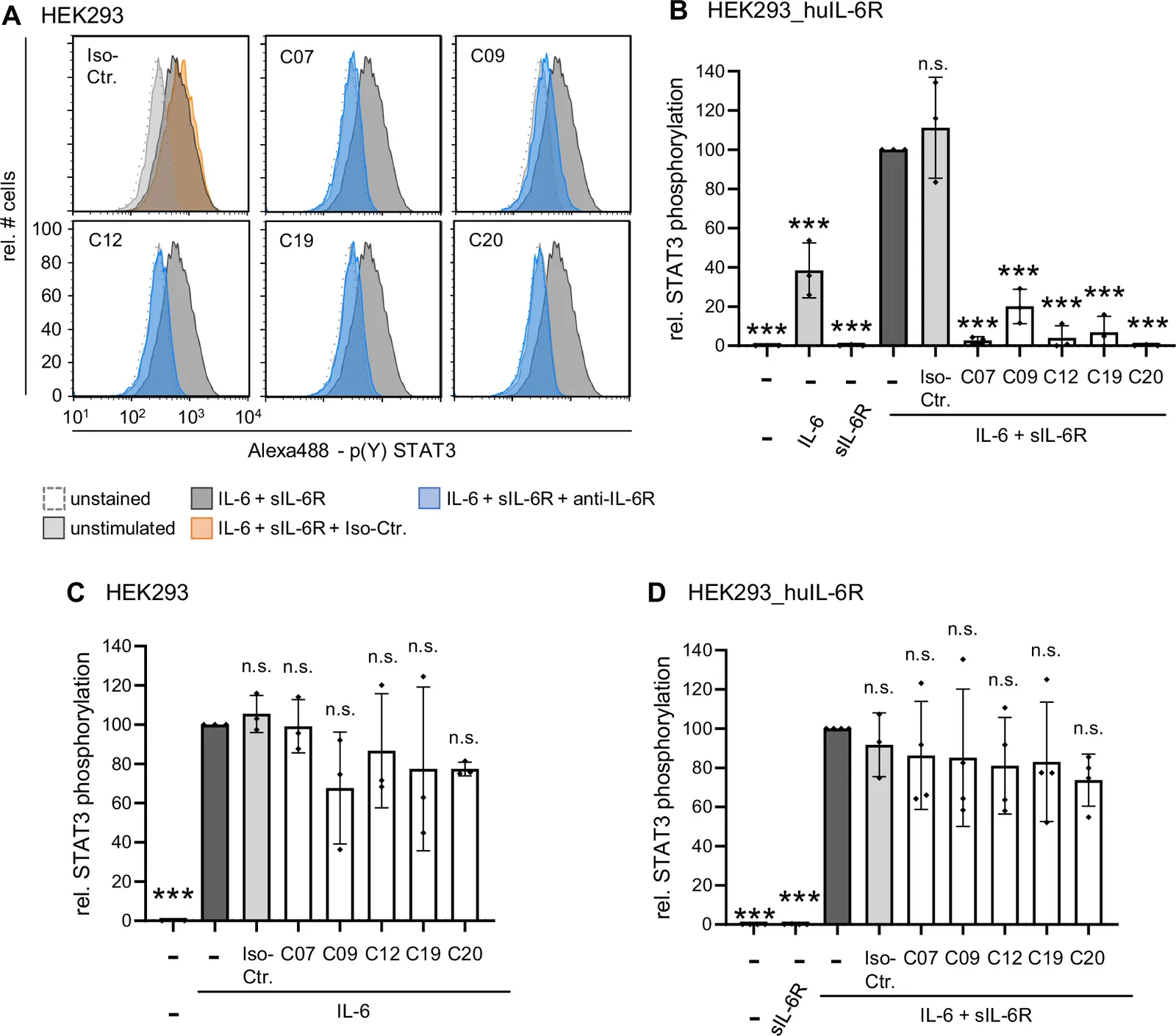
Jak/STAT signalling induced by IL-6 trans-signalling is selectively inhibited by anti-IL-6R mAbs. **A** HEK293 cells were starved for 2 h and subsequently stimulated with a mixture of huIL-6 (10 ng ml^−1^) and husIL-6R (500 ng ml^−1^) for 30 min. Cells were additionally incubated with an IgG isotype control (1H7) (1 µg ml^−1^; 6.7 nM) or with the indicated anti-IL-6R mAb (1 µg ml^−1^; 6.7 nM). STAT3 phosphorylation was evaluated by intracellular flow-cytometry using specific fluorophore-coupled antibodies against (p)Y705 STAT3. Single cells were gated based on SSC-A vs. FSC-A and FSC-H vs. FSC-W dot plots (Supplementary Fig. 5). Representative histograms of *n* = 3 independent experiments are shown. **B** For independent experiments median fluorescence of 10,000 cells per experimental condition was calculated. STAT3 phosphorylation induced by huIL-6 and husIL-6R was set to 100%. HEK293_huIL-6R cells and were starved for 2 h and subsequently stimulated with **C** huIL-6 (10 ng ml^−1^) or **D** husIL-6R (500 ng ml^−1^) alone and a mixture of huIL-6 (10 ng ml^−1^) and husIL-6R for 30 min. Cells were additionally incubated with an IgG isotype control (1H7) or with the indicated anti-IL-6R mAb (1 µg ml^−1^; 6.7 nM). STAT3 phosphorylation was evaluated by intracellular flow-cytometry. For independent experiments median fluorescence of 10,000 cells per experimental condition was calculated. STAT3 phosphorylation induced by huIL-6 was set to 100%. Mean and SD of *n* = 3–4 independent experiments are shown. One-way ANOVA with multiple comparisons: n.s. non-significant, ***p < 0.001. *P*-values indicate differences to cells incubated with cytokine but without mAbs.

HEK293 cells endogenously express gp130 and no IL-6R (Supplementary Fig. 2D). Thus, they mainly respond to trans-signalling. HEK293 cells were incubated with IL-6 and sIL-6R and the specified mAb. STAT3 Y705 phosphorylation was quantified by intracellular flow-cytometry. Trans-signalling results in strong phosphorylation of STAT3 (Fig. 4A). While the IgG isotype control does not affect STAT3 phosphorylation, all anti-IL-6R mAbs strongly reduce trans-signalling-induced STAT3 phosphorylation. C20 and C07, which most strongly reduce trans-signalling-induced proliferation (Fig. 3), also most strongly reduce trans-signalling-induced STAT3 phosphorylation (Fig. 4B).

To render HEK293 cells responsive for classic signalling we stably overexpressed human IL-6R (HEK293_huIL-6R)^51^. HEK293_huIL-6R cells strongly express IL-6R and gp130 (Supplementary Fig. 2E) and stimulation with IL-6 leads to phosphorylation of STAT3. None of the anti-IL-6R mAbs significantly reduces classic signalling in HEK293_huIL-6R cells (Fig. 4C). Finally, STAT3 phosphorylation in HEK293_huIL-6R cells induced by classic signalling in the presence of sIL-6R is not significantly inhibited by the anti-IL-6R mAbs (Fig. 4D).

In summary, the anti-IL-6R mAbs significantly inhibit IL-6 trans-signalling-induced intracellular signalling and proliferation, while they do not reduce classic signalling. C20 and C07 most effectively inhibit trans-signalling-induced proliferation and STAT3 phosphorylation. Since the anti-IL-6R mAb C09 slightly - although not significantly - inhibits classic signalling-induced STAT3 phosphorylation, it was excluded from further analysis.

### IL-11 trans-signalling-induced proliferation is not inhibited by the anti-IL-6R mAbs

**Fig. 5:**
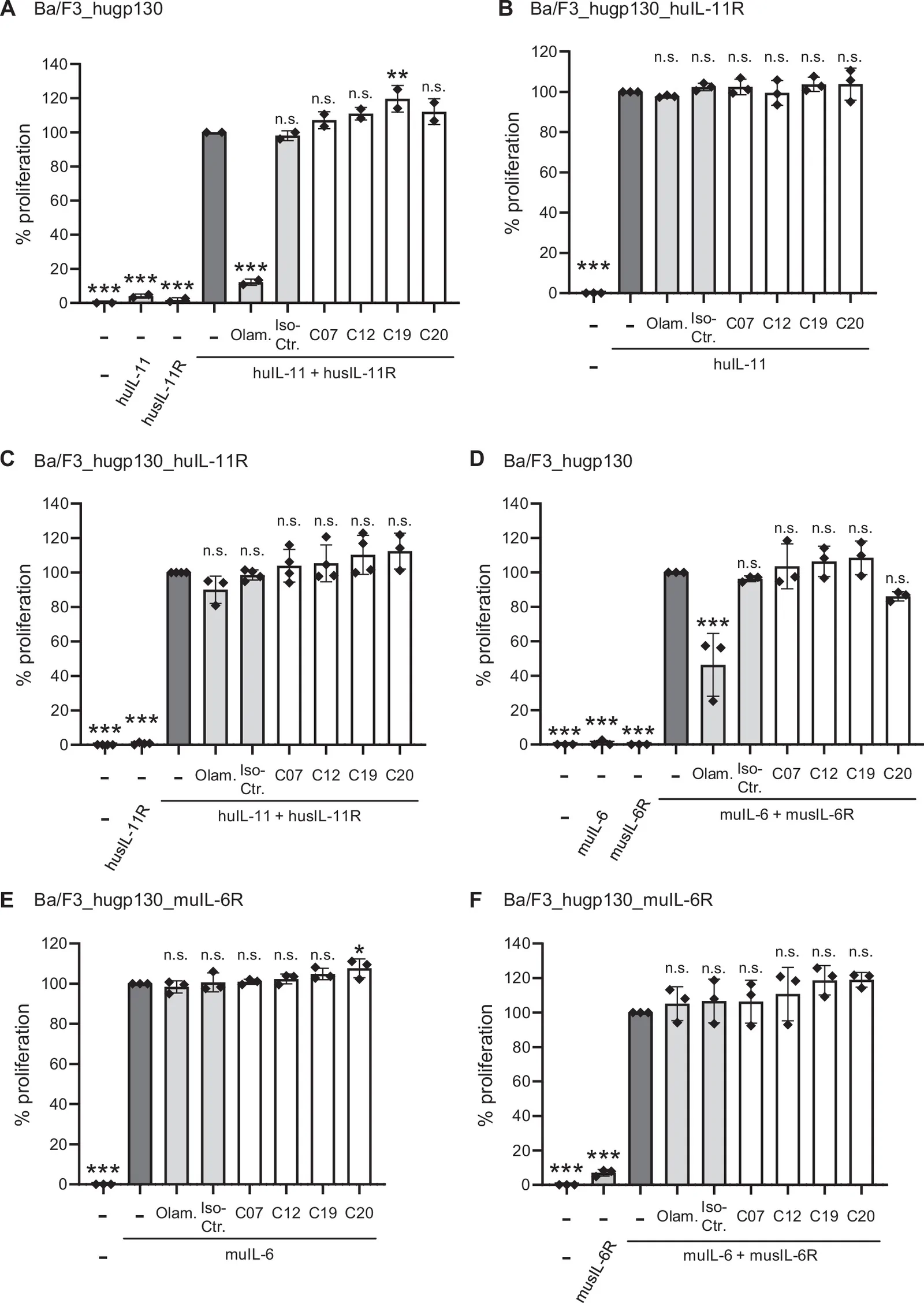
Human IL-11 and murine IL-6 signalling are not affected by the anti-IL-6R mAbs. **A** Ba/F3_hugp130 cells were cultured with huIL-11 (10 ng ml^−1^), husIL-11R (200 ng ml^−1^) or with a mixture of huIL-11 and husIL-11R. Cells were additionally incubated with Olamkicept (10 µg ml^−1^; 41.7 nM), an IgG isotype control (1H7) (10 µg ml^−1^; 66.7 nM) or with the indicated anti-IL-6R mAb (10 µg ml^−1^; 66.7 nM). **B** Ba/F3_hugp130_huIL-11R cells were cultured with huIL-11 (10 ng ml^−1^). Cells were additionally incubated with Olamkicept (10 µg ml^-1^; 41.7 nM), an IgG isotype control (1H7) or with the indicated anti-IL-6R mAbs (10 µg ml^−1^; 66.7 nM). **C** Ba/F3_hugp130_huIL-11R cells were cultured with husIL-11R (200 ng ml^−1^) or with a mixture of huIL-11 (10 ng ml^−1^) and husIL-11R. Cells were additionally incubated with Olamkicept (10 µg ml^−1^; 41.7 nM), an IgG isotype control (1H7) or with the indicated anti-IL-6R mAbs (10 µg ml^−1^; 66.7 nM). **D** Ba/F3_hugp130 cells were cultured with muIL-6 (10 ng ml^−1^), musIL-6R (200 ng ml^−1^) or with a mixture of muIL-6 and musIL-6R. Cells were additionally incubated with Olamkicept (10 µg ml^-1^; 41.7 nM), an IgG isotype control (1H7) or with the indicated anti-IL-6R mAbs (10 µg ml^−1^; 66.7 nM). **E** Ba/F3_hugp130_muIL-6R cells were cultured with muIL-6 (10 ng ml^−1^). Cells were additionally incubated with Olamkicept (10 µg ml^-1^; 41.7 nM), an IgG isotype control (1H7) or with the indicated anti-IL-6R mAbs (10 µg ml^−1^; 66.7 nM). **F** Ba/F3_hugp130_muIL-6R cells were cultured with musIL-6R (200 ng ml^−1^) or with a mixture of muIL-6 (10 ng ml^−1^) and musIL-6R. Cells were additionally incubated with Olamkicept (10 µg ml^-1^; 41.7 nM), an IgG isotype control (1H7) (10 µg ml^−1^; 66.7 nM) or with the indicated anti-IL-6R mAbs (10 µg ml^−1^; 66.7 nM). Viable cells were determined using Cell Titer Blue reagent. The absorbance of cell suspensions grown with cytokine but without mAbs was set to 100%. Mean and SD of *n* = 2–3 independent experiments are shown. One-way ANOVA with multiple comparisons: n.s. non-significant, **p < 0.01, ***p < 0.001. *P*-values indicate differences to cells incubated with cytokine but without Olamkicept or mAbs.

IL-11 signals via a gp130 homodimer, but unlike IL-6, it binds to the IL-11 receptor (IL-11R). IL-11 signalling can be initiated via a membrane-bound IL-11R (classic signalling) or via a sIL-11R (trans-signalling)^52^. Olamkicept also inhibits IL-11 trans-signalling^41^. As human IL-6R and human IL-11R have only a low sequence homology of 24%, the anti-IL-6R mAbs are not expected to interfere with IL-11 signalling. To test this, we analysed the effect of the anti-IL-6R mAbs on IL-11 signalling. The anti-IL-6R mAbs do not reduce IL-11 trans-signalling-induced proliferation of Ba/F3_hugp130 cells, while Olamkicept impairs the proliferation (Fig. 5A). Furthermore, the anti-IL-6R mAbs and Olamkicept do not interfere with IL-11 classic signalling (Fig. 5B) or IL-11 classic signalling in the presence of sIL-11R (Fig. 5C).

This shows that, unlike Olamkicept, the anti-IL-6R mAbs do not affect IL-11-induced trans-signalling.

### Murine IL-6 trans-signalling is not inhibited by the anti-IL-6R mAbs

Mouse models are a valuable tool for investigating the function of antibodies in disease. As both human and murine IL-6R are forming an active receptor-complex with human gp130^40^, we tested whether the anti-IL-6R mAbs cross-react with murine IL-6R.

Ba/F3_hugp130 cells were stimulated with murine IL-6 (muIL-6) and murine sIL-6R (musIL-6R) to initiate trans-signalling. In contrast to the human system, the anti-IL-6R mAbs do not significantly inhibit murine trans-signalling (Fig. 5D). Olamkicept inhibits murine trans-signalling, albeit less efficient than human trans-signalling (Figs. 2A and 5D). This is consistent with a higher IC50 value of Olamkicept for inhibiting trans-signalling in mice than in humans^40^.

Analog experiments were conducted for murine classic signalling in absence or presence of murine sIL-6R. None of the anti-human IL-6R mAbs interfere with murine classic signalling (Fig. 5E, F).

In summary, the anti-human IL-6R mAbs do not affect murine IL-6 signalling.

### The anti-IL-6R mAbs bind to soluble and membrane-bound human IL-6 receptor

**Fig. 6:**
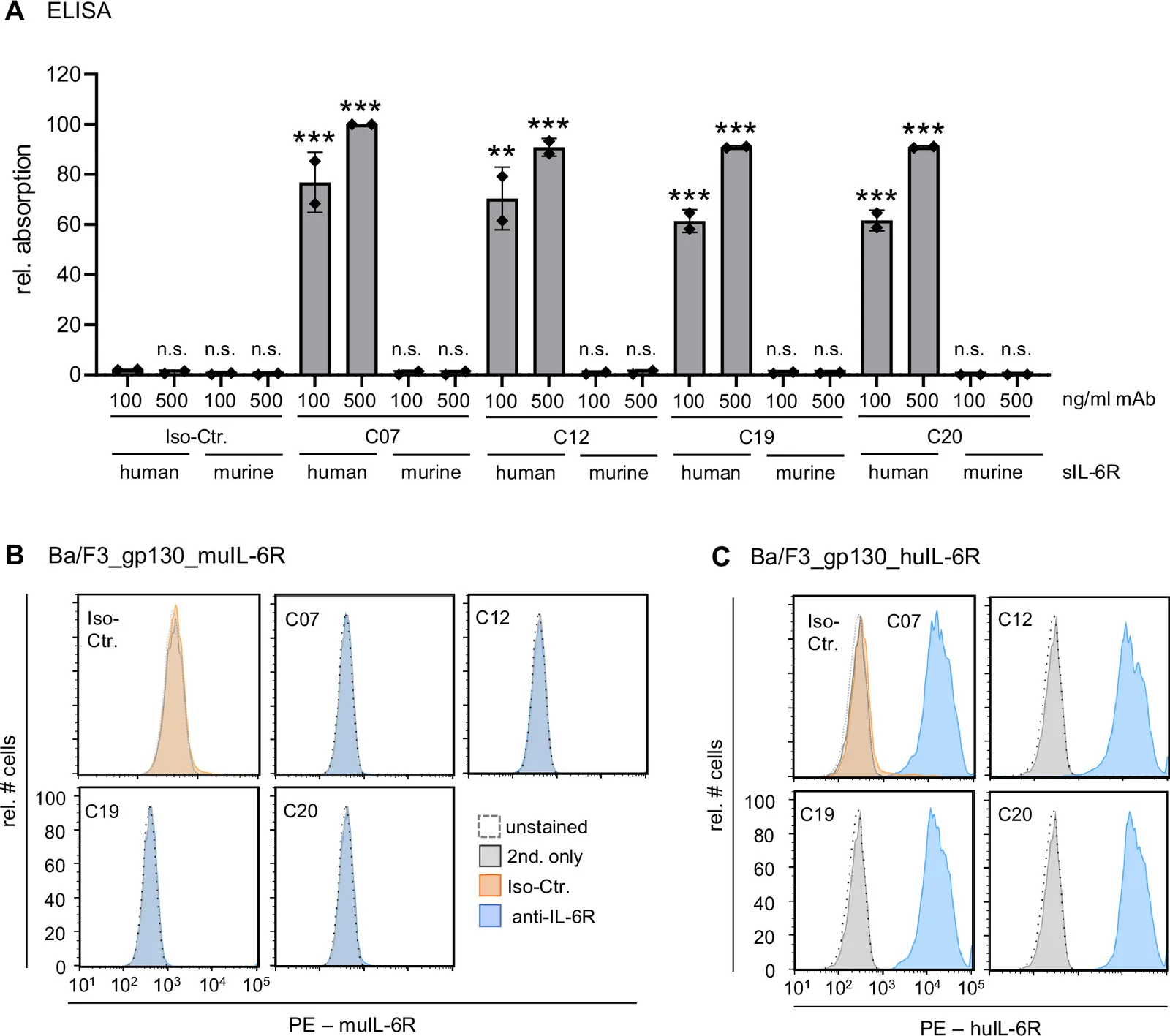
The anti-IL-6R mAbs bind to soluble and membrane-bound human IL-6R. **A** Binding of the IgG isotype control (1H7) (100 and 500 ng ml^−1^; 0.67 nM and 3.33 nM) or the indicated anti-IL-6R mAbs (100 and 500 ng ml^−1^; 0.67 nM and 3.33 nM) to human and murine sIL-6R was determined by ELISA. For independent experiments the highest absorption was normalized to 100%. Representative technical replicates from *n* = 3 independent experiments are shown. One-way ANOVA with multiple comparisons: n.s. non-significant, **p < 0.01, ***p < 0.001. *P* values indicate differences to human sIL-6R incubated with 100 ng ml^−1^ IgG isotype control **B** Ba/F3_hugp130_muIL-6R cells and **C** Ba/F3_hugp130_huIL-6R cells were either incubated with an IgG isotype control (1H7) or with the indicated anti-IL-6R mAb and subsequently stained with specific anti-human-IgG antibodies coupled to PE. Analysis was performed by flow-cytometry. Single cells were gated based on SSC-A vs. FSC-A and FSC-H vs. FSC-W dot plots (Supplementary Fig. 5). Representative histograms of *n* = 3 independent experiments are shown.

The anti-IL-6R mAbs inhibit human IL-6 trans-signalling, while they do not inhibit murine signalling. The lack of inhibition could either be due to a lack of binding of the anti-human IL-6R mAbs to murine IL-6R or due to binding of the mAbs to an epitope that is irrelevant for the formation of an active murine IL-6 receptor-complex. To test for cross-reactivity, binding of the anti-IL-6R mAbs to recombinant human and murine soluble IL-6R was tested by ELISA (Fig. 6A). The IgG isotype control does not bind to human or murine sIL-6R. The anti-IL-6R mAbs bind to human sIL-6R in a dose-dependent manner. In contrast, none of the mAbs bind to murine sIL-6R. In line with lack of cross-reactivity, the human and the murine IL-6R share only an amino acid sequence identity of 53% (Supplementary Note 2). This suggests that the epitopes recognized by the anti-IL-6R mAbs are not present in muIL-6R.

The subtractive immunisation protocol used to generate the anti-IL-6R mAbs was designed to eliminate B-cells producing antibodies against membrane-bound IL-6R, while enriching B-cells producing antibodies against epitopes exclusive for sIL-6R. We thus hypothesised that the anti-IL-6R mAbs selectively inhibit trans-signalling because they bind to an epitope that is present exclusively on human sIL-6R. To test this, binding of the anti-IL-6R mAbs to membrane-bound human and murine IL-6R was investigated by flow-cytometry. Expression of muIL-6R and huIL-6R on Ba/F3_hugp130_muIL-6R and Ba/F3-hugp130_huIL-6R cells was confirmed at functional level by stimulation of the cells with muIL-6 or huIL-6, respectively (Figs. 5E and 2E). The anti-IL-6R mAbs do not bind to membrane-bound muIL-6R (Fig. 6B). However, contrary to our initial hypothesis, all anti-IL-6R mAbs not only bind the soluble (Fig. 6A), but also the membrane-bound huIL-6R (Fig. 6C). Thus, the selective inhibition of trans-signalling cannot be explained by selective binding of the mAbs to sIL-6R. The subtractive immunization protocol did not result in antibodies that bind to an epitope present exclusively on sIL-6R.

### C20 mAb binds to the D2 domain of the human IL-6R with a high affinity

As the selective inhibition of trans-signalling cannot be explained by exclusive binding to sIL-6R, we hypothesised that the anti-IL-6R mAbs block an epitope on human IL-6R that is dispensable for classic, but essential for trans-signalling. To identify this epitope, we focused on C20, as all five analysed mAbs have highly conserved CDRs (Supplementary Fig. 1), and C20 exhibits the lowest IC50 for inhibition of trans-signalling (Fig. 3). To characterise the epitope on IL-6R, we first performed linear epitope mapping. No specific interaction was detected between C20 and IL-6R-derived peptides (Supplementary Note 3, Supplementary Fig. 4), suggesting that C20 recognises a conformational epitope.

**Fig. 7:**
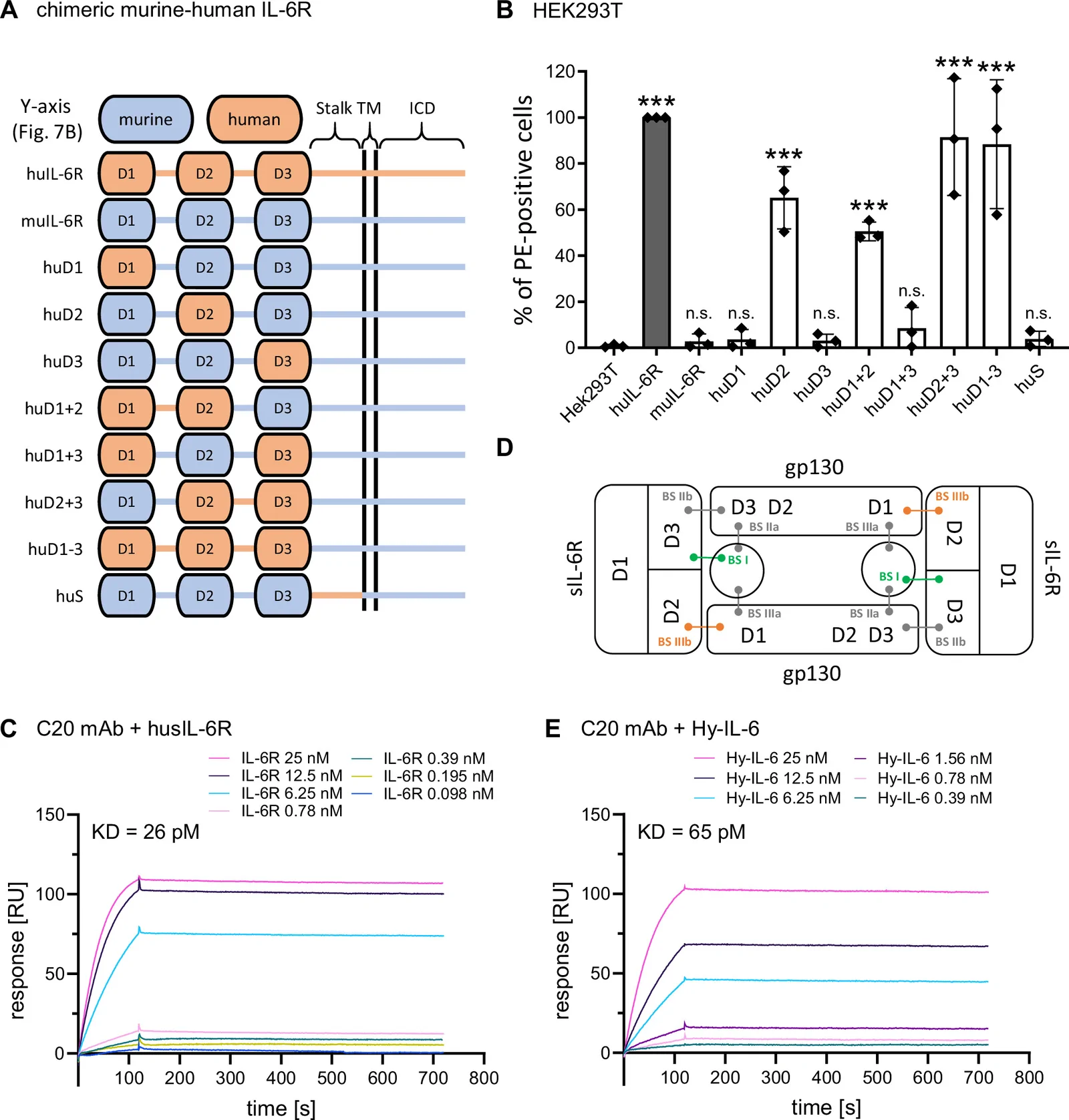
Anti-IL-6R mAb C20 binds with a high affinity to the D2 domain of the human IL-6R. **A** Schematic representation of the murine/human chimeric IL-6R variants (murine (blue), human (orange)). **B** HEK293T cells were transiently transfected with vectors coding for murine/ human chimeric IL-6R variants. As transfection control cells were additionally transfected with a vector coding for GFP (Supplementary Fig. 7). To control the expression of the chimeric receptors, transfected cells were stimulated with IL-6 (Supplementary Fig. 8). Transfected cells were incubated with C20 mAb and subsequently stained with specific anti-human-IgG antibodies coupled to PE. Analysis was performed by flow-cytometry. Single cells were gated based on SSC-A vs. FSC-A and FSC-H vs. FSC-W dot plots (Supplementary Fig. 5). Single transfected HEK293T cells with PE fluorescence intensity comparable to non-transfected HEK293T cells were excluded from the analysis by gating (Supplementary Fig. 6). The amount of HEK293T cells successfully transfected with huIL-6R was set to 100%. Mean and SD of *n* = 3 independent experiments are shown. One-way ANOVA with multiple comparisons: n.s. non-significant, ***p < 0.001. *P*-values indicate differences to untransfected cells. **C** C20 mAb was immobilized on a Protein A chip and increasing concentrations of husIL-6R were injected. Sensorgrams in response units (RU) over time are depicted as coloured lines. Only concentrations used for KD calculation are depicted, all measured concentrations can be found in Supplementary Fig. 9. Results of one experiment are shown. **D** Schematic representation of the interacting domains and binding sites within the hexameric active IL-6 receptor-complex (BS-I (green), BS-IIIb (orange)). **E** C20 mAb was immobilized on a Protein A chip and increasing concentrations of  Hy-IL-6 were injected. Sensorgrams in response units (RU) over time are depicted as coloured lines. Only concentrations used for K_D_ calculation are depicted, all measured concentrations can be found in Supplementary Fig. 12. Results of one experiment are shown.

To narrow down this conformational epitope we exploited the fact that C20 does not bind to muIL-6R (Fig. 6). We transiently expressed chimeric human-mouse IL-6R variants, where parts of the huIL-6R were replaced by the corresponding murine parts^43,53^, in HEK293T cells and analysed binding of C20 to these chimeric receptors using flow-cytometry (Fig. 7A, Supplementary Figs. 5–8).

Compared to non-transfected HEK293T cells and to cells transfected with muIL-6R, binding of C20 is greatly increased in cells transfected with huIL-6R (Fig. 7B). In particular, C20 binds to chimeric receptors that contain a human D2 domain. This suggests that the conformational epitope of C20 is located on the D2 of the huIL-6R.

To determine the affinity of anti-IL-6R mAb C20 for its epitope, we performed surface plasmon resonance (SPR) measurements (Fig. 7C, Supplementary Fig. 9). HusIL-6R binds rapidly to C20 mAb and dissociates extremely slowly. This results in an equilibrium dissociation constant (K_D_) of 26 pM. No interaction of C20 mAb and huIL-6 was detectable (Supplementary Fig. 10). For comparison, Tocilizumab has a K_D_ of 20 nM^54^.

Thus, C20 mAb binds with a high affinity to a conformational epitope in the D2 domain of the huIL-6R. The D2 domain of huIL-6R contains all key amino acids for BS-IIIb that forms the interaction of IL-6R with the D1 of gp130 (Fig. 7D and Supplementary Fig. 11) ^18^. Almost all other key amino acids involved in active IL-6 receptor-complex formation are located within the D3 of IL-6R. The only exceptions are N155 and F187, which are located in D2 and are part of BS-I between IL-6 and sIL-6R^18^. To determine, whether C20 mAb interferes with BS-I, we measured the affinity of Hy-IL-6 to C20 mAb using SPR measurements (Fig. 7E and Supplementary Fig. 12), thereby testing whether the IL-6 moiety in Hy-IL-6 affects affinity of C20 to the IL-6R moiety in Hy-IL-6. Hy-IL-6 like sIL-6R binds rapidly to C20 mAb and dissociates extremely slowly. This results in an equilibrium dissociation constant of 64 pM for Hy-IL-6 binding to C20 mAb. The comparable binding affinities of sIL-6R and Hy-IL-6 suggest that the IL-6 moiety in Hy-IL-6 does not hinder C20 binding to complexed IL-6:IL-6R dimer. Consistently, high amounts of IL-6 do not diminish C20 binding to membrane-bound IL-6R, while IL-6 prevents binding of Tocilizumab to BS-I on IL-6R (Supplementary Fig. 13). This data strongly indicates that the key amino acids of BS-I are not part of the epitope of C20.

In summary, the anti-IL-6R mAb C20 binds with a high affinity to a conformational epitope on the D2 domain of huIL-6R which is distinct to BS-I.

### C20 mAb selectively blocks IL-6 trans-signalling-induced activation of endothelial cells

Classic signalling is limited to a small number of cell types, with hepatocytes being one of the primary physiological target cells. The hepatic APR triggered by classic signalling plays a crucial role in the early defence against invading pathogens. Accordingly, patients treated with Tocilizumab have an increased risk of bacterial infections^55,56^. Maintaining the APR while selectively inhibiting trans-signalling is therefore a key therapeutic goal.

**Fig. 8:**
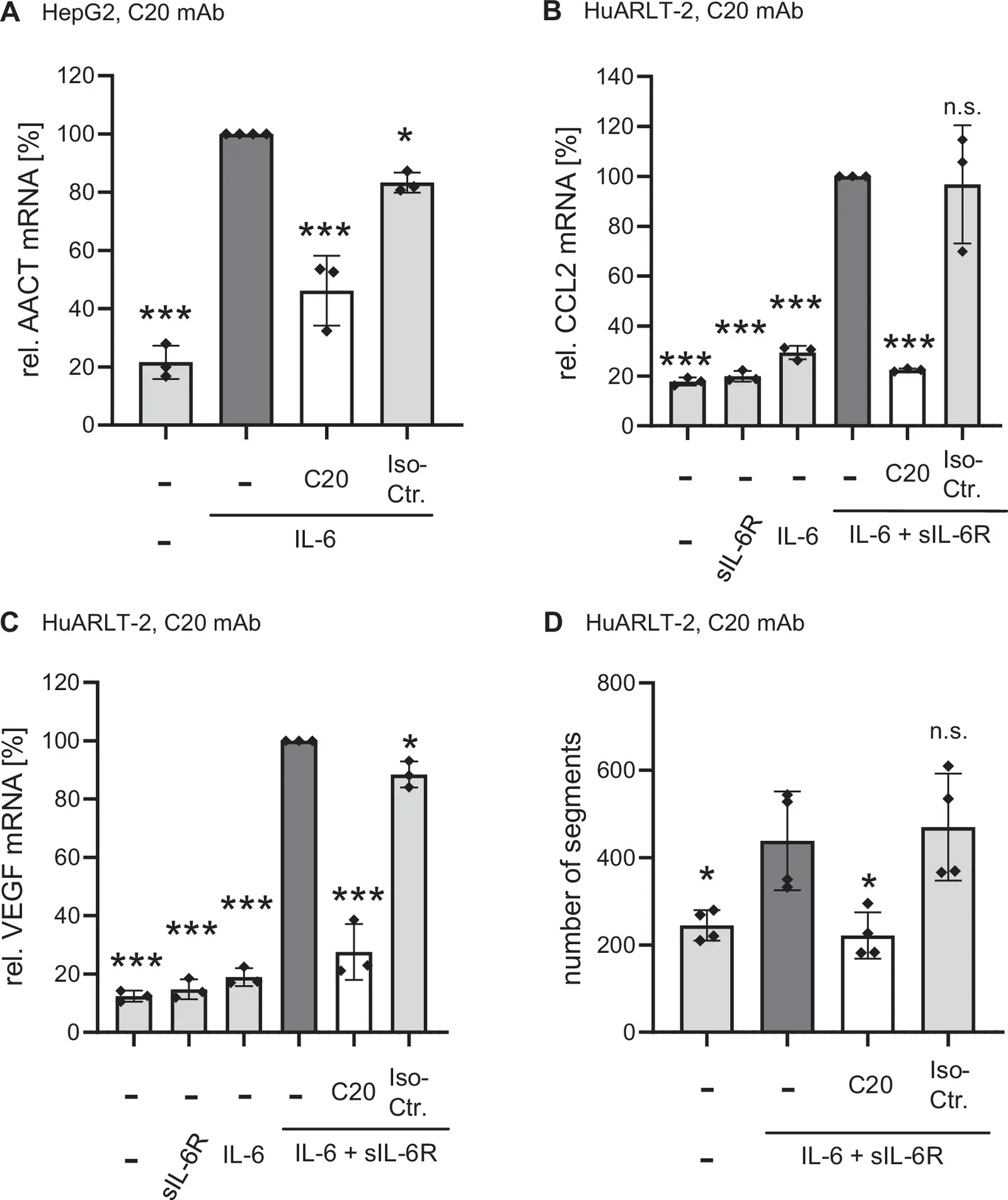
Anti-IL-6R mAb C20 selectively blocks the activation of endothelial cells induced by IL-6 trans-signalling. **A** HepG2 cells were starved overnight and subsequently stimulated with huIL-6 (20 ng ml^−1^) for 24 h. Cells were additionally incubated with anti-IL-6R mAb C20 (200 ng ml^−1^; 1.3 nM) or an IgG isotype control (1H7) (200 ng ml^−1^; 1.3 nM). Total RNA was isolated and transcribed into cDNA. AACT mRNA was quantified by qRT-PCR. AACT mRNA expression in response to huIL-6 was set to 100%. HuARLT-2 cells were stimulated for 24 h with husIL-6R (500 ng ml^−1^), huIL-6 (20 ng ml^−1^) or with a mixture of huIL-6 and husIL-6R. Cells were additionally incubated with anti-IL-6R mAb C20 (200 ng ml^−1^; 1.3 nM) or an IgG isotype control (1H7) (200 ng ml^−1^; 1.3 nM). RNA was isolated and transcribed into cDNA. CCL2 mRNA (**B**) and VEGF mRNA (**C**) were quantified by qRT-PCR. mRNA expression in response to huIL-6 and husIL-6R was set to 100%. Mean and SD of *n* = 3 independent experiments are shown. **D** The ability of HuARLT-2 cells to form capillary-like tubular structures was assessed in a tube formation assay. HuARLT-2 cells were stimulated for 24 h with a mixture of huIL-6 (50 ng ml^−1^) and husIL-6R (500 ng ml^−1^). Cells were additionally incubated with anti-IL-6R mAb C20 (200 ng ml^−1^; 1.3 nM) or an IgG isotype control (1H7) (200 ng ml^−1^; 1.3 nM). Quantification of angiogenic parameters was performed using ImageJ software (Supplementary Fig. 14). Mean and SD of *n* = 4 independent experiments are shown. Control data are in part also shown in Fig. 10D. One-way ANOVA with multiple comparisons: n.s. non-significant, **p < 0.01, ***p < 0.001. *P*-values indicate differences to cells stimulated with cytokine but without mAb.

To assess whether the anti-IL-6R mAb C20 maintains classic signalling in liver cells, we analysed mRNA expression of the acute-phase gene Alpha-1-antichymotrypsin (AACT) in human hepatoma (HepG2) cells. HepG2 cells express membrane-bound IL-6R and gp130 (Supplementary Fig. 2F) and therefore serve as a suitable model for investigating classic signalling. IL-6 induces classic signalling-dependent AACT mRNA expression, which is reduced, but not completely blocked, by C20 mAb (Fig. 8A).

During inflammation, elevated concentrations of IL-6 and sIL-6R in the bloodstream allow systemic trans-signalling. In particular, a cytokine storm can lead to endotheliitis, which poses a significant health risk^57^. Tocilizumab is an effective treatment for cytokine storms^31,58^. Olamkicept reduces the severity of COVID-19-induced endotheliopathy in mice^15^.

We therefore investigated whether C20 mAb inhibits trans-signalling-induced gene expression in endothelial cells. As readouts, we analysed mRNA expression of the chemokine (C-C motif) ligand 2 (CCL2) and vascular endothelial growth factor (VEGF)^59,60^. As an endothelial cell model we used immortalized human umbilical vein endothelial cells (HUVECs), known as HuARLT-2 cells^61^. HuARLT-2 cells express nearly no IL-6R but high levels of gp130 (Supplementary Fig. 2G). Consequently, stimulation with IL-6 alone does not induce CCL2 or VEGF mRNA expression, while stimulation with IL-6 and sIL-6R strongly upregulates expression of both mRNAs (Fig. 8B, C). Notably, IL-6 trans-signalling-induced expression of both CCL2 and VEGF mRNA is strongly inhibited by C20 mAb.

VEGF is a known inducer of angiogenesis^62^. We thus next investigated whether the reduction in VEGF mRNA levels also affects protein function in a tube formation assay. To this end, we measured trans-signalling-induced angiogenesis in the presence and absence of C20 mAb. Stimulating HuARLT-2 cells with IL-6 and sIL-6R results in an increase of angiogenesis measures (Fig. 8D and Supplementary Fig. 14). C20 mAb reduces trans-signalling-induced angiogenesis to the level of unstimulated cells.

Thus, C20 mAb completely blocks the effects of IL-6 trans-signalling on human endothelial cells, while still allowing some classic signalling-dependent hepatic APR.

### Monovalent anti-IL-6R Fab C20 selectively blocks IL-6 trans-signalling

The size and hydrophilicity of mAbs limits their ability to penetrate tissue and thus their therapeutic potential. One strategy to overcome this is to use Fabs, which diffuse more efficiently into tissue^63^ and can even cross the blood-brain barrier^64^. Additionally, due to their smaller size and monovalency, Fabs may also have a different mode of action, especially when interfering with the formation of multi-protein complexes.

We assume that the C20 mAb selectively disrupts the formation of the active trans-signalling receptor-complex. If so, differences in the mode of action between the bivalent C20 mAb and the monovalent C20 Fab are conceivable. To test this, we investigated whether C20 Fab also selectively inhibits trans-signalling.

**Fig. 9:**
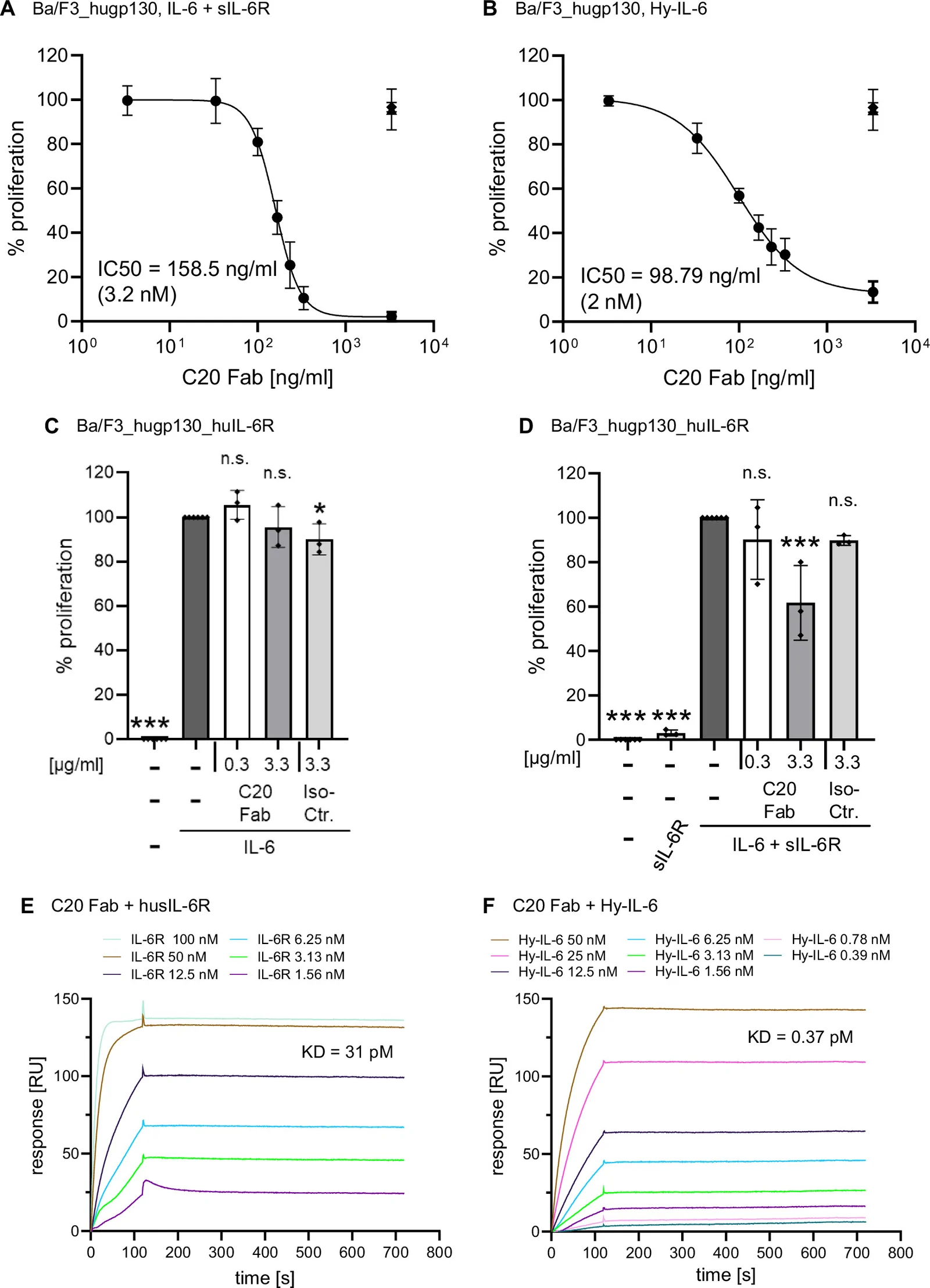
Anti-IL-6R Fab C20 selectively blocks IL-6 trans-signalling. Ba/F3_hugp130 cells were cultured with **A** a mixture of huIL-6 (10 ng ml^−1^) and husIL-6R (200 ng ml^−1^) or **B** with Hy-IL-6 (28 ng ml^−1^) and increasing amounts of the anti-IL-6R Fab C20. Viable cells were determined using Cell Titer Blue reagent. As a negative control cells were incubated with a Fab isotype control (diamond). Inhibition of classic signalling by C20 Fab is depicted as triangle. The absorbance of cell suspensions in the absence of the anti-IL-6R Fab C20 was set to 100%. Mean and SD of *n* = 4 independent experiments are shown. Curves were fitted with a four-parameter non-linear fit. **C** Ba/F3_hugp130_huIL-6R cells were cultured with huIL-6 (10 ng ml^−1^). Cells were additionally incubated with the anti-IL-6R Fab C20 (333 ng ml^-1^ or 3333 ng ml^−1^; 6.67 nM or 66.7 nM) or a Fab isotype control (3333 ng ml^−1^, 66.7 nM). **D** Ba/F3_hugp130_huIL-6R cells were cultured with husIL-6R (200 ng ml^−1^) or with a mixture of huIL-6 (10 ng ml^−1^) and husIL-6R. Cells were additionally incubated with the anti-IL-6R Fab C20 (333 ng ml^−1^ or 3333 ng ml^−1^; 6.67 nM or 66.7 nM) or a Fab isotype control (3333 ng ml^−1^, 66.7 nM). Viable cells were determined using Cell Titer Blue reagent. The absorbance of cell suspensions grown with cytokine but without mAb was set to 100%. Mean and SD of *n* = 3 independent experiments are shown. One-way ANOVA with multiple comparisons: n.s. non-significant, **p < 0.01, ***p < 0.001. *P*-values indicate differences to cells incubated with cytokine but without Fab. C20 Fab was immobilized on a CM5 chip using a Human Fab Capture Kit and increasing concentrations of **E** husIL-6R or **F** Hy-IL-6 were injected. Sensorgrams in response units (RU) over time are depicted as coloured lines. Only concentrations used for K_D_ calculation are depicted, all measured concentrations for (**E**) can be found in Supplementary Fig. 15. For (**F**) all measured concentrations were used for K_D_ calculation. Results of one experiment are shown.

In the following experiments C20 Fab was used in equimolar concentrations to C20 mAb before (Figs. 2 and 3). Ba/F3_hugp130 cells were incubated with IL-6 and sIL-6R together with increasing amounts of C20 Fab. The IC50 for inhibition of trans-signalling was calculated. As negative control cells were treated with a Fab isotype control. The C20 Fab inhibits human trans-signalling with an IC50 of 3.2 nM (Fig. 9A), demonstrating comparable efficacy to C20 mAb (3.5 nM, Fig. 3A). The Fab isotype control does not affect IL-6 trans-signalling-induced proliferation (Fig. 9A, diamond). To validate the selectivity of the anti-IL-6R Fab for trans-signalling, the percentage of classic signalling-induced proliferation of Ba/F3_hugp130_huIL-6R cells in the presence of C20 Fab is shown (Fig. 9A, triangle).

Replacing IL-6 and sIL-6R by Hy-IL-6 resulted in similar IC50 values for C20 Fab, compared to C20 mAb (Figs. 9B and 3F). This suggests that monovalent binding is sufficient to block formation of the active trans-signalling receptor-complex. Also, Hy-IL-6-induced trans-signalling is not affected by the Fab isotype control (Fig. 9B, diamond).

To test the effect of C20 Fab on IL-6 classic signalling, Ba/F3_hugp130_huIL-6R cells were incubated with IL-6 alone or with IL-6 and sIL-6R in the presence or absence of C20 Fab or the Fab isotype control. C20 Fab does not interfere with IL-6 classic signalling (Fig. 9C). C20 Fab in high concentrations slightly reduces classic signalling in the presence of sIL-6R (Fig. 9D).

We next determined the affinity of C20 Fab to human sIL-6R using SPR (Fig. 9E and Supplementary Fig. 15). HusIL-6R binds quickly to C20 Fab and dissociates extremely slowly. This results in a K_D_ of 31 pM for the interaction of C20 Fab with husIL-6R. Thus, C20 mAb (Fig. 7C) and Fab exhibit comparable affinity for husIL-6R. Like C20 mAb, C20 Fab does not interact with human IL-6 (Supplementary Fig. 16). In addition, we determined the affinity for the interaction of C20 Fab with Hy-IL-6 using SPR measurements (Fig. 9F). Hy-IL-6 quickly associates and slowly dissociates from C20 Fab resulting in a K_D_ of <1 pM. This high affinity indicates, that IL-6 does not reduce the affinity of C20 Fab to huIL-6R. A summary of all determined affinities is given in Supplementary Table 3.

The anti-IL-6R Fab C20 effectively and selectively blocks IL-6 trans-signalling with a similarly high affinity as C20 mAb. The bivalence and size of C20 mAb do not appear to be relevant for selective inhibition of trans-signalling. Therefore, C20 Fab could be beneficial for therapeutic applications that require improved tissue distribution.

### C20 Fab selectively blocks endothelial cell activation without affecting acute-phase response

**Fig. 10:**
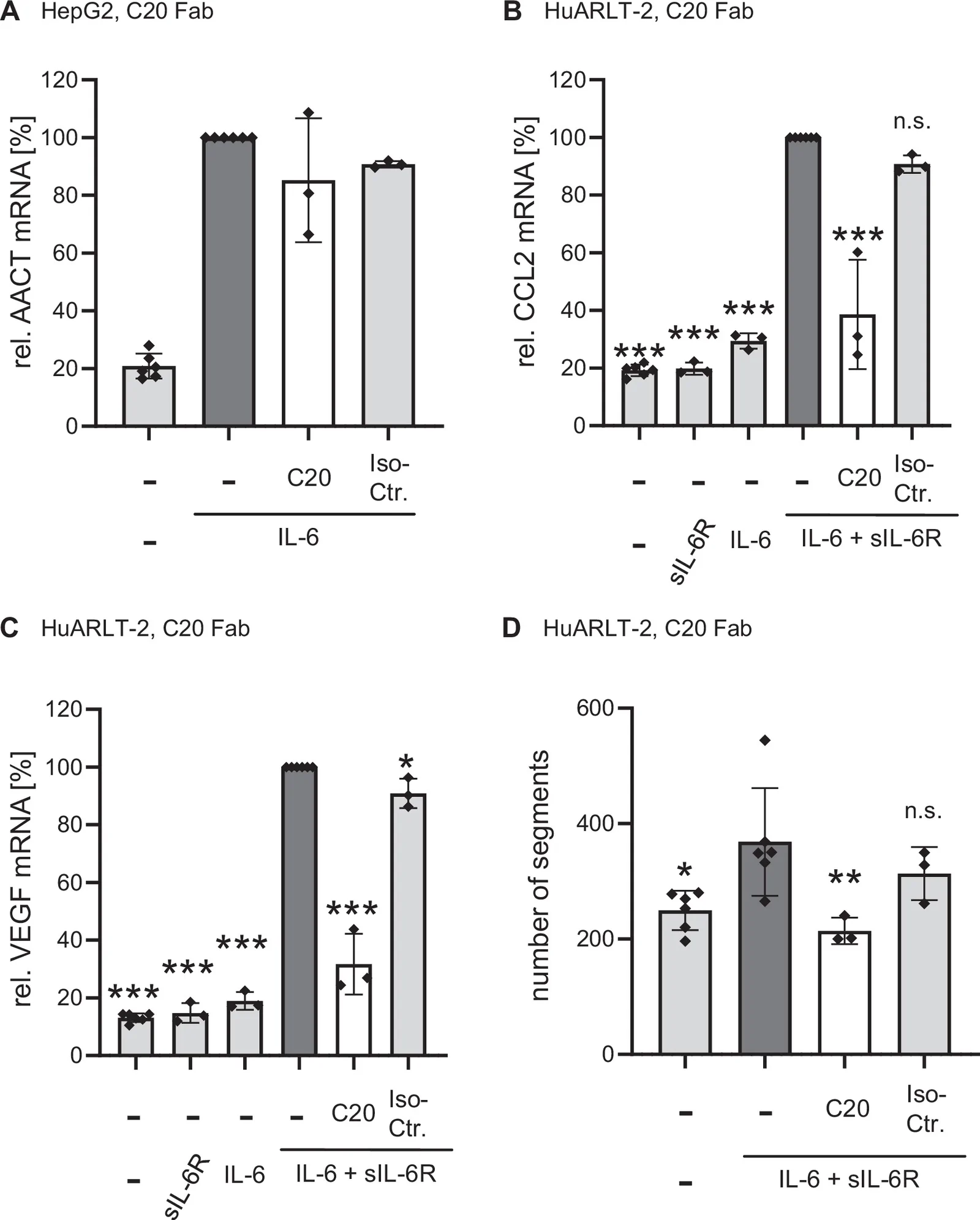
Anti-IL-6R Fab C20 selectively blocks endothelial cell activation without affecting acute-phase response. **A** HepG2 cells were starved overnight and subsequently stimulated with huIL-6 (20 ng ml^−1^) for 24 h. Cells were additionally incubated with anti-IL-6R Fab C20 or a Fab isotype control (66.7 ng ml^−1^; 1.3 nM). Total RNA was isolated and transcribed into cDNA. AACT mRNA was quantified by qRT-PCR. AACT mRNA expression in response to huIL-6 was set to 100%. HuARLT-2 cells were stimulated for 24 h with husIL-6R (500 ng ml^−1^), huIL-6 (20 ng ml^−1^) or with a mixture of huIL-6 and husIL-6R. Cells were additionally incubated with anti-IL-6R Fab C20 (66.7 ng ml^−1^; 1.3 nM) or a Fab isotype control (66.7 ng ml^−1^; 1.3 nM). RNA was isolated and transcribed into cDNA. CCL2 mRNA (**B**) and VEGF mRNA (**C**) were quantified by qRT-PCR. mRNA expression in response to huIL-6 and husIL-6R was set to 100%. Mean and SD of *n* = 3 independent experiments are shown. **D** The ability of HuARLT-2 cells to form capillary-like tubular structures was assessed in a tube formation assay. HuARLT-2 cells were stimulated for 24 h with a mixture of huIL-6 (50 ng ml^−1^) and husIL-6R (500 ng ml^−1^). Cells were additionally incubated with anti-IL-6R Fab C20 (66.7 ng ml^−1^; 1.3 nM) or a Fab isotype control (66.7 ng ml^−1^; 1.3 nM). Quantification of angiogenic parameters was performed using ImageJ software (Supplementary Fig. 17). Mean and SD of *n* = 4 independent experiments are shown. Control data are in part also shown in Fig. 8D. One-way ANOVA with multiple comparisons: n.s. non-significant, **p < 0.01, ***p < 0.001. *P*-values indicate differences to cells stimulated with cytokine but without mAb.

Since we had observed a comparable selectivity, inhibitory effect, and affinity of the C20 Fab and mAb, we investigated how C20 Fab influences APR and whether it blocks activation of endothelial cells. Notably, C20 Fab comparable to the Fab isotype control does not reduce classic signalling-dependent AACT mRNA expression in liver cells (Fig. 10A).

In contrast, trans-signalling-induced CCL2 and VEGF mRNA expression are strongly reduced by C20 Fab in endothelial cells, while the Fab Isotype control hardly affects CCL2 and VEGF mRNA amount (Fig. 10B, C). In line, also trans-signalling induced angiogenesis is blocked by C20 Fab but not by the Fab isotype control (Fig. 10D and Supplementary Fig. 17).

In summary, C20 Fab does not impair acute-phase protein expression, while it blocks IL-6 trans-signalling dependent activation of endothelial cells. This implies that anti-IL-6R Fab C20 is a more versatile candidate for potential therapeutic application compared to C20 mAb.

## Discussion

Global blockade of IL-6 classic and trans-signalling is currently the standard treatment of IL-6-associated diseases^30^. Selective inhibition of trans-signalling offers great potential for reducing or avoiding side effects caused by inhibition of classic signalling in the treatment of inflammatory and proliferative diseases. Selective inhibition of trans-signalling would even allow treatment of IL-6-related diseases in tissues where IL-6 classic signalling is crucial for proper function. To date, no selective inhibitors of human IL-6 trans-signalling other than improved variants of Olamkicept have been described^65,66^. Olamkicept is effective in multiple preclinical models of inflammatory diseases and has successfully passed clinical phase II for treatment of IBD^1,39^. Efforts to develop an anti-IL-6R antibody that selectively blocks human trans-signalling failed^43^.

We describe anti-human IL-6R antibodies that have the unique property of selectively blocking trans-signalling (Fig. 2). These mAbs are highly specific for inhibiting human IL-6 trans-signalling as they do not block murine IL-6 signalling or human IL-11 signalling (Fig. 5).

Contrary to our initial hypothesis, that subtractive immunisation would produce antibodies selective for sIL-6R, the anti-IL-6R mAbs bind to both membrane-bound and soluble huIL-6R (Fig. 6). Thus, the selectivity of the mAbs for inhibiting trans-signalling is not achieved by the mAbs only binding to sIL-6R or the IL-6:sIL-6R complex. A Fab fragment of C20 mAb effectively and selectively blocks trans-signalling (Fig. 9) and exhibits high affinity binding to IL-6R similarly to that of the mAb (Figs. 7C and 9E). Thus, bivalency and size of the C20 mAb are not relevant for selective inhibition of trans-signalling. This suggests, that the mechanism of inhibition is not based on the aggregation of multiple (s)IL-6Rs.

The anti-murine IL-6R mAb 25F10 also binds to both membrane-bound and soluble murine IL-6R, but selectively blocks murine trans-signalling^43^. The authors hypothesised, that the selectiveness of 25F10 indicates differences between the formation of the murine IL-6 receptor-complex in classic and trans-signalling. Human and murine IL-6R share only 54% sequence identity and differ in their 3D structure^44,67^. This is reflected in the fact that an antibody directed against the corresponding epitope of 25F10 within human IL-6R, blocks both human classic and trans-signalling suggesting fundamental differences in the assembly of murine and human IL-6 receptor-complexes^43^.

The C20 mAb binds with high affinity to a conformational epitope located on the D2 of human IL-6R (Fig. 7). The D2 contains all amino acids forming BS-IIIb, which facilitates the interaction of IL-6R with the D1 of gp130^18^. The only other amino acids involved in the formation of the active IL-6 receptor-complex located in D2 are N155 and F187, which are part of BS-I between IL-6 and sIL-6R^18^. C20 mAb and Fab do not bind to human IL-6 (Supplementary Figs. 10, 16) and have the same affinity for both IL-6R (Figs. 7C and 9E) or the IL-6:IL-6R dimer Hy-IL-6 (Figs. 7E and 9F). Furthermore, the presence of high amounts of IL-6 does not reduce binding of C20 mAb to human membrane-bound IL-6R (Supplementary Fig. 13), strongly suggesting that C20 does not interfere with BS-I. It is therefore very likely that C20 interferes with BS-IIIb and thus blocks binding of D2 of IL-6R to D1 of gp130. In line, with C20 not binding to murine IL-6R, the region around BS-IIIb is poorly conserved between humans and mice (Supplementary Note 2).

We thus postulate, that BS-IIIb is not essential for human IL-6 classic signalling. In contrast, BS-IIIb is essential for human trans-signalling, as binding of C20 to D2 of sIL-6R blocks trans-signalling. This suggests that formation of the human active IL-6 receptor-complex differs between classic and trans-signalling. These differences in receptor-complex formation could be due to lower flexibility of the membrane-bound IL-6R compared to soluble IL-6R. The IL-6 receptor-complex, containing membrane-bound IL-6R could be more stable and require only one interaction site (BS-IIb) between IL-6R and gp130 for full activation. In contrast, sIL-6R probably requires two interaction sites with gp130 (BS-IIb and -IIIb) to form a stable, active receptor-complex.

These differences in the formation of the IL-6 receptor-complex for classic and trans-signalling, are of upmost importance for future approaches to selectively inhibit trans-signalling in inflammatory diseases. IL-6 is strongly induced in SARS-CoV-2 patients, accompanied by a long-term increase in sIL-6R^68^. The selective inhibition of IL-6 trans-signalling by Olamkicept is beneficial in reducing SARS-CoV-2-induced endotheliopathy^15^. We show that the trans-signalling-selective C20 mAb and Fab completely block trans-signalling-induced expression of target genes involved in endothelial activation and angiogenesis (Figs. 8 and 10).

IL-6 classic signalling is crucial for defence against invading pathogens. Patients treated with Tocilizumab exhibit an increased risk of bacterial infections, probably due to inhibition of APR^55,56^. Importantly, the expression of the APR gene AACT in hepatocytes is maintained in the presence of C20 mAb and completely unaffected by C20 Fab (Figs. 8 and 10).

C20 Fab, compared to a full-sized antibody, probably has better tissue penetration due to the smaller size of Fabs compared to mAbs^63^. Thus, our data strongly recommend, the therapeutic potential of C20 Fab in the treatment of trans-signalling dependent inflammatory diseases. However, unmodified Fabs are more rapidly eliminated than mAbs within the body^64^. Future in vivo pharmacokinetic and pharmacodynamic studies will be needed to uncover the optimal therapeutic format of C20.

Our findings demonstrate that C20 Fab selectively inhibits IL-6 trans-signalling, not only in cells overexpressing membrane-bound IL-6R, but also in cells that endogenously express membrane-bound IL-6R. HepG2 cells express approximately 2000 membrane-bound IL-6R per cell^26^. Thus, under the experimental conditions used (Fig. 10A), C20 Fab was present at roughly 700-fold molar excess to membrane-bound IL-6R. Together with the high affinity of C20 Fab to IL-6R (Fig. 9E), this suggests that essentially all membrane-bound IL-6R on the HepG2 cells are occupied by C20 Fab. Still, C20 Fab does not reduce IL-6-induced APR. Under trans-signalling conditions (Fig. 10B–D), roughly 7000-fold more sIL-6R is present than membrane-bound IL-6R under classic signalling conditions. Furthermore, the human sIL-6R was present at 10-fold molar excess to C20 Fab. Still, C20 Fab completely blocks IL-6 trans-signalling dependent gene expression and angiogenesis in endothelial cells. These data indicate that the selectivity for trans-signalling is not determined by the absolute number of IL-6R and the ratio of IL-6R to C20, but indeed by distinct properties of the classic and trans-signalling receptor-complexes.

## Conclusion

In summary, human IL-6 classic and trans-signalling rely on distinct receptor-complex assemblies. By exploiting these differences, the anti-IL-6R Fab C20 emerges as a strategy to selectively suppress pathogenic IL-6 trans-signalling while preserving the essential homeostatic and anti-inflammatory functions of IL-6.

## Methods

### Cell culture

Ba/F3_wt^45^ cells were purchased from DSMZ (German collection of Microorganisms and Cell Cultures GmbH) (#ACC300). Ba/F3_hug130^46^, Ba/F3_hugp130_huIL-6R^47^, Ba/F3_hugp130_muIL-6R^53^, and Ba/F3_hugp130_huIL-11R^69^ cells were generated by stable transduction of expression vectors encoding the respective receptors into Ba/F3_wt cells. Ba/F3_hugp130, Ba/F3_hugp130_huIL 6R cells were a generous gift from Prof. Stefan Rose-John (Kiel, Germany). Ba/F3_hugp130_muIL-6R and Ba/F3_hugp130_huIL-11R cells were generated in the lab of C. Garbers.

Ba/F3 variants were grown in DMEM high glucose (Thermo Fisher Scientific, #41966029) supplemented with 10% heat inactivated FCS (Thermo Fisher Scientific, #10270) streptomycin and penicillin (each 100 µg ml^−1^) (Thermo Fisher Scientific, #15140122) at 37 °C in a water saturated atmosphere containing 5% CO_2_. Ba/F3_wt cells were cultured using 10% of IL-3-containing conditioned medium from WEHI-3B-cells (purchased from DSMZ, #ACC26). Ba/F3_hugp130 cells were cultured with 10% of conditioned medium from Hy-IL-6-producing CHO cells (CHO_Hy-IL-6). CHO-Hy-IL-6 cells were a generous gift from Prof. Stefan Rose-John (Kiel, Germany). Hy-IL-6 is a fusion protein of huIL-6 and husIL-6R that induces trans-signalling, but not classic signalling^50^. Conditioned medium containing either IL-3 or Hy-IL-6 was prepared by 3 days culturing of either WEHI-3B or CHO_Hy-IL-6 cells, respectively. Ba/F3_hugp130_huIL-6R cells were cultured with 10 ng ml^−1^ recombinant huIL-6 (Bio-Techne, #7270-IL). Ba/F3_hugp130_muIL-6R cells were cultured with 10 ng ml^−1^ recombinant muIL-6 (Bio-techne, #406-ML). Ba/F3_hugp130_huIL-11R cells were cultured with 10 ng ml^−1^ recombinant human IL-11 (Bio-techne, #218-IL). Recombinant husIL-6R was acquired from Conaris. Recombinant human sIL-11R (#8895-MR) and musIL-6R (#1830-SR) were purchased from Bio-Techne. Olamkicept (#MA5-54954) and Tocilizumab (#MA5-42298, Lot ZA4166981) were purchased from Thermo Fisher Scientific. Hy-IL-6 (#8954-SR) was purchased from Bio-Techne.

To avoid use of cross-contaminated cells, HEK293 (#ACC 305) and HEK293T (#ACC 635) cells were purchased from DSMZ, as HEK cells are listed in the ICLAC register (Version 13, ICLAC-00063). HEK cells were used because they endogenously do not express IL-6R and are thus an appropriate model for trans-signalling. Additionally, HEK cells can be transfected with high efficiency which allows robust expression of murine-human receptor chimeras. HEK293_huIL-6R cells^51^ were generated by stable transduction of an expression vector encoding for human IL-6R into HEK293 cells. HEK293_huIL-6R cells were generated in the lab of A. Dittrich and F. Schaper. HEK293 variants were grown in DMEM high glucose supplemented with 10% heat inactivated FCS, streptomycin and penicillin (each 100 µg ml^−1^) at 37 °C in a water saturated atmosphere containing 5% CO_2_. HEK293_huIL-6R cells were additionally selected with puromycin (2 mg ml^−1^) (Carl Roth GmbH, #0240.4).

To avoid use of cross-contaminated cells, HepG2 cells were purchased directly from DSMZ (#ACC 180), as they are listed in the ICLAC register (Version 13, ICLAC-00310). HepG2 cells were used because they are an established model for human IL-6 classic signalling-induced APR, as in contrast to most other human cell lines they endogenously express both subunits of the IL-6 receptor complex. HepG2 cells were grown in DMEM F12 (Thermo Fisher Scientific, #31331093) supplemented with 10% heat inactivated FCS, streptomycin and penicillin (each 100 µg ml^−1^) at 37 °C in a water saturated atmosphere containing 5% CO_2_.

Immortalised human umbilical vein endothelial cells (HUVECs) HuARLT-2 cells^61^ were a kind gift from Prof. Dagmar Wirth (Braunschweig, Germany). HuARLT-2 cells were seeded on 0.5% gelatine-coated cell culture dishes and cultivated in endothelial cell growth medium (PromoCell, #C-22010) supplemented with 2 µg ml^−1^ doxycycline at 37 °C in a water saturated atmosphere containing 5% CO_2_.

Cells were regularly tested for mycoplasma contaminations and confirmed to be infection-free for all experiments.

### Generation of mAbs and Fabs

The anti-mesothelin mAb 1H7 (IgG isotype control) and the anti-IL-6R mAbs C07, C09, C12, C19 and C20 were generated by MAB Designs Ltd. The anti-mesothelin mAb 1H7 was produced as described in Supplementary Note 4^49,70^. For the generation of anti-IL-6R mAbs this protocol was adapted by immunizing BALB/c mice first with JV1 cells expressing membrane-bound human IL-6R, inducing tolerance with cyclophosphamide and subsequently immunizing with recombinant husIL-6R (Peprotech, #200-06RC). Mice were killed and the spleens were collected. RNA from splenocytes was transcribed to cDNA. cDNA was used for phage-library construction. Phages were panned against husIL-6R. Soluble Fabs were produced from individual *E. coli* clones^71^. In brief, Fab expression was induced and periplasmic extracts containing soluble Fab were extracted by a freeze/thaw cycle. Bacteria were pelleted and frozen at −80 °C for 2 h, then thawed at room temperature and resuspended in 110 ml of PBS. The periplasmic extract was collected after 30 min of shaking at room temperature and subsequent centrifugation. Anti-IL-6R Fabs (C07, C09, C12, C19, and C20) were chimerised at RD Biotech with a human IgG1-expressing mammalian vector and transfected to CHO cells. Cell culture supernatants were collected and antibody concentration was determined. Human Fab isotype control was purchased from Novus Biologicals (#NBP1-97020, Lot 48778).

### Viability assay

Ba/F3 cells were washed three times with PBS. Subsequently, 5000 cells per well were seeded in 96 well culture plates. Cells were incubated in the presence of cytokines, soluble receptors and inhibitors as indicated. Viability of cells was determined using Cell Titer Blue reagent (Promega, #G8080) according to manufacturer’s instructions after 48 h. Absorption at 570 nm and 600 nm was recorded with an Infinite M200 Pro plate reader (Tecan). Curve fitting for calculation of IC50 was done with four parameters least squares fit in GraphPad Prism (version 11.0.2, Dotmatics).

### Flow-cytometry

For intracellular flow-cytometry cells were seeded in 12-well cell culture dishes for 24 h. Subsequently, cells were starved in DMEM high glucose (Thermo Fisher Scientific, #41966029) without FCS for 2 h and treated as indicated in the figures. After stimulation, cells from each well were detached with 150 µl Trypsin per well (Thermo Fisher Scientific, #25300054). 100 µl of the cell suspension were fixed with 100 µl pre-warmed (37 °C) paraformaldehyde (4%) and incubated at 37 °C for 10 min followed by centrifugation (230 × *g*, 4 °C, 5 min). Afterwards, cells were permeabilized by adding 100 µl ice cold methanol (90%) and incubated at −20 °C for 10 min followed by centrifugation (230 × *g*, 4 °C, 5 min). Subsequently, cells were washed twice with cold 1% BSA-EDTA buffer (1% BSA, 0.5 mM EDTA in PBS). Cells were incubated at 4 °C with antibody solution containing (p)Y705 STAT3-Alexa488 coupled antibody (BD, #557814, Lot 2013440, 1:200 in 2% BSA-EDTA buffer (2% BSA, 0.5 mM EDTA in PBS)), overnight. Next, cells were washed three times in 1% BSA-EDTA buffer before flow-cytometric analysis.

10,000 individual cells were measured per condition. Data was recorded on a BD FACS Canto II equipped with 3 lasers (405 nm, 488 nm, 663 nm) using BD FACS Diva Version 10.6.3 (BD).

For surface staining of membrane expressed IL-6R, adherent cells were detached from the cell culture dish using Accutase (Biowest, #L0950-100). Suspension cells were taken directly out of the cell suspension. 300,000 cells were transferred into 5 ml polystyrene tubes (Fisher Scientific, #352052). The cells were centrifuged (230 × *g*, 4 °C, 5 min) and then incubated at 4°C for 30 min in antibody solution (isotype control mAb or anti-IL-6R mAb 1:100 in 1% BSA-EDTA buffer) or in 1% BSA-EDTA buffer. Cells were washed with 3 ml 1% BSA-EDTA buffer and centrifuged (230 × *g*, 4 °C, 5 min). Cells were incubated at 4°C for 30 min in the dark in antibody solution containing PE coupled anti-human IgG Fc (BioLegend, catalogue # 410708, Lot B352999, 1:100 in 1% BSA-EDTA buffer) or in 1% BSA-EDTA buffer. Cells were washed with 3 ml 1% BSA-EDTA buffer before flow-cytometric analysis.

10,000 individual cells were measured per condition. Data was recorded on a BD FACS Canto II equipped with 3 lasers (405 nm, 488 nm, 663 nm) using BD FACS Diva Version 10.6.3 (BD). Data Analyses was performed with FlowJo^TM^ (version: 10.6.1, BD).

### ELISA

ELISA plates were coated with 100 µl neutravidin (5 µg ml^−1^ in PBS) overnight. Plates were washed 3× with 200 µl wash buffer (PBS + 0.05% Tween-20) and blocked with 200 µl PBS + 5% BSA at room temperature for 2 h. Plates were washed 3× with 200 µl wash buffer and incubated with 100 µl recombinant human biotinylated IL-6 (Diaclone, #715H01 CUS; 500 ng ml^−1^ in PBS + 1% BSA) for 1 h. Plates were washed 3× with 200 µl wash buffer and incubated with 100 µl recombinant soluble human or murine IL-6R (500 ng ml^−1^ in PBS + 1% BSA) for 1 h. Plates were washed 3× with 200 µl wash buffer and incubated with the indicated amount of mAbs (100 or 500 ng ml^−1^ in PBS + 1% BSA) in a volume of 100 µl for 1 h. Plates were washed 3× with 200 µl wash buffer and incubated with HRP-coupled anti-human IgG (H + L) (Proteintech, #SA00001-17, Lot U0000119, 1:100 in PBS + 1% BSA) for 1 h. Plates were washed 3× with 200 µl wash buffer. 100 µl of TMB was added to each well. Colour development was stopped after 15 min by adding 100 µl 2 N H_2_SO_4_. Absorption at 450 nm was measured using an Infinite M200 Pro plate reader (Tecan).

### Chimeric receptors

The expression plasmids pcDNA3.1-human IL-6R and pcDNA3.1-murine IL-6R were used as templates for the generation of the IL-6R chimeric constructs. In the D1, D2, D3 and S chimeras, individual extracellular domains of murine IL-6R were replaced by the corresponding human domains. The D1 + D2, D1 + D3 and D2 + D3 chimeras contained two human extracellular domains, whereas the D1–D3 chimera contained all three human extracellular domains. In the S chimera, the stalk region was derived from human IL-6R, while the remaining extracellular domains were derived from murine IL-6R^53^. HEK293T cells were transiently transfected with these expression vectors using lipofectamine2000 (Thermofisher Scientific, #11668027). 24 h later cells were detached with Accutase and stained with anti-IL-6R C20 mAb (1:100 in 1% BSA-EDTA buffer: 1% BSA, 0.5 mM EDTA in PBS) and PE coupled anti-human IgG Fc (Biolegend, #410708, Lot B352999, 1:100 in 1% BSA-EDTA buffer). As transfection control, the cells were co-transfected with a GFP expression vector (Supplementary Fig. 7)^72^. PE and GFP fluorescence intensities were analysed by flow-cytometry. Transfected HEK293T cells with PE fluorescence intensity comparable to non-transfected HEK293T cells were excluded from the analysis by gating. The gating strategy is shown in Supplementary Figs. 5, 6. To verify receptor surface expression an aliquot of the transfected cells was stimulated with human IL-6 (10 ng ml^−1^) and IL-6-induced STAT3 phosphorylation was detected by intracellular flow-cytometry (Supplementary Fig. 8).

### Surface plasmon resonance affinity measurements

SPR experiments were carried out at 25 °C using a BiacoreX 100 (Cytiva) in PBS containing 0.05% surfactant P20 (Cytiva, #BR100054). Anti-IL-6R mAb C20 was immobilized onto a protein A sensor chip (Cytiva, #29127557, Lot #10349531) at a level of ∼220 response units (RUs). Immobilization of anti-IL-6R Fab C20 onto a CM5 sensor chip (Cytiva, #BR100399, Lot #10295121) was performed according to the manufacturer’s instructions of the Human Fab Capture Kit (Cytiva, #28958325) at a level of ∼205 RUs. Recombinant husIL-6R (R&D Systems, #227-SR-025/CF), recombinant huIL-6^73^ or Hy-IL-6^73^ were injected and flowed over the chip surface for 2 min at a flow rate of 30 µl min^−1^. After each binding the sensor chip surface was washed and regenerated with 10 mM glycine-HCl (pH 2.1) for 2 × 1 min at a flow rate of 30 µl min^−1^. The data were analysed with Biacore X100 Control Software (version 2.1.0.201, Cytiva). The graphs were plotted using GraphPad Prism (version 11.0.2).

### qRT-PCR

Total RNA was isolated using the GeneMatrix Universal RNA Purification Kit (EURx, #E3598) according to manufacturer’s instructions. 500 ng of RNA was reverse transcribed into cDNA with the NG dART RT KIT (EURx, #E0801), employing random hexameric primers according to the manufacturer’s instructions. mRNA expression of SERPINA3 (alpha-1-antichymotrypsin, AACT), VEGF, CCL2, GAPDH, and SDHA was analysed with the following primers:

huSERPINA3 fw: 5’- CTTCACCAGCAAGGCTGACC -3’,

huSERPINA3 rv: 5’- GCACAGCCTTATGGACCACC -3’,

huVEGF fw: 5’- GAGGGCAGAATCATCACGAAGT -3’,

huVEGF rev: 5’- TCCTATGTGCTGGCCTTGGTGA -3’,

huCCL2 fw: 5’-CAATCAATGCCCCAGTCACC -3’,

huCCL2 rv: 5’- CCTGAACCCACTTCTGCTTG -3’,

huGAPDH fw: 5’- TGCACCACCAACTGCTTAGC -3’,

huGAPDH rv: 5’- GGCATGGACTGTGGTCATGAG -3’,

huSDHA fw: 5’- TGGGAACAAGAGGGCATCTG -3’,

huSDHA rv: 5’- CCACCACTGCATCAAATTCATG -3’.

GAPDH and SDHA were used as house-keeping genes. qRT-PCR was performed using SG qPCR Master Mix (EURx, #E0401) according to the manufacturer’s instructions. Gene expression was calculated with the following formula

$${ratio}=\,\frac{({E}_{{gene}-{of}-{interest}})^{\Delta} {CP}_{{gene}-{of}-{interest}}({control}-{sample})}{({E}_{{house}-{keeping}-{gene}})^{\Delta}{CP}_{{house}-{keeping}-{gene}}({control}-{sample})},$$

with E being the real-time PCR efficiency and CP being the theoretical real-time-PCR cycle number at which the fluorescence rises above background fluorescence^74^.

### Tube formation assays

HuARLT-2 cells were seeded for 48 h in endothelial growth medium with supplements and 2 μg ml^−1^ doxycycline and then stimulated for 24 h with huIL-6 and husIL-6R. µ-Slide 15 well angiogenesis slides (Ibidi, #81506) were coated with Matrigel (Corning, #354230). Matrigel was thawed slowly on ice before using, dispensed into wells (10 µl per well), and then allowed to polymerize for 30 min at 37 °C. After stimulation, cells were detached with Trypsin, counted and seeded at a density of 7800 cells per well in 50 µl of their respective conditioned supernatants onto µ-Slide 15 well pre-coated with Matrigel. Cells were incubated at 37 °C for 6 h in a water saturated atmosphere containing 5% CO_2_, after which three representative images were taken per well using an AMG EVOS microscope (Thermo Fisher Scientific). Tube formation as represented by number of nodes, segments, junctions and branches was analysed using Image J (version 1.54p)^75^.

### Statistics and reproducibility

All experiments were performed in independent biological replicates. The number of independent biological replicates is given for each figure in the figure caption. Results of individual experiments are shown in the paper indicating reproducibility of the presented data. Additionally, results are presented as means ± SD of independent experiments. Statistical analysis was performed with GraphPad Prism (version 11.0.2) using one-way ANOVA with Tukey´s multiple comparisons test, with the assumption of a normal distribution. The designations n.s. non-significant, *p < 0.05, **p < 0.01, ***p < 0.001 denote *p*-values for the measured differences.

### Reporting summary

Further information on research design is available in the Nature Portfolio Reporting Summary linked to this article.

## Supplementary information

Transparent Peer Review file

Supplementary Material

Description of Additional Supplementary Files

Supplementary Dataset

Reporting Summary

## Data Availability

All data supporting the findings of this study are available within the paper and its Supplementary Information. Source data underlying graphs can be obtained from the file Supplementary Dataset. Should any raw data files be needed in another format they are available from the corresponding author upon reasonable request. Anti-IL-6R antibodies are available from the authors upon signing a material transfer agreement.
