# Supplementary material for: Selective inhibition of IL-6 trans-signalling reveals distinct mechanisms of human classic and trans-signalling receptor-complex formation
Source: Commun Biol. 2026 Oct 14;9:1340. doi: 10.1038/s42003-026-11014-0 (PMC13669321; doi:10.1038/s42003-026-11014-0)
Supplement: Supplementary file 1 [file 42003_2026_11014_MOESM1_ESM.pdf]

nature portfolio

Peer Review File

Selective inhibition of IL-6 trans signalling reveals distinct mechanisms of human classic and trans-signalling receptor-complex formation

Corresponding Author: Dr Anna Dittrich

This manuscript has been previously reviewed at another journal. This document only contains information relating to versions considered at Communications Biology.

This file contains all reviewer reports in order by version, followed by all author rebuttals in order by version.

Version 1:

Reviewer comments:

Reviewer #1

(Remarks to the Author)
This manuscript by Dittrich A. and colleagues is well written on the selective blockade of human IL-6 trans-signaling. The authors reported the selective inhibitory activity of the C20 antibody against human IL-6 trans-signaling, which binds to the D2 region of sIL-6R. However, I ask the author to add the description in the discussion part as follows.

- sgp130-Fc also has selective inhibitory activity for IL-6 trans-signaling. Also, treatment of sgp130-Fc prevents several types of inflammatory diseases, such as inflammatory bowel diseases, which are undergoing clinical trials. It would be better to describe the potential therapy of C20 antibody treatment in inflammatory diseases.

Reviewer #2

(Remarks to the Author)
Authors have considerably improved on the original manuscript and have addressed the majority of previous comments and concerns and have now included 1H7 negative control IgG, validating that inhibition of signalling in Ba/F3 assays with IL-6R mAbs is specific. The dose dependent inhibition is now nicely demonstrated in Ba/F3 assays showing potency ranking of IL-6R IgG. Data show selective inhibition of trans-IL6 signalling mediated through binding to the D2 domain of the IL-6R that interacts with GP130.

The authors then move on from Ba/F3 cells to demonstrate effect of C20 IgG and Fab on classical or trans signalling in more relevant cells types. No negative controls are included in all the assays shown in Fig 7,8 or 9. The authors cannot assume that there will be no impact from adding an irrelevant IgG or Fab to the different assays and therefore the concluding statements associated with each of these figures cannot be validated. It is essential to include a negative control.

The paper is now acceptable subject to two changes:
1. Negative controls must be shown for figures 7,8 & 9.

2. The on-rate of 10e11 for Fab C20 binding binding HyIL6 (Table S9C) is clearly incorrect - probably a typo. The Biacore traces themselves in Fig 6E look to be fine so please check the calculations for on/off rate and overall affinities.

Additional detailed changes required

p2 Line 40 : Add APR
p3 Line 65: "…offering both conceptual and therapeutic advances". This is not a proven therapeutic. Change to "potential therapeutic advances" or reword
p4 line 83-88: "The five binding sites" – would improve reader experience to reference the figure shown in 6d at this point

and change 6d to fig 1
p53 Figure 5: Please clarify y- axis absorption – this seems rather high for OD units, have the values been normalised to a positive control?
P13 line 283: “ a sequence identity of 53%” please clarify that this is amino acid identity
P14 line 309: “Human-mouse IL-6R were parts” Typo: change were to where
P56 Fig 7: Experiment lacks appropriate negative control IgG. Is the reduction in AACT by C20 in Fig 7a due to irrelevant IgG effect in this type of assay? This needs to be tested as different functional assays can be impacted to different extent by irrelevant IgG
P58 Figures A-D: no negative controls included in assays therefore conclusions cannot be validated
P58 Figure 8F and p18 line 399-402 and supp.info 9 (table):Please check the affinity provided for C20 Fab binding Hy-IL-6 using SPR. The Kon is recorded as 10-11 which is not possible or fit with the limitations of the BIAcore X100. In addition the affinity provided is ~ 100 X stronger which also doesn’t make sense.
P60 Fig 9: No Fab negative controls shown alongside C20 therefore conclusions cannot be validated
Conclusion P21 line 481: For balance please consider discussing the impact of using a Fab on PK compared with an IgG

Version 2:

Reviewer comments:

Reviewer #2

(Remarks to the Author)
The authors have addressed all the outstanding comments and the manuscript is now acceptable for publication.

Open Access This Peer Review File is licensed under a Creative Commons Attribution 4.0 International License, which permits use, sharing, adaptation, distribution and reproduction in any medium or format, as long as you give appropriate credit to the original author(s) and the source, provide a link to the Creative Commons license, and indicate if changes were made.
In cases where reviewers are anonymous, credit should be given to 'Anonymous Referee' and the source.
The images or other third party material in this Peer Review File are included in the article’s Creative Commons license, unless indicated otherwise in a credit line to the material. If material is not included in the article’s Creative Commons license and your intended use is not permitted by statutory regulation or exceeds the permitted use, you will need to obtain permission directly from the copyright holder.
To view a copy of this license, visit https://creativecommons.org/licenses/by/4.0/

Authors: *We thank the editors of Nature Communications for their careful evaluation of our manuscript and for recognizing its general interest, as well as for the suggestion to transfer the study to Communications Biology. We are also grateful to the reviewers for their thoughtful and constructive comments. Following the suggestion by the editor of Communications Biology we are resubmitting our paper including novel SPR and flow cytometry data, that show that IL-6 does not interfere with binding of the anti-IL-6R antibodies to huIL-6R. In response to the reviewers' comments, we have revised the manuscript to improve clarity, strengthen the presentation of the results, and provide additional information addressing points related to experimental design, novelty, and interpretation of the findings. Specifically we have extended information on the pro-inflammatory and anti-inflammatory effects of classic and trans-signalling (page 4, lines 73 – 79), added sequence alignments between murine and human IL-6R (new Supplementary Information 6), have included positive and negative controls in the revised figures (2 A-F, 8 A), included molar concentrations of mAbs and Fabs in all figures and added a passage on the molar ratios of antibodies and IL-6R in the discussion section.*
While the scope of the study remains unchanged, we believe that these revisions substantially enhance the comprehensibility of the work and better delineate its advance over prior studies.

Reviewer #1 (Remarks to the Author):
This manuscript (NCMMS-25-89947) describes the generation of a new anti-IL-6 receptor (IL-6R) antibody that specifically inhibits IL-6R trans-signaling and compares its inhibitory activity on IL-6R trans-signaling for tocilizumab and olamkicept. Also, the authors identified the binding site of the new IL-6R antibody on the gp130 domain. Finally, provides the bioactivities of this new antibody using human gp130 and/or human IL-6R in several types of cell lines. Overall, this manuscript provided clear evidence that the new anti-IL-6R antibody has selective inhibitory activities compared to other anti-IL-6R antibodies. The authors concluded the new antibody has selective inhibitory activity with similar patterns to olamkicept but not the same as olamkicept. However, this manuscript has critical concerns. Although the authors showed several in vitro data of selective inhibitory function for IL-6R trans-signaling, the data for pharmacological kinetics analysis should be provided to improve the therapeutic advance of this new antibody. Thus, this manuscript has a rather low novelty.

Authors: *We thank the reviewer for acknowledging that our data provide clear evidence that the presented IL-6R antibody, C20, is a selective inhibitor of IL-6 trans-signalling. While the pro-inflammatory role of trans-signalling has been discussed in the literature for nearly 30 years, to date, no human IL-6 trans-signalling-selective antibody has been reported. Thus, from our knowledge, C20 represents, alongside Olamkicept (sgp130Fc) and variants thereof, the only selective inhibitor of human IL-6 trans-signalling.*
Importantly, C20 exhibits a completely different mode of action from Olamkicept (sgp130Fc) as it binds selectively to IL-6R and interferes with formation of an active IL-6 trans-signalling complex, while Olamkicept traps not only IL-6:sIL-6R complexes but also IL-11:sIL-6R complexes. The binding *of C20 to IL-6R provides superior selectivity against inhibition of other IL-6 type cytokines like IL-11. Additionally, C20 interferes specifically with assembly of the trans-signalling complex which offers novel insights into the mechanisms of IL-6 receptor complex formation.*
We thus believe that both the antibody C20 itself and its unique mechanism of action are of significant interest for understanding IL-6 receptor biology and for targeted anti-IL-6 trans-signalling therapy.
Preclinical studies in mouse models, are challenging because C20 is human-specific (Fig. 4 and 5). While in vivo pharmacokinetic studies in appropriate models will be essential for future clinical translation, these experiments are beyond the scope of the current manuscript. The focus here is on the in vitro characterization and mechanistic insights of C20, which we believe provides an important contribution to the field.

Reviewer #2 :
The authors have identified a small panel antibodies selective for blockade of trans IL-6 signalling (IL6-sIL6R/GP130) over classical signalling (IL-6-memIL-6R), which has therapeutic potential to avoid side effects that arise from blocking the classical pathway.
The manuscript is a mix of antibody characterisation data and hypotheses based on the antibody data regarding how IL-6 signalling pathways can differ at a molecular level.
Overall, the hypothesis that mAb/Fab binds to BS-IIIb on IL-6R is convincing based on the chimeric receptor studies. However, extension of the hypothesis to state that the mAb doesn't interfere with BS-I requires substantiation. Therefore, the manuscript is not acceptable for publication in its current form.
There are a number of major points that need considering if the authors are to consider resubmitting their manuscript at a later date or to another journal.

Authors: *We thank the reviewer for this positive review. We have now included SPR and flow cytometry data showing that binding of C20 is not affected by binding of IL-6 to BS-I (new. Figs. 6E, 8F, S11A and S11B).*

Major Points
1. Antibodies. The described antibody immunisation campaign did not deliver as intended. Only 27 periprep samples were identified (this is typically 100's to 1000's for most campaigns) from which 5 highly related antibodies were identified, probably all derived from the same lineage from the same mouse. In essence there is only a single Ab of interest that was isolated by chance, not by design.

Authors: *The antibodies described in this study were generated using a subtractive immunisation strategy that we previously established (doi:10.1080/19420862.2017.1288770). This approach is specifically designed to eliminate B-cell responses against dominant epitopes, thereby redirecting the immune response toward less immunogenic and functionally distinct epitopes that would likely be missed using conventional immunisation protocols.*
As an inherent consequence of this strategy, a substantial proportion of the antigen-responsive B-cell population is intentionally eliminated, resulting in a markedly smaller pool of antibody-producing clones compared with standard immunisation campaigns. Accordingly, the recovery of fewer periprep samples is expected and does not reflect a failure of the immunisation but rather proves its selective nature.
Furthermore, immunisation campaigns frequently yield multiple highly similar antibodies derived from the same lineage. To avoid redundant downstream screening, we sequenced all recovered antibodies at an early stage and retained only a single representative of each unique sequence. This practice intentionally reduces the apparent number of antibodies for characterization while preserving sequence diversity.
We have now clarified these methodological considerations and expanded the description of the immunisation and selection strategy in the new Supplementary Information 1.

Reviewer #2: 2. Mechanism Data: The data put forward to support selective trans-blockade needs strengthening through the provision of dose response curve data for the test antibodies, positive and negative (1H7 irrelevant mAb) controls in BOTH the Ba/F3 huGP130 and the Ba/F3 huGP130 – huIL-6R proliferation assays with IL-6 or IL-6 + sIL6R. The authors have only shown single mAb concentration data in Figure 1.

Authors: *We agree with the reviewer that dose–response analyses are essential to substantiate selective trans-signalling blockade. In response, we have expanded the presented data with appropriate positive and negative controls. Specifically, new Figure 2 now includes the negative*

control antibody 1H7 (10 µg/ml) in the Ba/F3 hugp130 trans-signalling assay, as well as analyses of the respective anti-IL-6R antibodies in the Ba/F3 hugp130–huIL-6R classic-signalling assay (at 10 µg/ml) at the highest applied concentration used for blockade of trans-signalling. These data demonstrate very low inhibitory activity in the respective non-target pathways, thereby supporting the selectivity of the antibodies.

In addition, we now report IC_{50} values for Olamkicept and Tocilizumab as positive controls (page 10, "In the same experimental setting Tocilizumab and Olamkicept have an IC50 of 0.27 nM and 0.003 nM, respectively"), providing quantitative benchmarks for trans- and classic-signalling inhibition, respectively. Together, these additions strengthen the mechanistic evidence for selective IL-6 trans-signalling blockade.

Reviewer #2: 3. Appropriate controls are missing for some experiments eg IgG negative controls in dose response curves. Reviewer requests that the 1H7 irrelevant mAb control to be included in Figure 2 and other dose response graphs. The same also can be applied to the Fab data in Figure 8 to show dose response curves for the classic signalling, (although for the Fab it has been tested at a higher concentration which is approx.15X in excess of IL-6R which is more convincing data).

Authors: *We agree that the inclusion of appropriate controls is essential for the interpretation of the dose–response data. Accordingly, we have now included the negative IgG control antibody 1H7 in the dose–response curves shown in revised Figure 2. In addition, for the Fab-based experiments, we have incorporated the effect of C20 Fab on classic-signalling as control in the dose–response analyses presented in revised Figure 8. This control further strengthens the conclusion that the observed effects are specific and not attributable to non-specific Fab-mediated activity.*

Reviewer #2: 4. Some experiments need to be repeated. Much higher concentrations of IL-6 are required, 10-100x higher, so that potency curves can be compared to Fig 2 data for trans signalling in Ba/F3 GP130 cells and any impact on the classical response more thoroughly assessed. This is key for the claims being made as the authors have not tested at high enough IL6 concentrations to ensure that there is minimal classical signal inhibition by mabs.

Authors: *We thank the reviewer for the suggestion to vary the ratios of Antibodies, IL-6 and IL-6R to assess potential effects on classical signalling. To clarify, we have now provided detailed information on the molar concentrations of IL-6 and sIL-6R in the figure legends, as well as the absolute numbers of IL-6R molecules expressed on the cell surface:*

page 21: "Our findings demonstrate that C20 Fab selectively inhibits IL 6 trans-signalling, not only in cells overexpressing membrane-bound IL 6R, but also in cells that endogenously express membrane-bound IL 6R. HepG2 cells express approximately 2000 membrane-bound IL 6R per cell. Thus, under the experimental conditions used (Fig. 9A), C20 Fab was present at roughly 700-fold molar excess to membrane-bound IL-6R. Together with the high affinity of C20 Fab to IL-6R (Fig. 8E), this suggests that essentially all membrane-bound IL 6R on the HepG2 cells are occupied by C20 Fab. Still, C20 Fab does not reduce IL-6-induced APR. Under trans-signalling conditions (Fig 9B-D), roughly 7000-fold more shuIL-6R is present than membrane-bound IL 6R under classic signalling conditions (Fig. 9A). Furthermore, the shuIL-6R was present at 10-fold molar excess to C20 Fab (Fig. 9B-D). Still, C20 Fab completely blocks the IL 6 trans signalling dependent gene expression and angiogenesis in endothelial cells. These data indicate that the selectivity for trans-signalling is not determined by the absolute number of IL-6R and the ratio of IL-6R to C20, but indeed by distinct properties of the classic and trans-signalling receptor-complexes."

In summary, *under trans-signalling conditions, the number of sIL-6R greatly exceeds the number of membrane-bound IL-6R under classic signalling conditions, indicating that the observed selectivity of the antibodies cannot be attributed to differences in epitope abundance.*
We have additionally added new data showing that high amounts of IL-6 do not interfere with binding of C20 to membrane-bound IL-6R (new Supplementary Information 11) nor is their affinity reduced binding to preformed dimers of IL-6:IL-6R (Hy-IL-6) (New Fig. 6E and 8F).

.

Reviewer #2: 5. Overall, the hypothesis that mAb/Fab binds to BS-IIIb on IL-6R is convincing based on the chimeric receptor studies. However, extension of the hypothesis to state that the mAb doesn't interfere with BS-I requires substantiation as the concentration of IL-6 (180ng/ml ~ 10 nM) used to compete with C20 (mAb concentration needs to be included, 1:100 dilution is not sufficient information) for binding to IL-6R on cells (supporting information 10) may be way too low to draw this conclusion as C20 is a high affinity mAb which may also be binding avidly to the IL-6R.

Authors:
We have revised the experiment to assess competition between IL-6 and C20 binding using several complementary approaches. First, we included a positive control using Tocilizumab, which is known to block BS-I interactions (new Supplementary Fig. S11A). Second, we increased the concentration of IL-6 to enhance its potential to compete with C20 (new Supplementary Fig. S11B).
In addition, we now report the exact concentrations of IL-6, C20, and Tocilizumab used, providing the necessary quantitative context for interpreting the competition analysis (revised Supporting Information 11).
Furthermore, we have incorporated new SPR measurements demonstrating that both the C20 mAb and its Fab fragment bind the IL-6/sIL-6R fusion protein Hy-IL-6 (new Figs. 6E, 8F) with affinities comparable to those observed for sIL-6R alone (Figs. 6C, 8E).
Collectively, these data strongly support our conclusion that C20 binding to BS-IIIb on human IL-6R does not interfere with IL-6 engagement at BS-I.

Reviewer #2: 6. Additional structural data (solved co-crystal) is required to support the hypothesis for the mAb/Fab blocking only BS-IIIb and not interfering with BS-I and for the proposed distinct receptor complex assemblies.

Authors: *We fully agree with the reviewer that high-resolution structural information, such as a co-crystal of C20 bound to IL-6R within the classic and trans-signalling receptor complexes, would provide valuable mechanistic insight into the selective blockade of BS-IIIb and the distinct receptor assemblies. We note, however, that obtaining structural data for membrane-bound proteins is technically very challenging. To date, the only reported high-resolution structures of the IL-6 receptor complex are based on crystals formed by solubilized gp130, soluble IL-6R, and IL-6 (DOI: 10.1126/sciadv.ade4395). We have however added (new Fig. 6E, 8F, S11A and S11B) to experimentally show that C20 binds independently of BS-I occupation by IL-6.*

Reviewer #2: Minor points
Introduction: Requires more context about the pro-inflammatory and anti-inflammatory effects of the classical and trans- signalling to link it to the utility of a trans- selective mAb/Fab.

Authors: *We have now expanded the Introduction and Discussion to provide additional context on the pro-inflammatory and anti-inflammatory effects of classical and trans-signalling (page 4, lines 73 – 79), highlighting how selective trans-signalling blockade with a mAb or Fab may offer therapeutic*

benefits. The potential utility of this approach has been discussed in the literature for nearly 30 years, and we have referenced relevant studies to support this context (references: 6-15).

Reviewer #2: • Graph consistency: Authors, please keep units on graphs to molar for consistency. It is difficult for the reader to make comparisons of data as its switches between nanograms/mL, micrograms/mL to molar concentrations throughout manuscript.

Authors: *We thank the reviewer for pointing this out. To improve clarity and consistency, we have now included molar concentrations in the legends of all figures.*

Reviewer #2: • Murine cross reactivity: the authors carry out assessment of murine IL-6 signalling (line 247). As the test mAbs do not bind murine IL-6R (Fig 5A) reviewer recommends moving mouse functional assay data to supplementary data and focus this section on binding data as the mouse data distracts the reader from the key points. Also, an alignment of mouse IL-6R and human IL-6R may be useful in supporting epitope recognised by C20 mAb.

Authors: *We appreciate the reviewer's suggestion. However, we have decided to retain the data on the murine IL-6R in the main manuscript, as the lack of cross-reactivity provides important insight into the epitope recognized by C20 and is highly relevant for the design of potential in vivo studies. To further support this, we have now included an alignment of murine and human IL-6R (new Supplementary Information 6), which highlights that the region surrounding BS-IIIb is poorly conserved, consistent with the predicted epitope of C20 on human IL-6R.*

Reviewer #1 (Remarks to the Author):

This manuscript by Dittrich A. and colleagues is well written on the selective blockade of human IL-6 trans-signaling. The authors reported the selective inhibitory activity of the C20 antibody against human IL-6 trans-signaling, which binds to the D2 region of sIL-6R. However, I ask the author to add the description in the discussion part as follows.

Authors: We thank the reviewer for the suggestion to improve discussion of our results.

- sgp130-Fc also has selective inhibitory activity for IL-6 trans-signaling. Also, treatment of sgp130-Fc prevents several types of inflammatory diseases, such as inflammatory bowel diseases, which are undergoing clinical trials. It would be better to describe the potential therapy of C20 antibody treatment in inflammatory diseases.

Authors We have revised the discussion to better place our findings in the context of existing therapeutic strategies targeting IL-6 trans-signalling. Specifically, we now highlight that sgp130-Fc is also a selective inhibitor of IL-6 trans-signalling that has demonstrated therapeutic efficacy in multiple preclinical models of inflammatory disease and is currently being evaluated in clinical trials for inflammatory bowel disease (page 19, lines 429 ff).

Additionally, we specified the potential usage of C20 mAb and Fab in the treatment of inflammatory diseases (page 21, lines 485 ff)

Reviewer #2 (Remarks to the Author):

Authors have considerably improved on the original manuscript and have addressed the majority of previous comments and concerns and have now included 1H7 negative control IgG, validating that inhibition of signalling in Ba/F3 assays with IL-6R mAbs is specific. The dose dependent inhibition is now nicely demonstrated in Ba/F3 assays showing potency ranking of IL-6R IgG. Data show selective inhibition of trans-IL6 signalling mediated through binding to the D2 domain of the IL-6R that interacts with GP130.

Authors: We sincerely thank the reviewer for the positive evaluation of our revised manuscript and for recognizing the improvements made in response to the previous comments.

The authors then move on from Ba/F3 cells to demonstrate effect of C20 IgG and Fab on classical or trans signalling in more relevant cells types. No negative controls are included in all the assays shown in Fig 7,8 or 9. The authors cannot assume that there will be no impact from adding an irrelevant IgG or Fab to the different assays and therefore the concluding statements associated with each of these figures cannot be validated. It is essential to include a negative control.
The paper is now acceptable subject to two changes:
1. Negative controls must be shown for figures 7,8 & 9

Authors: We thank the reviewer for this important comment and agree that appropriate negative controls are essential to demonstrate the specificity of the observed effects. Accordingly, we have included isotype-matched negative control mAbs and Fabs in the experiments presented in Figures 8 (former Fig. 7), 9 (former Fig. 8), and 10 (former Fig. 9). These additional control experiments confirm that the effects observed with C20 mAb and C20 Fab are specific and are not attributable to the addition

of irrelevant IgG or Fab molecules. The corresponding data have been incorporated into the revised Figures 8–10, and the figure legends and results section have been updated accordingly.

2. The on-rate of 10e11 for Fab C20 binding binding HyIL6 (Table S9C) is clearly incorrect - probably a typo. The Biacore traces themselves in Fig 6E look to be fine so please check the calculations for on/off rate and overall affinities.

Authors: We thank the reviewer for carefully identifying this mistake. The reported on-rate in Table S9C contained a typographical error. The correct value is 3.85 × 10-5, and this has now been corrected in the revised Supplementary Table S9C. We have also rechecked the other kinetic parameters and confirm that the Biacore analyses, including the association and dissociation rate constants and the derived binding affinities, are correct.

Additional detailed changes required

Authors: We thank the reviewer for these helpful suggestions and careful reading of the manuscript. We have addressed each point as follows:

p2 Line 40 : Add APR

Authors: "APR" has been added as suggested.

p3 Line 65: "…offering both conceptual and therapeutic advances". This is not a proven therapeutic. Change to "potential therapeutic advances" or reword

Authors: The wording has been revised to "...offering both conceptual and ***potential*** *therapeutic advances."*

p4 line 83-88: "The five binding sites" – would improve reader experience to reference the figure shown in 6d at this point and change 6d to fig 1

Authors: We have introduced a new Figure 1 providing an overview of the five binding sites. Figure 7d (former 6d) has consequently been redesigned with a simplified colour scheme to improve clarity.

P13 line 283: " a sequence identity of 53%" please clarify that this is amino acid identity P14 line 309:

Authors: We have clarified that the reported 53% sequence identity refers to amino acid sequence identity.

"Human-mouse IL-6R were parts" Typo: change were to where

Authors: The typographical error has been corrected.

Conclusion P21 line 481: For balance please consider discussing the impact of using a Fab on PK compared with an IgG

Authors: We have expanded the Discussion to address the pharmacokinetic implications of using a Fab compared with a full-length IgG, including the need for future studies to determine the optimal therapeutic format (page 21, lines 485 ff).

p53 Figure 5: Please clarify y- axis absorption – this seems rather high for OD units, have the values been normalised to a positive control?

Authors: The y-axis label in Fig. 6A (former 5A) has been clarified, and the legend now explains that the absorbance values were normalized to the positive control.

P56 Fig 7: Experiment lacks appropriate negative control IgG. Is the reduction in AACT by C20 in Fig 7a due to irrelevant IgG effect in this type of assay? This needs to be tested as different functional assays can be impacted to different extent by irrelevant IgG

Authors: As detailed above, we have now included IgG controls for all experiments depicted.

P58 Figures A-D: no negative controls included in assays therefore conclusions cannot be validated

Authors: As detailed above, we have now included IgG controls for all experiments depicted.

P58 Figure 8F and p18 line 399-402 and supp.info 9 (table): Please check the affinity provided for C20 Fab binding Hy-IL-6 using SPR. The Kon is recorded as 10-11 which is not possible or fit with the limitations of the BIAcore X100. In addition the affinity provided is ~ 100 X stronger which also doesn't make sense.

Authors: As detailed above, we have corrected this mistake.

P60 Fig 9: No Fab negative controls shown alongside C20 therefore conclusions cannot be validated

Authors: As detailed above, we have now included IgG controls for all experiments depicted.

Reviewer #2 (Remarks to the Author):

The authors have addressed all the outstanding comments and the manuscript is now acceptable for publication.

Authors:

We thank the reviewer.
