# Supplementary material for: Selective inhibition of IL-6 trans-signalling reveals distinct mechanisms of human classic and trans-signalling receptor-complex formation
Source: Commun Biol. 2026 Oct 14;9:1340. doi: 10.1038/s42003-026-11014-0 (PMC13669321; doi:10.1038/s42003-026-11014-0)
Supplement: Supplementary file 2 [file 42003_2026_11014_MOESM2_ESM.pdf]

Supplementary Information

Selective inhibition of IL-6 trans-signalling reveals distinct mechanisms of human classic and trans-signalling receptor-complex formation

Emelie Rieneck[a], Jan René Haak[a,b], Kamal Asgarov[c,d], Jean-René Pallandre[c,e], Nicole Wundrack[a], Julia Ettich[f], Niloufarsadat Miri[a], Doreen M. Floss[f], Jürgen Scheller[f], Christoph Garbers[g], Andy Clark[h], John Wijdenes[h], Fred Schaper[a, i, j,k] and Anna Dittrich[a, i, j,k*]

- *a Otto von Guericke University Magdeburg, Institute of Biology, Chair of Systems Biology, Universitätsplatz 2, 39106 Magdeburg, Germany*
- *b present affiliation: Qpliance GmbH, Marie-Curie Straße 3, 14656 Brieselang, Germany*
- *c INSERM U1098, Université de Franche-Comté, 8 Rue du Docteur Jean-François Xavier Girod, 25020 Besançon Cedex, France*
- *d present affiliation: RNACoreX, 5 Rue Lepenot, 25720 Avanne-Aveney, France*
- *e present affiliation: Etablissement Français du Sang - Bourgogne Franche-Comté (EFS BFC), 8 rue du Docteur Girod, 25020 Besançon Cedex, France*
- *f Institute for Biochemistry and Molecular Biology II, Medical Faculty and University Hospital Düsseldorf, Heinrich Heine University Düsseldorf, Universitätsstraße 1, 40225 Düsseldorf, Germany*
- *g Institute of Clinical Biochemistry, Hannover Medical School, Carl-Neuberg-Strasse 1, 30625 Hannover, Germany*
- *h Mabdesigns Ltd, 20 Balderton Street, W1K 6TL London, UK*
- *i Center for Dynamic Systems: Systems Engineering (CDS), Universitätsplatz 2, 39106 Magdeburg, Germany*
- *j Magdeburg Center for Systems Biology (MACS), Universitätsplatz 2, 39106 Magdeburg, Germany*
- *k Center for Health and Medical Prevention (CHaMP), Universitätsplatz 2, 39106 Magdeburg, Germany*

* corresponding author:
Otto von Guericke University Magdeburg, Institute of Biology, Chair of Systems Biology, Universitätsplatz 2, 39106 Magdeburg, Germany, anna.dittrich@ovgu.de

Supplementary Note 1: Generation of anti-IL-6R antibodies

To generate the anti-IL-6R antibodies described in this paper a subtractive immunization protocol was used. Mice were first immunized with JV1 cells expressing membrane-bound human IL-6R. Subsequently, mice were treated with cyclophosphamide to obtain tolerance to membrane-bound IL-6R. Following four cycles of immunization and depletion, mice were immunized with sIL-6R**.** Subsequently, mice were killed and total RNA was isolated from splenocytes. As an inherent consequence of this strategy, a substantial proportion of the antigen-responsive B-cell population is intentionally eliminated, resulting in a markedly smaller pool of antibody-producing clones compared to standard immunisation campaigns. To avoid redundant downstream screening, all recovered antibodies were sequenced at this step and 27 representatives of each unique sequence were used for further characterization. Next, a fragment antigen-binding region (Fab) library was created using phage display technology. Fab-containing periplasmic extracts were collected from the 27 single colony cultures out of this Fab library. The first screening procedure involved identifying those Fabs that were most likely to selectively interfere with IL-6 trans-signalling. To this end, periplasmic extracts, each containing one of the 27 Fabs, were tested to inhibit IL-6 trans- and classic signalling-induced cell proliferation and STAT3 phosphorylation.

Proliferation of the murine pro B cell line Ba/F3 strongly depends on stimulation with the cytokine IL-3. As Ba/F3 wildtype (Ba/F3_wt) cells neither express gp130 nor IL-6R, they do not respond to IL-6. However, stable expression of human gp130 (Ba/F3_hugp130) and human IL-6R (Ba/F3_hugp130_huIL-6R) allows IL-6-dependent proliferation in the absence of IL-3. Ba/F3_hugp130_huIL-6R and Ba/F3_hugp130 cells were stimulated with human IL-6 (10 ng ml^{-1}) together with sIL-6R (200 ng ml^{-1}) to induce classic and trans-signalling, respectively. Where indicated, cells were additionally incubated with 1 % of the 27 periplasmic extracts.

Control cells were incubated without cytokines or with IL-6 and sIL-6R alone. The number of viable cells was determined after 48 h. Next, the number of viable cells incubated with IL-6 and sIL-6R was set to 100 % and the percentage of viable cells in the presence of the Fabs was calculated (Supplementary Table 1A). All 27 anti-IL-6R Fabs analysed inhibit IL-6-induced proliferation of Ba/F3_hugp130_huIL-6R and Ba/F3_hugp130 cells. However, the strength of the inhibition varies between the different Fabs. For example, Fabs 01, 20, and 26 reduce trans-signalling-induced proliferation by more than 50 percent, while Fabs 08 and 23 do so only to a small extent. Next, we evaluated the Fabs according to their ability to preferentially interfere with IL-6 trans-signalling. The ratio between the residual proliferation of Ba/F3_hugp130_huIL-6R cells in the presence of the Fabs and the residual proliferation of Ba/F3_hugp130 cells in the presence of the Fabs was calculated. Fabs that inhibit trans-signalling stronger than classic signalling are characterized by a high ratio. Accordingly, the 27 Fabs were sorted in descending order according to this ratio. Based on this list, Fabs 20, 07, 19, 09 and 12 are the most promising candidates as selective inhibitors of trans-signalling.

Second, the ability of the 27 candidate Fabs to preferentially block trans-signalling-induced Jak/STAT signalling was characterized, which serves as a more direct measure of classic and trans-signalling than proliferation. Two variants of human embryonic kidney cells (HEK293) were used to monitor STAT3 activation. HEK293 cells express endogenous gp130, but nearly no membrane-bound IL-6R and thus serve as a model for IL-6 trans-signalling. HEK293_huIL-6R cells were derived from HEK293 cells by stable overexpression of human IL-6R. HEK293_huIL-6R cells serve as a model for classic signalling. HEK293_huIL-6R cells and HEK293 cells were treated with IL-6 (10 ng ml^{-1}) together with sIL-6R (200 ng ml^{-1}) to induce classic signalling and trans-signalling, respectively. Where indicated, 1 % of the periplasmic extracts

containing anti-IL-6R Fabs were added. As a control, cells were left untreated or treated with IL-6 and sIL-6R alone. After stimulation, STAT3 Y705 phosphorylation was determined by intracellular flow-cytometry. Fluorescence of cells stimulated with IL-6 and sIL-6R was set to 100 % and the percentage of STAT3 phosphorylation in the presence of each Fab was calculated (Supplementary Table 1B).

All 27 anti-IL-6R Fabs analysed inhibit IL-6 trans-signalling-induced STAT3 phosphorylation by more than 40 %. In contrast, none of the tested Fabs inhibit STAT3 phosphorylation in response to classic signalling in HEK293_huIL-6R cells by more than 40 %. To quantify the specificity of each Fab for inhibiting trans-signalling, the ratio between residual STAT3 activation in HEK293_huIL-6R cells (classic signalling) and in HEK293 cells (trans-signalling) in the presence of the anti-IL-6R Fabs, was calculated. The Fabs were sorted in descending order of this ratio. Remarkably, the same five Fabs 20, 19, 07, 09, and 12 ranked among the first Fabs in both, the ranking based on the inhibition of IL-6-induced proliferation and in IL-6-induced STAT3 activation (compare supplementary Tables 1A and 1B).

Supplementary Table 1: Screening for anti-IL-6R Fabs that preferentially interfere with IL-6 trans-signalling.

A) 5,000 Ba/F3_hugp130_huIL-6R or Ba/F3_hugp130 cells were cultivated for two days with huIL-6 (10 ng ml^{-1}) together with husIL-6R (200 ng ml^{-1}) to enable classic and trans-signalling-induced proliferation. Where indicated, cells were additionally incubated with 1 % of periplasmic extract containing the specified anti-IL-6R Fab (C01-27). The number of viable cells was determined using Cell Titer Blue reagent after 48 h. The absorbance of cell suspensions grown with cytokines in the absence of Fabs was set to 100 %. Mean values of n = 4 independent experiments are shown. To rank the Fabs according to their ability to interfere with trans-signalling, the ratio between the remaining proliferation induced by classic signalling and the remaining proliferation induced by trans-signalling in the presence of the analysed Fabs was calculated. Orange indicates high values of this ratio, while blue indicates low values of this ratio.

B) HEK293_huIL-6R or HEK293 cells were starved for 2 h and subsequently stimulated for 30 min with huIL-6 (10 ng ml^{-1}) together with husIL-6R (200 ng ml^{-1}) to enable classic and trans-signalling-induced STAT3 phosphorylation. Where indicated, cells were additionally incubated with 1 % of periplasmic extract containing the specified anti-IL-6R Fab (C01-27). STAT3 phosphorylation was evaluated by intracellular flow-cytometry using a specific fluorophore-coupled antibody against (p)Y705 STAT3. For independent experiments mean fluorescence of 10,000 cells per experimental condition was calculated. Mean values of n = 4 independent experiments are shown. STAT3 phosphorylation in cells stimulated with cytokines was set to 100 %. To rank the Fabs according to their ability to interfere with trans-signalling, the ratio between the remaining STAT3 phosphorylation induced by classic signalling and the remaining STAT3 phosphorylation induced by trans-signalling in the presence of the analysed Fabs was calculated. Orange indicates high values of this ratio, while blue indicates low values of this ratio.

A

Fab	Classic signalling induced proliferation [%]	Trans-signalling induced proliferation [%]	Ratio
without	100	100	1.0
**C20**	82	37	2.2
**C07**	86	56	1.5
**C19**	73	53	1.4
**C09**	92	70	1.3
**C12**	62	48	1.3
C27	62	51	1.2
C01	53	44	1.2
C25	70	58	1.2
C14	73	61	1.2
C26	41	35	1.2
C18	69	60	1.2
C17	85	74	1.2
C16	54	49	1.1
C05	74	67	1.1
C10	68	62	1.1
C02	76	69	1.1
C15	72	67	1.1
C03	66	61	1.1
C11	81	76	1.1
C21	61	59	1.0
C04	68	66	1.0
C13	69	67	1.0
C23	85	84	1.0
C08	90	90	1.0
C24	73	76	1.0
C06	55	59	0.9
C22	54	58	0.9

B

Fab	Classic signalling induced STAT3 phosphorylation [%]	Trans-signalling induced STAT3 phosphorylation [%]	Ratio
without	100	100	1.0
**C20**	97	32	3.0
**C19**	72	24	3.0
**C07**	77	28	2.8
**C26**	94	39	2.4
**C09**	83	38	2.2
**C12**	90	42	2.1
C25	88	42	2.1
C18	89	46	1.9
C17	71	38	1.9
C10	100	54	1.8
C21	80	45	1.8
C22	72	42	1.7
C16	80	46	1.7
C27	82	51	1.6
C08	93	58	1.6
C14	86	55	1.6
C01	85	55	1.5
C15	72	48	1.5
C04	79	54	1.5
C13	80	55	1.5
C06	80	57	1.4
C11	85	60	1.4
C05	75	54	1.4
C24	70	54	1.3
C02	74	59	1.3
C23	68	57	1.2
C03	63	54	1.2

Supplementary Figure 1

Heavy chain

	Nr.	Sequence
CDR1	C07	GFTFSSYA
	C09	GFTFSNYG
	C12	GFTFSNYG
	C19	GFTFSNYG
	**C20**	**GFTFSNYG**

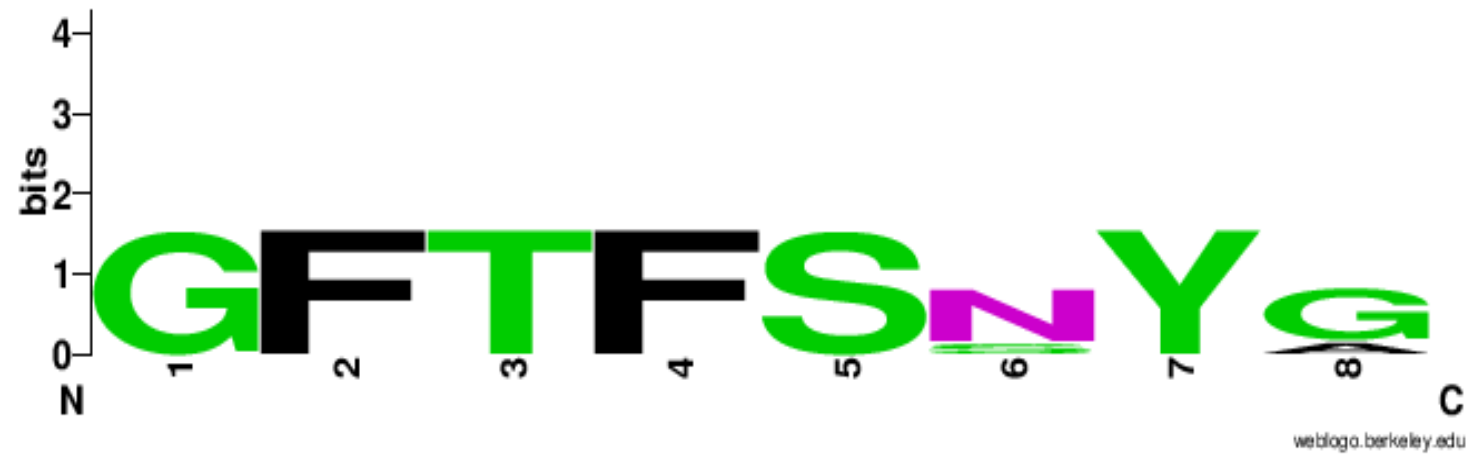

	Nr.	Sequence
CDR2	C07	INSNGGST
	C09	INSNGGST
	C12	INSNGGST
	C19	MNSKGGST
	**C20**	**INSNGGST**

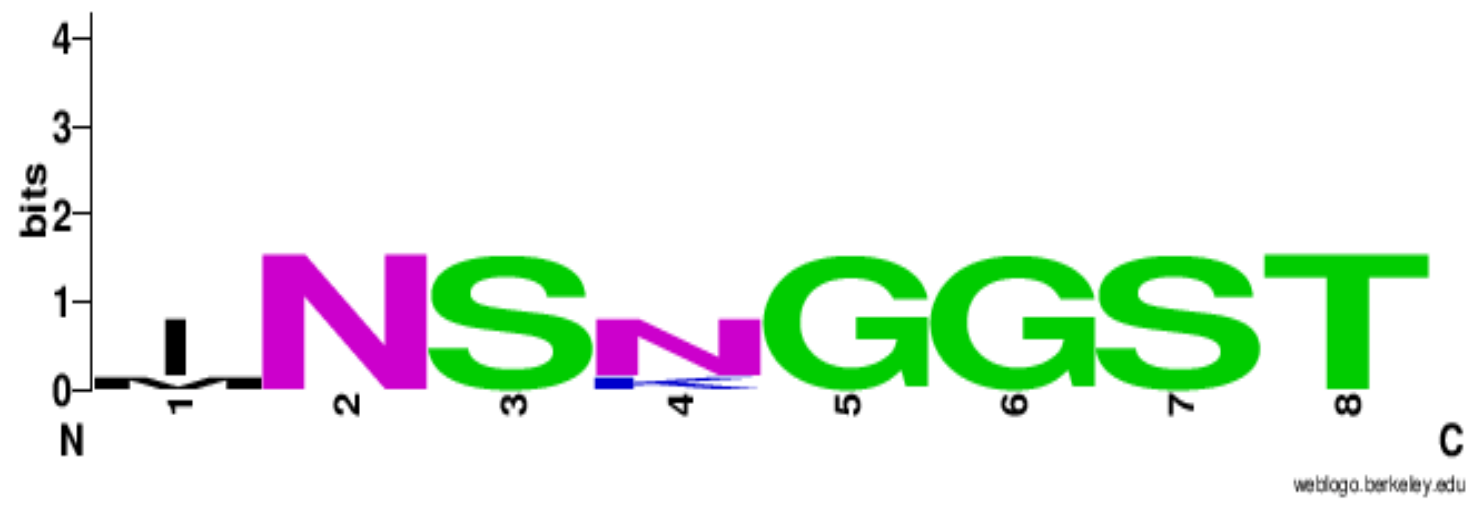

	Nr.	Sequence
CDR3	C07	AREGYYTMDY
	C09	ARDGNYVSDY
	C12	TRDGNFVSDY
	C19	ARDGYYTMDY
	**C20**	**ARDGNFVSDY**

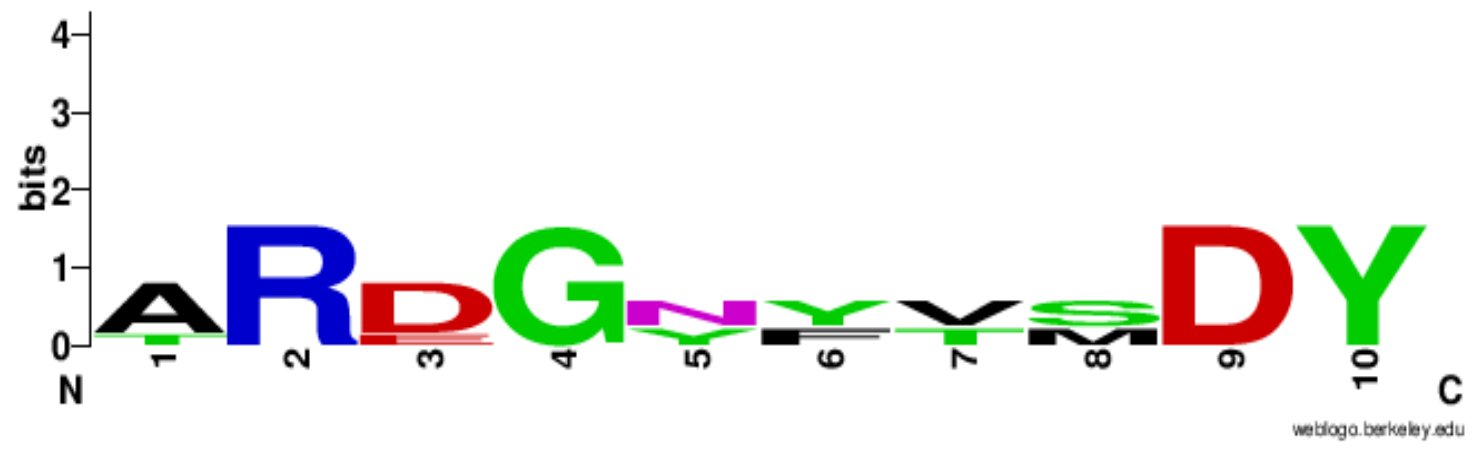

Light chain

	Nr.	Sequence
CDR1	C07	ESVDSYGNSF
	C09	ESVDSYGNSF
	C12	ESVDSYGNSF
	C19	KSVDSFGNSF
	**C20**	**ESVDSYGNRF**

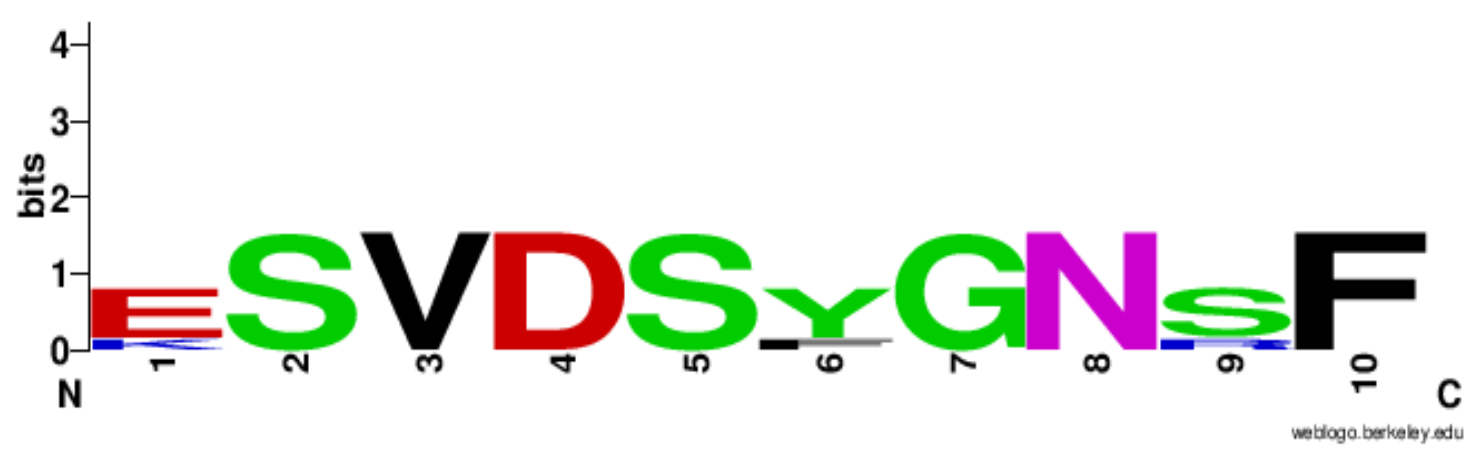

	Nr.	Sequence
CDR2	C07	LAS
	C09	LAS
	C12	LAS
	C19	LAS
	**C20**	**LAS**

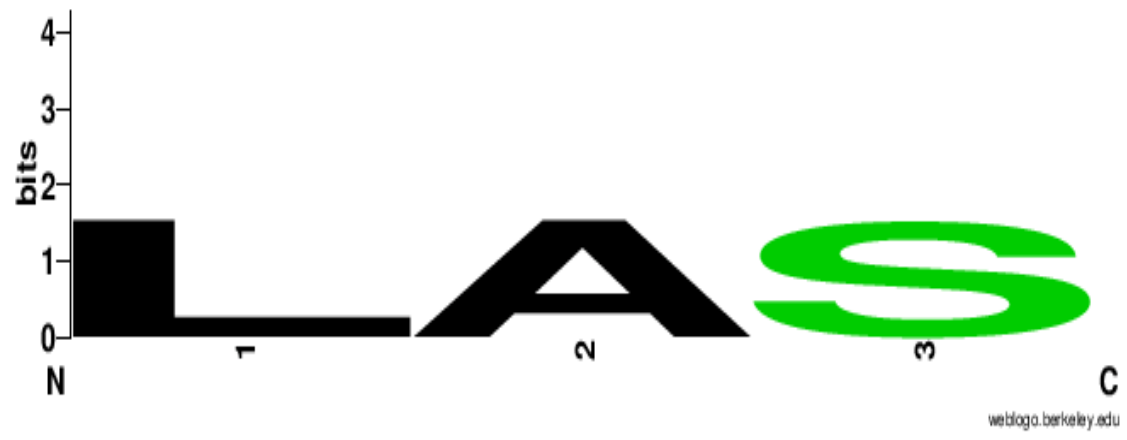

	Nr.	Sequence
CDR3	C07	QQNNEDPYT
	C09	QQNNEDPWT
	C12	QQNNEDPYT
	C19	QQNNEDPYT
	**C20**	**QQNNEDPYT**

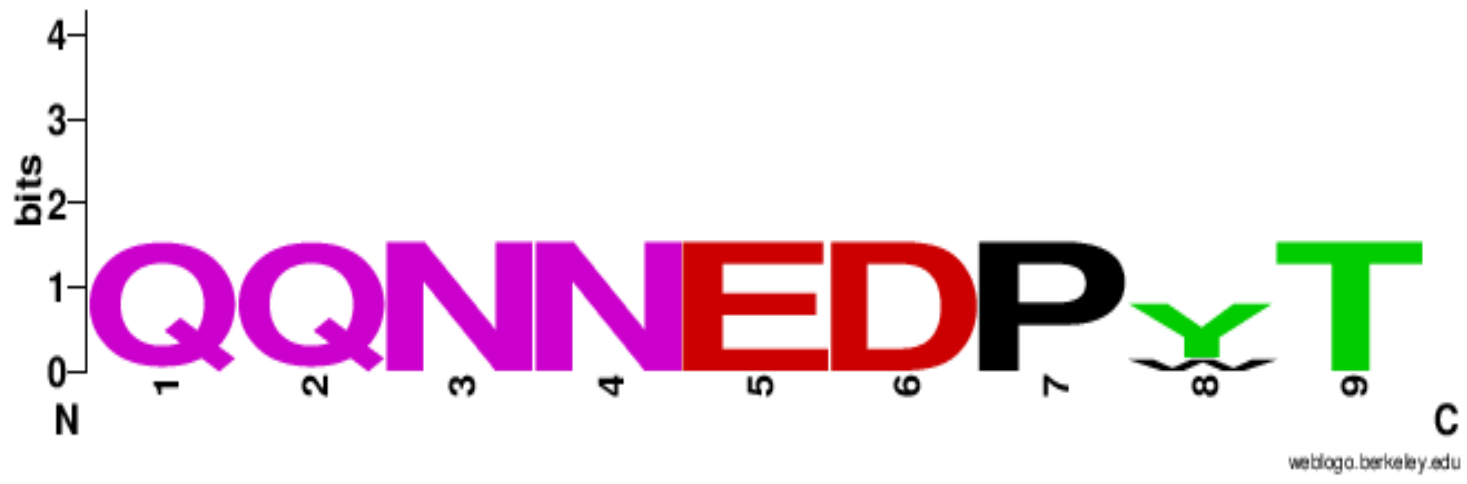

Supplementary Figure 1: The complementary determining regions (CDR) sequences of the five analysed anti-IL-6R mAbs have a high degree of sequence similarity.

The figure summarizes the sequences of all six CDRs for each of the analysed anti-IL-6R mAbs (left side). The CDRs of C20 mAb are marked in bold. The sequence logos have been generated using WebLogo (right sight).[1]

Supplementary Figure 2

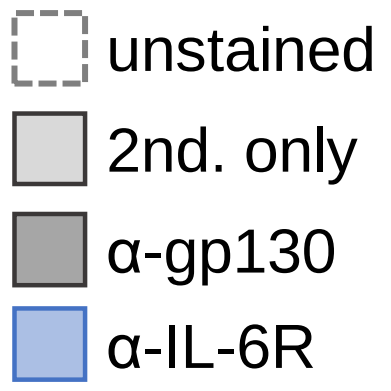

A Ba/F3_wt

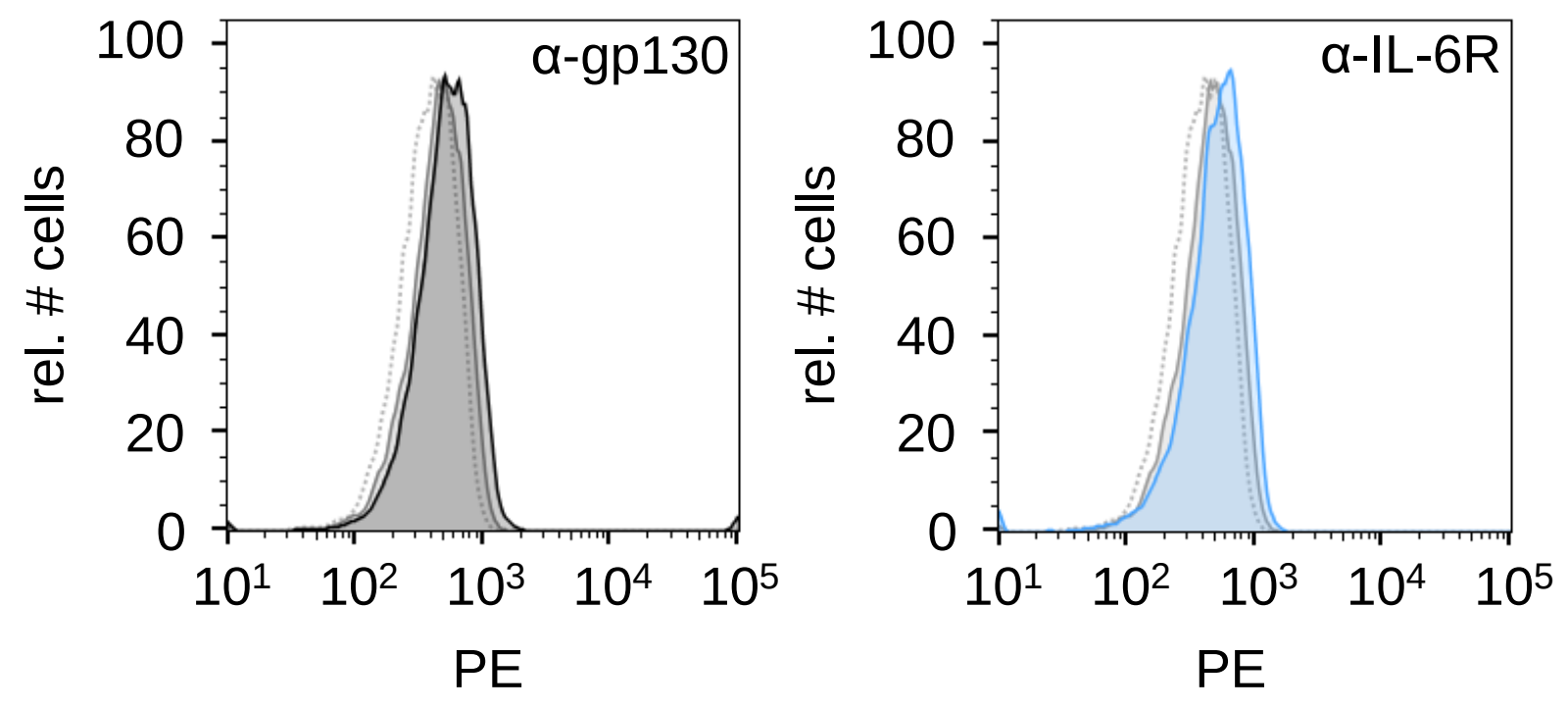

B Ba/F3_hugp130

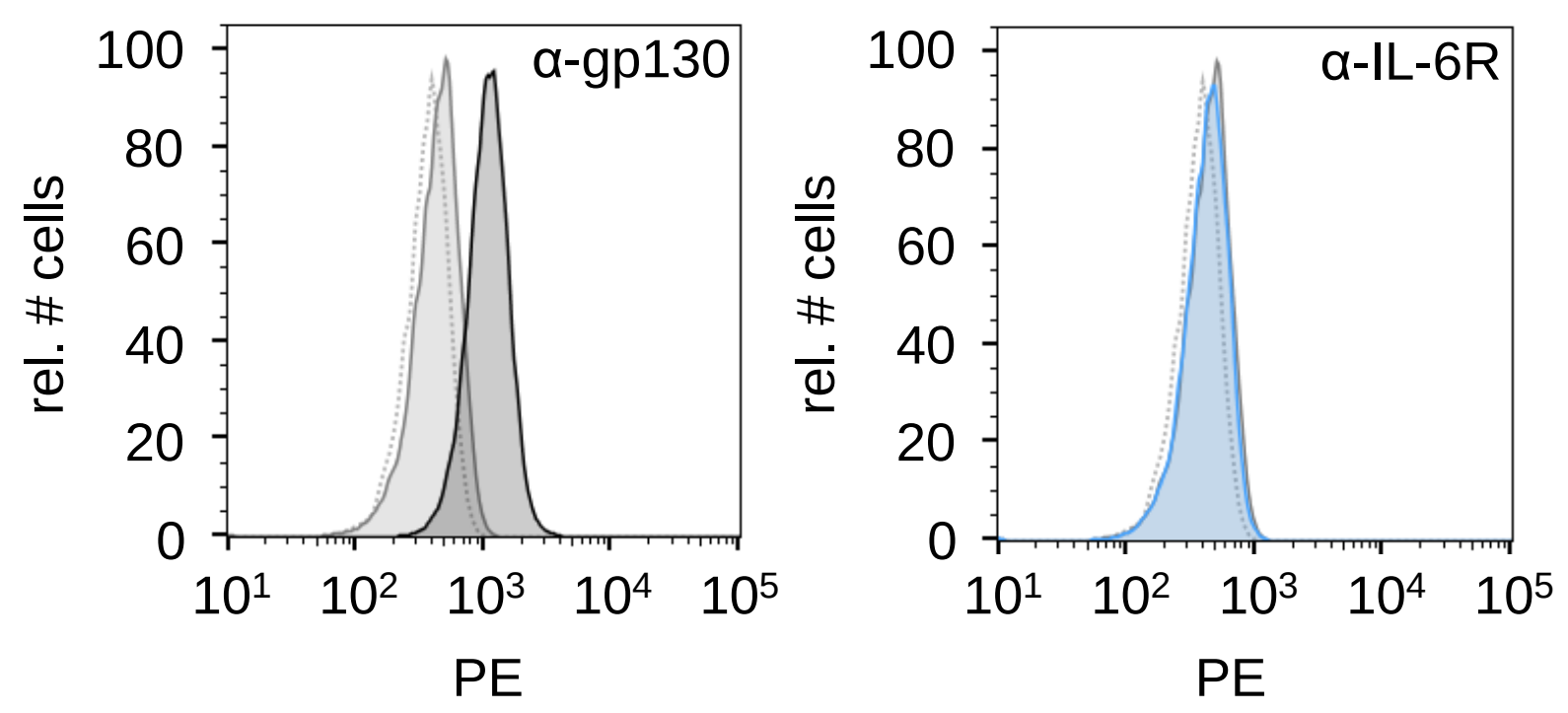

C Ba/F3_hugp130_huIL-6R

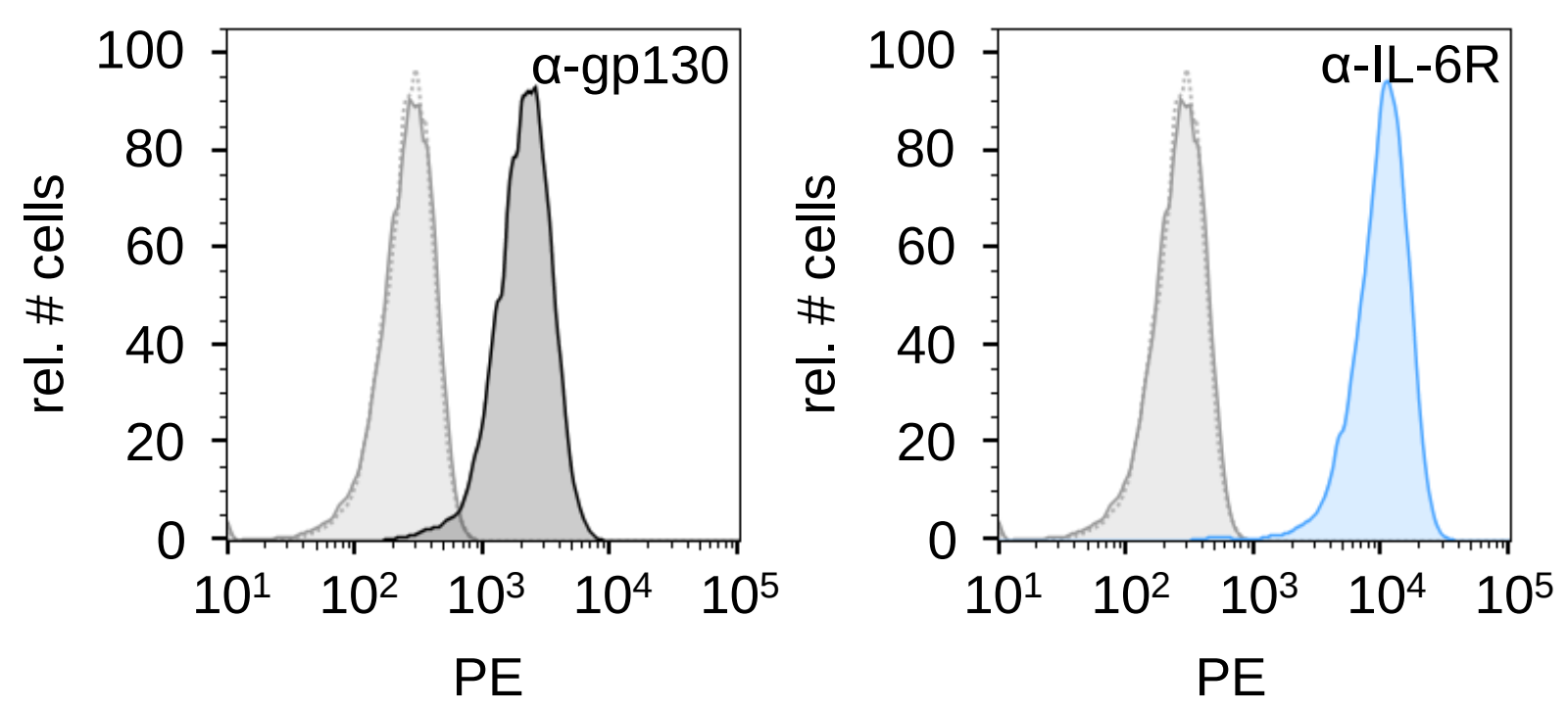

D HEK293

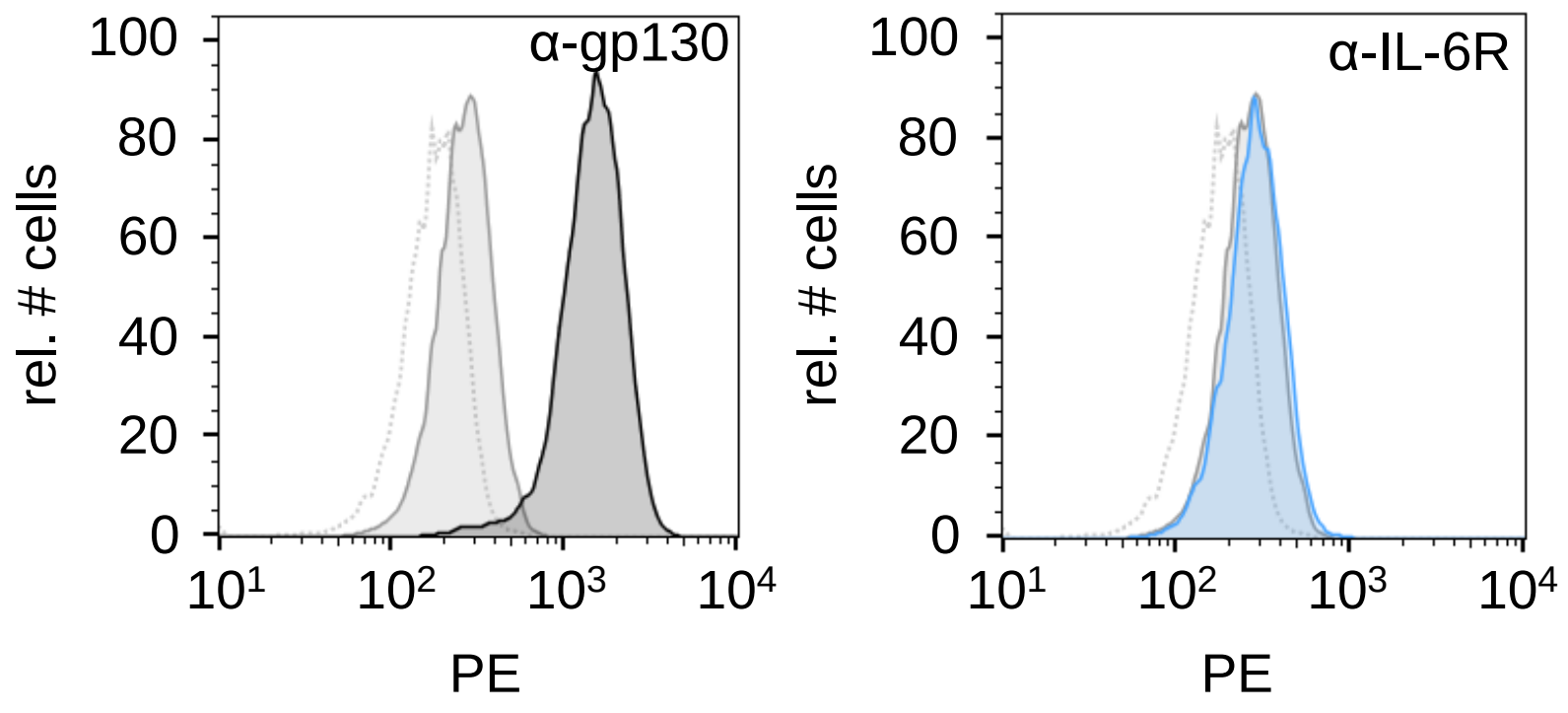

E HEK293_huIL-6R

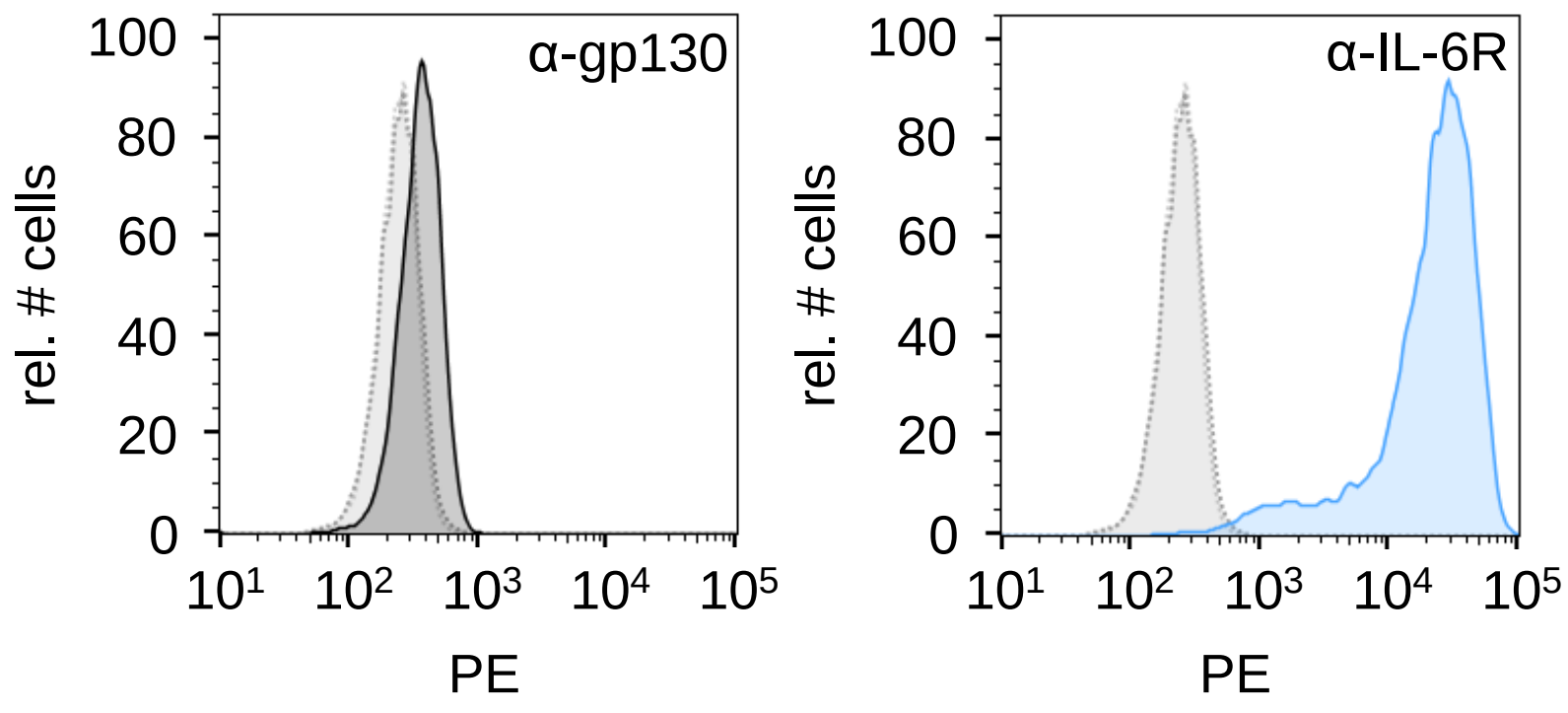

F HepG2

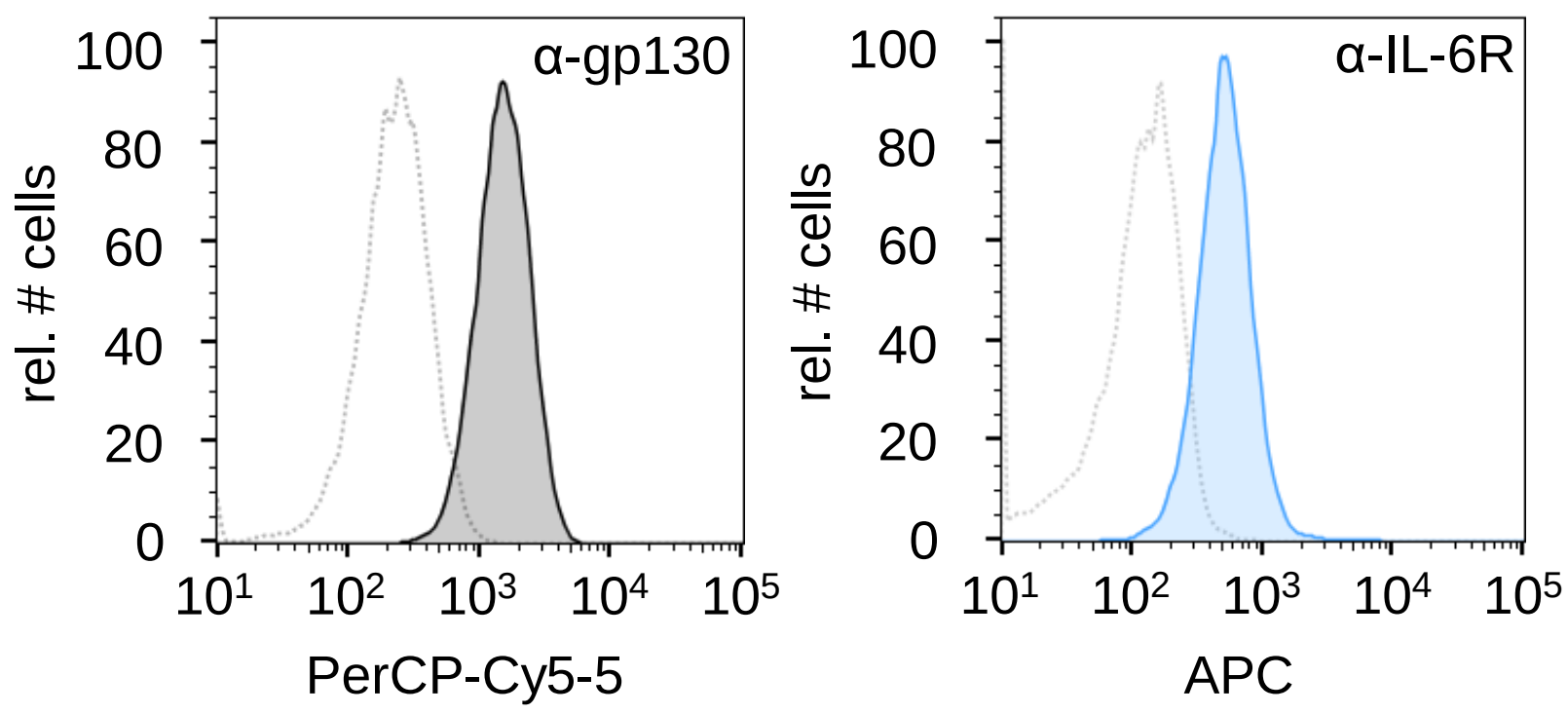

G HuARLT-2

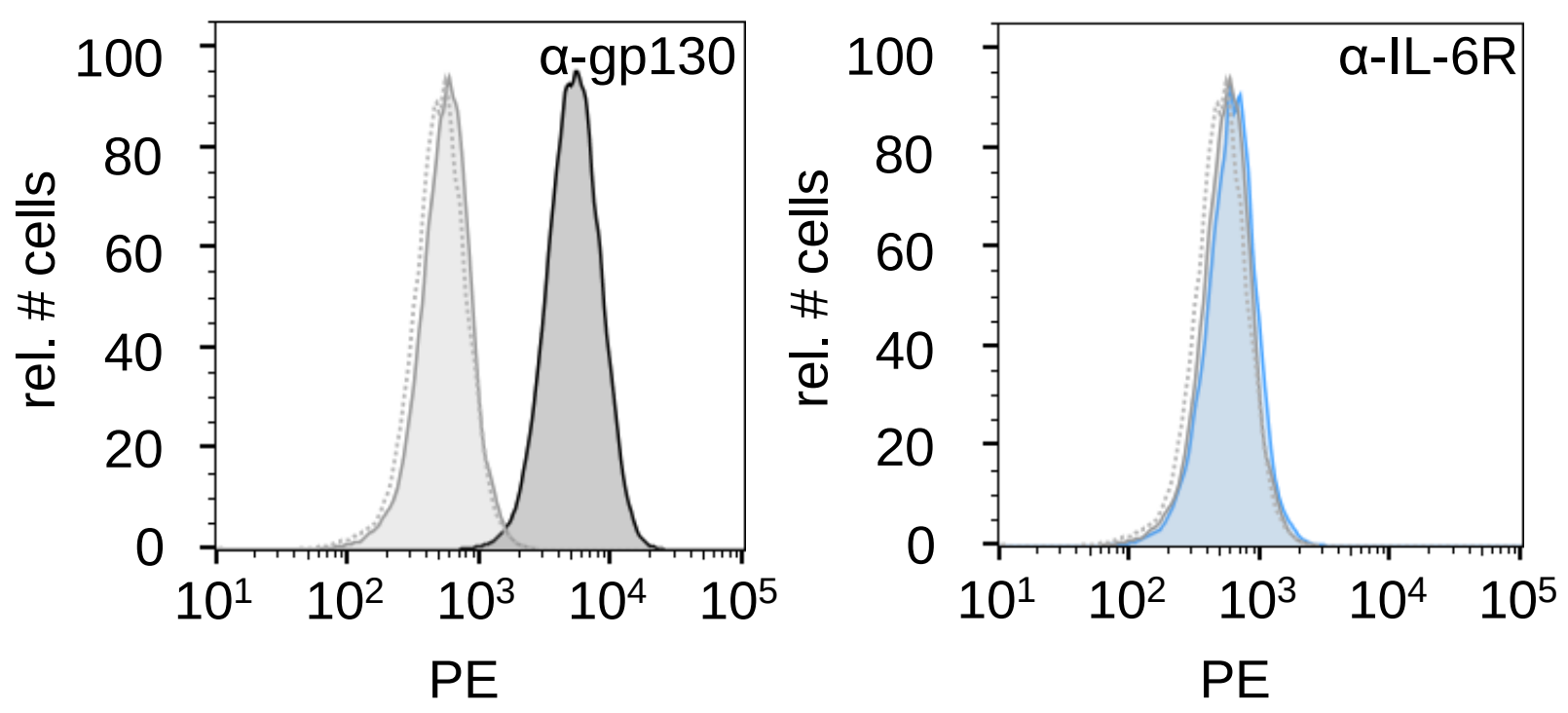

Supplementary Figure 2: Surface expression of gp130 and IL-6R differs between the analysed cell lines

Suspension cells **(A-C)** were taken directly out of the cell culture medium. Adherent cells **(D-G)** were detached from the cell culture dishes using Accutase (Biowest, #L0950-100). 300,000 cells per sample were transferred into 5 ml polystyrene tubes (Fisher scientific, #352052) and centrifuged (230 g, 4 °C, 5 min). The supernatant was removed from the cell pellet and the cells were stained:

- for **A-E** and **G**
  - Samples “Unstained” and “2nd only” were resuspended in 100 µl FACS buffer (EDTA (0.5 mM) + BSA (bovine serum albumin) (1 %) in PBS).
  - Sample “α-gp130” was resuspended in 100 µl anti-gp130 antibody (Active Bioscience GmbH, clone B-R3, #852.060.000, Lot RSP-PF220401D1) diluted 1:100 in FACS buffer.
  - Sample “α-IL-6R” was resuspended in anti-human IL-6R (Active Bioscience GmbH, clone B-R6, #852.030.000, Lot RSP-PF181122D3) diluted 1:100 in FACS buffer.
  - Cells were incubated on ice in the dark for 30 min and 3 ml FACS buffer was added to each sample. Cells were centrifuged (230 g, 4 °C, 5 min) and the supernatant was carefully removed.
  - Sample “Unstained” was resuspended in 100 µl FACS buffer.
  - Samples “2nd only”, “α-gp130” and “α-IL-6R” were each resuspended in R-Phycoerythrin-coupled secondary antibody (Jackson Immuno Research, #115-116-146) diluted 1:100 in FACS buffer.
  - Cells were incubated on ice in the dark for 30 min. 3 ml FACS buffer was added to each sample. Cells were centrifuged (230 g, 4 °C, 5 min) and the supernatant was carefully removed.
- for **F**
  - Sample “Unstained” was resuspended in 100 µl FACS buffer.
  - Sample “α-gp130” was resuspended in 100 µl anti-human gp130 antibody coupled to PE-CF594 (BD, catalogue # 564152, Lot 4121753) diluted 1:100 in FACS buffer.
  - Sample “α-IL-6R” was resuspended in 100 µl anti-human IL-6R antibody coupled to APC (BioLegend, catalogue # 352806, Lot B427671) diluted 3:100 in FACS buffer.
  - Cells were incubated on ice in the dark for 30 min. 3 ml FACS buffer was added to each sample. The cells were centrifuged (230 g, 4 °C, 5 min) and the supernatant was carefully removed.

After staining the cells were taken up in 200 µl FACS buffer and analysed by flow-cytometry. 10,000 individual cells were analysed per condition. Data were recorded on a BD FACS Canto II equipped with 3 lasers (405 nm, 488 nm, 663 nm) using BD FACS Diva Version 10.6.3 (BD). Data analyses was performed with FlowJo™ (BD, version: 10.6.1).

Supplementary Figure 3

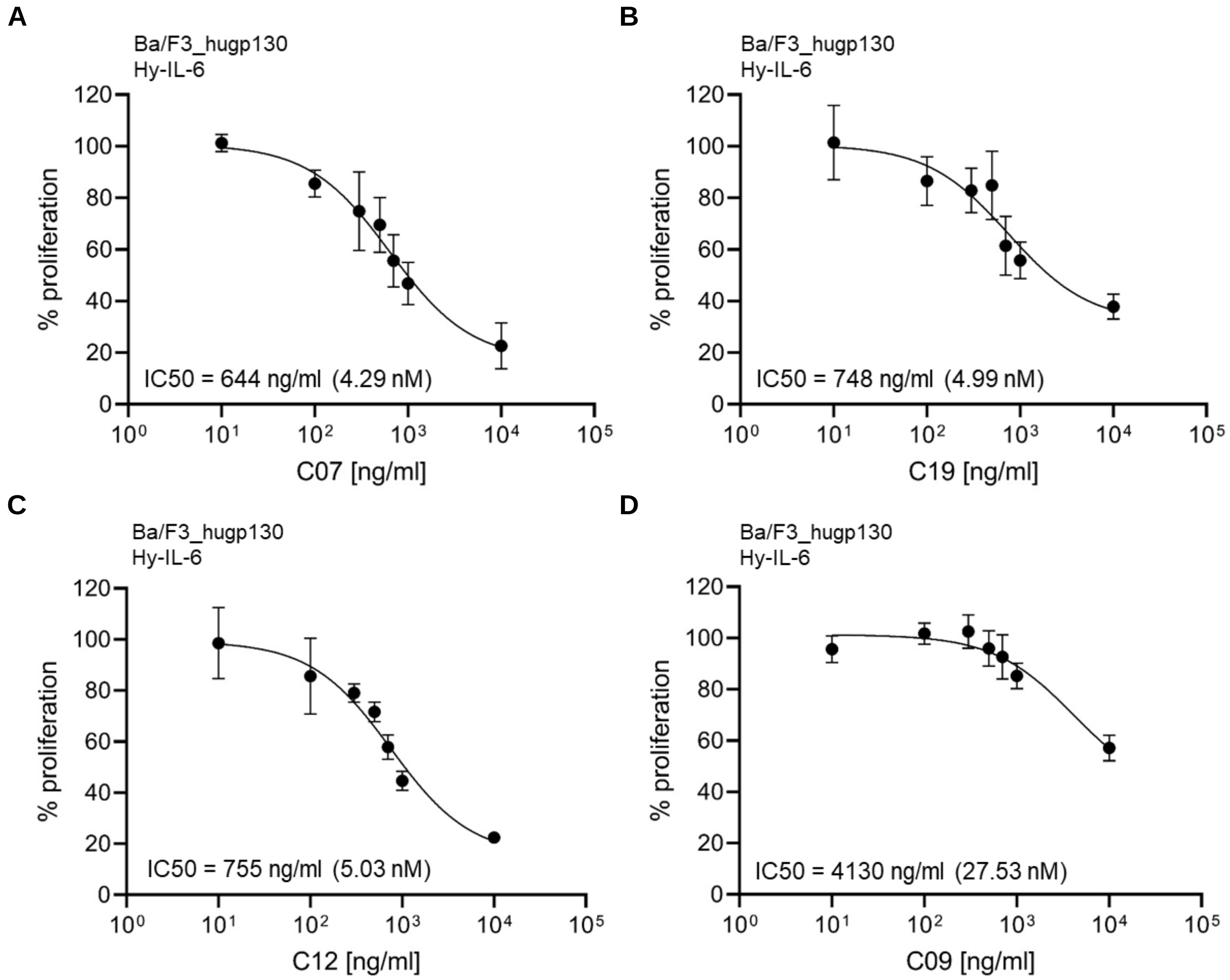

Supplementary Figure 3: All analysed anti-IL-6R mAbs dose-dependently inhibit Hy-IL-6-induced trans-signalling

5,000 Ba/F3_hugp130 cells were cultivated for two days with Hy-IL-6[2] (28.1 ng ml^{-1} equimolar to 10 ng ml^{-1} IL-6, Biotechne, #8954-SR) and increasing amounts of **(A)** C07 mAb, **(B)** C19 mAb, **(C)** C12 mAb and **(D)** C09 mAb. Viable cells were determined using Cell Titer Blue reagent (Promega, #G8080) after 48 h. The absorbance measured for cell suspensions grown with Hy-IL-6 in the absence of mAbs was set to 100 %. Mean and SD of n = 3 independent experiments are shown. Curve fitting for calculation of IC50 was done with four parameters least squares fit in GraphPad Prism (version 11.0.2, Dotmatics).

Supplementary Table 2: IC50s of the anti-IL-6R mAbs and Fab in IL-6 trans-signalling dependent proliferation assays

IC50s were determined as described in Fig. 3 and Supplementary Fig. 3.

<table>
<tr><th>Cell type</th><th>Stimulus</th><th>Antibody</th><th>IC50
ng ml^{-1}</th><th>IC50
nM</th></tr>
<tr><td rowspan="12">Ba/F3_hugp130</td><td rowspan="6">IL-6 (10 ng ml^{-1}) +
sIL-6R (200 ng ml^{-1})</td><td>C20 mAb</td><td>531.6</td><td>3.5</td></tr>
<tr><td>C20 Fab</td><td>158.5</td><td>3.2</td></tr>
<tr><td>C07 mAb</td><td>756.5</td><td>5.0</td></tr>
<tr><td>C09 mAb</td><td>5269.0</td><td>35.1</td></tr>
<tr><td>C12 mAb</td><td>863.0</td><td>5.8</td></tr>
<tr><td>C19 mAb</td><td>1214.0</td><td>8.1</td></tr>
<tr><td rowspan="6">Hy-IL-6 (28.1 ng ml^{-1},
equimolar to 10 ng ml^{-1} IL-6)</td><td>C20 mAb</td><td>304.2</td><td>2.0</td></tr>
<tr><td>C20 Fab</td><td>98.8</td><td>2.0</td></tr>
<tr><td>C07 mAb</td><td>644.2</td><td>4.3</td></tr>
<tr><td>C09 mAb</td><td>4130.0</td><td>27.5</td></tr>
<tr><td>C12 mAb</td><td>755.0</td><td>5.0</td></tr>
<tr><td>C19 mAb</td><td>748.3</td><td>5.0</td></tr>
</table>

Supplementary Note 2: Sequence alignment of murine and human IL-6R

A sequence alignment with the amino acid sequences of the human and murine IL-6R was performed using the BLASTp (protein-protein BLAST) algorithm from NCBI.[3] The alignment shows 53 % sequence identity between murine and human IL-6R. Important amino acids for BS-IIIb are marked.

Input sequences:

Human IL-6R (Swiss-Prot: P08887.1)

```
>sp|P08887.1|IL6RA_HUMAN RecName: Full=Interleukin-6 receptor subunit
alpha;
MLAVGCALLAALLAAPGAALAPRRCPAQEVARGVLTSLPGDSVTLTCPGVEPEDNATVHWVLRKPAAGSH
PSRWAGMGRRLLLRSVQLHDSGNYSCYRAGRPAGTVHLLVDVPPEEPQLSCFRKSPLSNVVCEWGPRSTP
SLTTKAVLLVRKFQNSPAEDFQEPCQYSQESQKFSCQLAVPEGDSSFYIVSMCVASSVGSKFSKTQTFQG
CGILQPDPPANITVTAVARNPRWLSVTWQDPHSWNSSFYRLRFELRYRAERSKTFTTWMVKDLQHHCVIH
DAWSGLRHVVQLRAQEEFGQGEWSEWSPEAMGTPWTESRSPPAENEVSTPMQALTTNKDDDNILFRDSAN
ATSLPVQDSSSVPLPTFLVAGGSLAFGTLLCIAIVLRFKKTWKLRALKEGKTSMHPPYSLGQLVPERPRP
TPVLVPLISPPVSPSSLGSDNTSSHNRPDARDPRSPYDISNTDYFFPR
```

Murine IL-6R (UniProtKB/Swiss-Prot: P22272.2):

```
>sp|P22272.2|IL6RA_MOUSE RecName: Full=Interleukin-6 receptor subunit
alpha;
MLTVGCTLLVALLAAPAVALVLGSCRALEVANGTVTSLPGATVTLICPGKEAAGNVTIHWVYSGSQNREW
TTTGNTLVLRDVQLSDTGDYLCSLNDHLVGTVPLLVDVPPEEPKLSCFRKNPLVNAICEWRPSSTPSPTT
KAVLFAKKINTTNGKSDFQVPCQYSQQLKSFSCQVEILEGDKVYHIVSLCVANSVGSKSSHNEAFHSLKM
VQPDPPANLVVSAIPGRPRWLKVSWQHPETWDPSYYLLQFQLRYRPVWSKEFTVLLLPVAQYQCVIHDAL
RGVKHVVQVRGKEELDLGQWSEWSPEVTGTPWIAEPRTTPAGILWNPTQVSVEDSANHEDQYESSTEATS
VLAPVQESSSMSL
```

Results
Query: sp|P08887.1|IL6RA_HUMAN RecName: Full=Interleukin-6 receptor subunit alpha;
Query ID: lcl|Query_3897803 Length: 468

Sbjct: sp|P22272.2|IL6RA_MOUSE RecName: Full=Interleukin-6 receptor subunit alpha
Sequence ID: Query_3897805 Length: 460
Range 1: 1 to 460

Score:451 bits(1159), Expect:1e-160,
Method: Compositional matrix adjust.,
Identities:253/474(53%), Positives:313/474(66%), Gaps:20/474(4%)

```
Query  1    MLAVGCALLAALLAAPGAALAPRRCPAQEVARGVLTSLPGDSVTLTCPGVEPEDNATVHW 60
            ML VGC LL ALLAAP  AL    C A EVA G +TSLPG +VTL CPG E   N T+HW
Sbjct  1    MLTVGCTLLVALLAAPAVALVLGSCRALEVANGTVTSLPGATVTLICPGKEAAGNVTIHW 60

Query  61   VLRKPAAGSHPSRWAGMGRRLLLRSVQLHDSGNYSCYRAGRPAGTVHLLVDVPPEEPQLS 120
            V     +GS    W   G  L+LR VQL D+G+Y C       GTV LLVDVPPEEP+LS
Sbjct  61   VY----SGSQNREWTTTGNTLVLRDVQLSDTGDYLCSLNDHLVGTVPLLVDVPPEEPKLS 116
```

```
Query  121 CFRKSPLSNVVCEWGPRSTPSLTTKAVLLVRKFQNSPAE-DFQEPCQYSQESQKFSCQLA 179
           CFRK+PL N +CEW P STPS TTKAVL  +K   +  + DFQ PCQYSQ+ + FSCQ+
Sbjct  117 CFRKNPLVNAICEWRPSSTPSPTTKAVLFAKKINTTNGKSDFQVPCQYSQQLKSFSCQVE 176

Query  180 VPEGDSSFYIVSMCVASSVGSKFSKTQTFQGCGILQPDPPANITVTAVARNPRWLSVTWQ 239
           + EGD  ++IVS+CVA+SVGSK S  + F    ++QPDPPAN+ V+A+   PRWL V+WQ
Sbjct  177 ILEGDKVYHIVSLCVANSVGSKSSHNEAFHSLKMVQPDPPANLVVSAIPGRPRWLKVSWQ 236

Query  240 DPHSWNSSFYRLRFELRYRAERSKTFTTWMVKDLQHHCVIHDAWSGLRHVVQLRAQEEFG 299
            P +W+ S+Y L+F+LRYR   SK FT  ++    Q+ CVIHDA  G++HVVQ+R +EE
Sbjct  237 HPETWDPSYYLLQFQLRYRPVWSKEFTVLLLPVAQYQCVIHDALRGVKHVVQVRGKEELD 296

Query  300 QGEWSEWSPEAMGTPWT-ESRSPPAENEVSTPMQALTTNKDDDNILFRDSANATSL--PV 356
            G+WSEWSPE  GTPW  E R+ PA   +  P Q    +  +    +  S  ATS+  PV
Sbjct  297 LGQWSEWSPEVTGTPWIAEPRTTPA-GILWNPTQVSVEDSANHEDQYESSTEATSVLAPV 355

Query  357 QDSSSVPLPTFLVAGGSLAFGTLLCIAIVLRFKKTWKLRALKEGKTS--MHPPYSLGQLV 414
           Q+SSS+ LPTFLVAGGSLAFG LLC+ I+LR K+ WK  A KE KT+    PPYSLG L
Sbjct  356 QESSSMSLPTFLVAGGSLAFGLLLCVFIILRLKQKWKSEAEKESKTTSPPPPPYSLGPL- 414

Query  415 PERPRPTPVLVPLISPPVSPSSLGSDNTSSHNRPDARDPRSPYDISNTDYFFPR 468
               +PT +LVPL+    +P S GSDNT +H+    RD +SPYD SN DY FPR
Sbjct  415 ----KPTFLLVPLL----TPHSSGSDNTVNHSCLGVRDAQSPYDNSNRDYLFPR 460
```

Supplementary Note 3: Linear epitope mapping of C20

The epitope of the anti-IL-6R mAb C20 was attempted to be analysed by a linear epitope mapping approach performed by PEPperPRINT GmbH. The sequence of IL-6R (accession number: P08887) without signal peptide was used. As C20 binds to sIL-6R, analyses were restricted to the sequence of the sIL-6R (yellow):

MLAVGCALLAALLAAPGAALAPRRCPAQEVARGVLTSLPGDSVTLTCPGVEPEDNATVHWVLRKPAAGSH
PSRWAGMGRRLLLRSVQLHDSGNYSCYRAGRPAGTVHLLVDVPPEEPQLSCFRKSPLSNVVCEWGPRSTP
SLTTKAVLLVRKFQNSPAEDFQEPCQYSQESQKFSCQLAVPEGDSSFYIVSMCVASSVGSKFSKTQTFQG
CGILQPDPPANITVTAVARNPRWLSVTWQDPHSWNSSFYRLRFELRYRAERSKTFTTWMVKDLQHHCVIH
DAWSGLRHVVQLRAQEEFGQGEWSEWSPEAMGTPWTESRSPPAENEVSTPMQALTTNKDDDNILFRDSAN
ATSLPVQDSSSVPLPTFLVAGGSLAFGTLLCIAIVLRFKKTWKLRALKEGKTSMHPPYSLGQLVPERPRP
TPVLVPLISPPVSPSSLGSDNTSSHNRPDARDPRSPYDISNTDYFFPR

The sequence of sIL-6R was elongated with neutral GSGSGSG linkers at the C- and N-termini to avoid truncated peptides. The elongated antigen sequence was converted into linear 15 amino acid peptides with a peptide-peptide overlap of 14 amino acids. The resulting IL-6R peptide microarrays contained 339 different peptides printed in duplicate (678 spots) and were framed by additional HA (YPYDVPDYAG, 80 spots) control peptides. The IL-6R peptide microarray was pre-stained with the secondary and control antibodies in incubation buffer to investigate background interactions with the antigen-derived peptides that could interfere with the assays. Simultaneous incubation of all further IL-6R peptide microarrays with human IgG1 antibody C20 at concentrations of 1 µg ml^{-1}, 10 µg $ml^{-1}$and 100 µg ml^{-1} was followed by staining with the secondary antibody and read-out with an Innopsys InnoScan 710-IR Microarray Scanner at scanning gains of 50/10 (red/green). The additional HA peptides framing the peptide microarrays were simultaneously stained with the control antibody as internal quality control to confirm the assay performance and the peptide microarray integrity. Quantification of spot intensities and peptide annotation were based on the 16-bit grey scale tiff files. Microarray image analysis was done with PepSlide® Analyzer. An intensity map was

generated and interactions in the peptide map highlighted by an intensity colour code with red for high and white for low spot intensities. Pre-staining of an IL-6R peptide microarray with the secondary and control antibodies showed a few very weak background interactions of the secondary antibody with some basic IL-6R peptides of the microarray (Supplementary Figure 4). These presumably non-specific ionic background interactions were quantified and considered for the analysis of the assays.

Supplementary Figure 4

A) Background Signal

Goat anti-human IgG (H+L) DyLight680

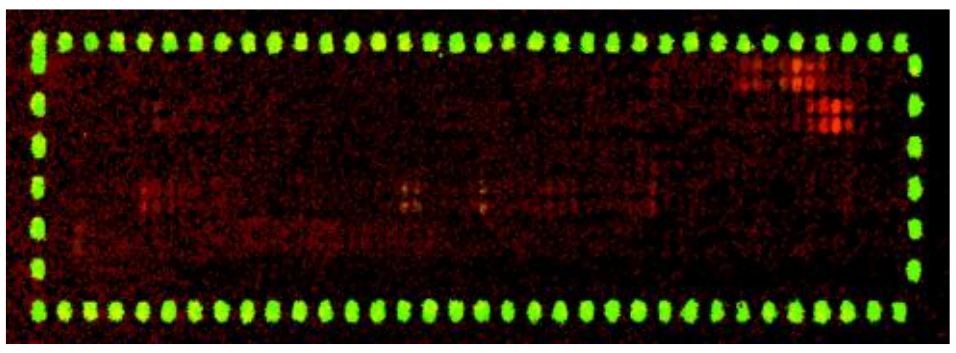

B) Anti-human IL-6R + secondary antibody

Human IgG C20, 100 µg/ml

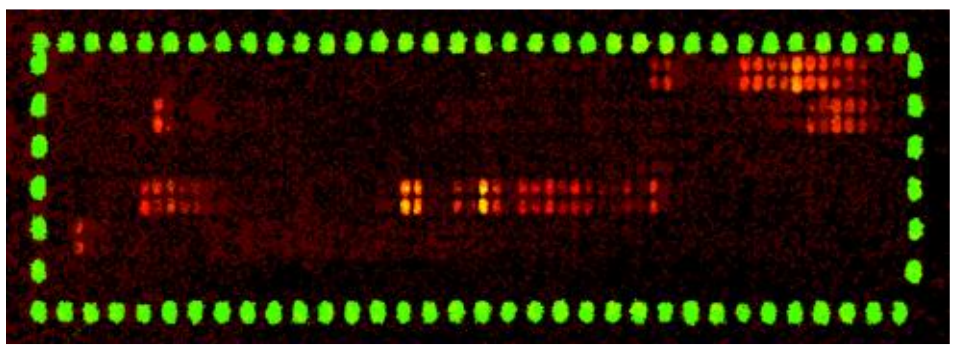

Supplementary Figure 4: Weak interaction of C20 with linear peptides derived from human IL-6R

A) The IL-6R peptide microarray was pre-stained with the secondary antibodies to investigate background interactions with the antigen-derived peptides. **B)** The IL-6R peptide microarray was stained with C20 mAb (100 µg ml^{-1}) followed by staining with secondary antibody and read-out with an Innopsys InnoScan 710-IR Microarray Scanner at scanning gains of 50/10 (red/green). HA peptides framing the peptide microarrays were simultaneously stained with the control antibody as internal quality control. Incubation of further IL-6R peptide microarrays with human IgG1 antibody C20 resulted in a number of weak interactions with epitope-like spot patterns formed by adjacent peptides with the common motifs RWAGMGRR, SLTTKAVLLVR, ITVTAVARNPR, QDPHSWNSSFYRLR, NSSFYRLRFELR and RFELRYRAER as well as with single peptides QLHDSGNYSCYRAGR and YRAERSKTFTTWMVK at low to moderate signal-to-noise ratio. Almost all interactions were also observed with the secondary antibody alone and are thus regarded as background binding. The interactions with peptides with the common motifs RWAGMGRR, QDPHSWNSSFYRLR and NSSFYRLRFELR exhibited significantly higher intensity levels compared to the background binding of the secondary antibody, what may indicate sample binding to the corresponding peptides. However, the corresponding peptides exhibited all a basic character and possibly also resulted from non-specific ionic antibody binding. Therefore, these interactions have to be considered with caution. The overall weak and unclear interaction profile of human IgG1 antibody C20 might result from a conformational contribution to antibody binding, which could hardly or not be mimicked by the linear IL-6R peptides of the microarray.

Supplementary Figure 5

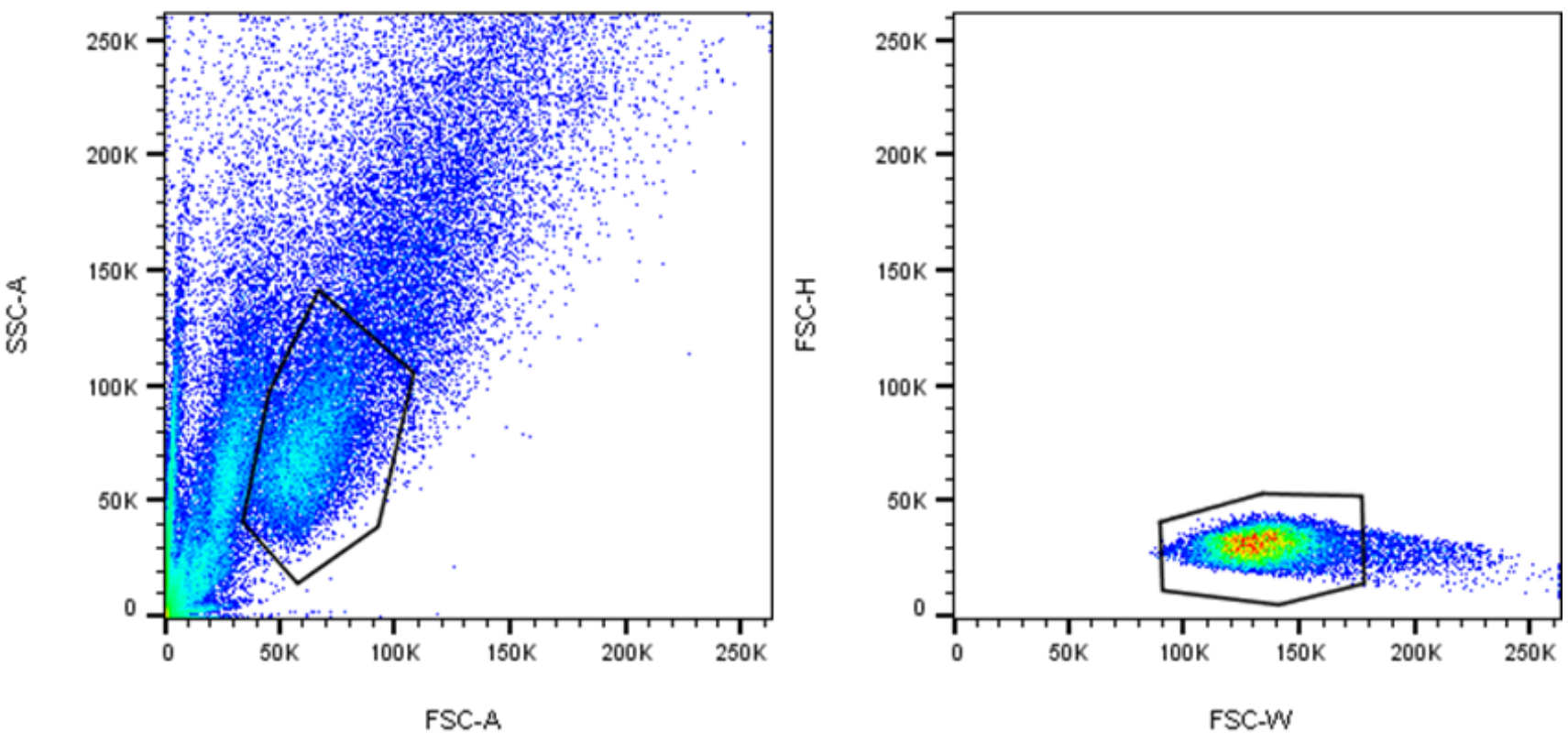

Supplementary Figure 5: Strategy to gate on single cells

Living cells were gated from a SSC-A against FSC-A dot plot. From this population single cells were gated by plotting FSC-H against FSC-W and gating on cells with low FSC-W. Only living, single cells were subsequently analysed. Representative dot plots are shown.

Supplementary Figure 6

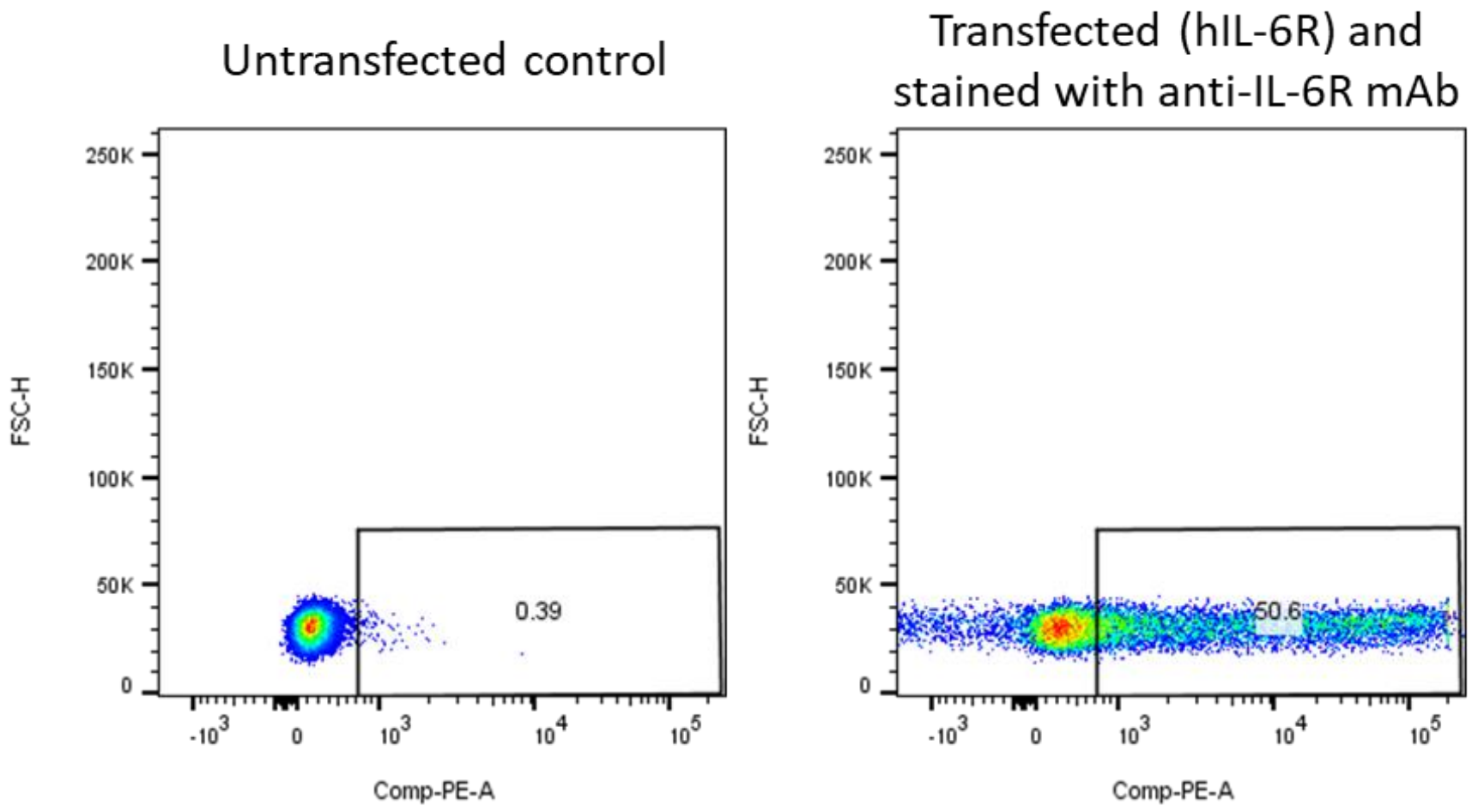

Supplementary Figure 6: Strategy to gate on cells expressing chimeric receptors
HEK293T cells were transiently transfected with expression vectors coding for chimeric human-murine IL-6R[4] (Fig. 7A) using lipofectamine2000 (Thermofisher Scientific, #11668027). 24 h later cells were detached with Accutase (Biowest, # 0950-100) and stained with anti-IL-6R C20 mAb (1:100 in 1 % BSA-EDTA buffer: 1 % BSA, 0.5 mM EDTA in PBS) and phycoerythrin (PE) coupled anti-human IgG Fc (Biolegend, #410708, 1:100 in 1 % BSA-EDTA buffer). As a transfection control, the cells were co-transfected with a GFP expression vector (Supplementary Fig. 7). PE and GFP fluorescence intensities were analysed by flow cytometry using a sequential gating strategy. First, live cells were identified based on side scatter area (SSC-A) and forward scatter area (FSC-A) parameters. From this population, single cells were gated using a forward scatter height (FSC-H) versus forward scatter width (FSC-W) plot to exclude doublets (Supplementary Fig. 5). PE-positive transfected cells were subsequently identified, with the threshold for PE positivity defined relative to the autofluorescence of untransfected control cells. Representative dot plots shown.

Supplementary Figure 7

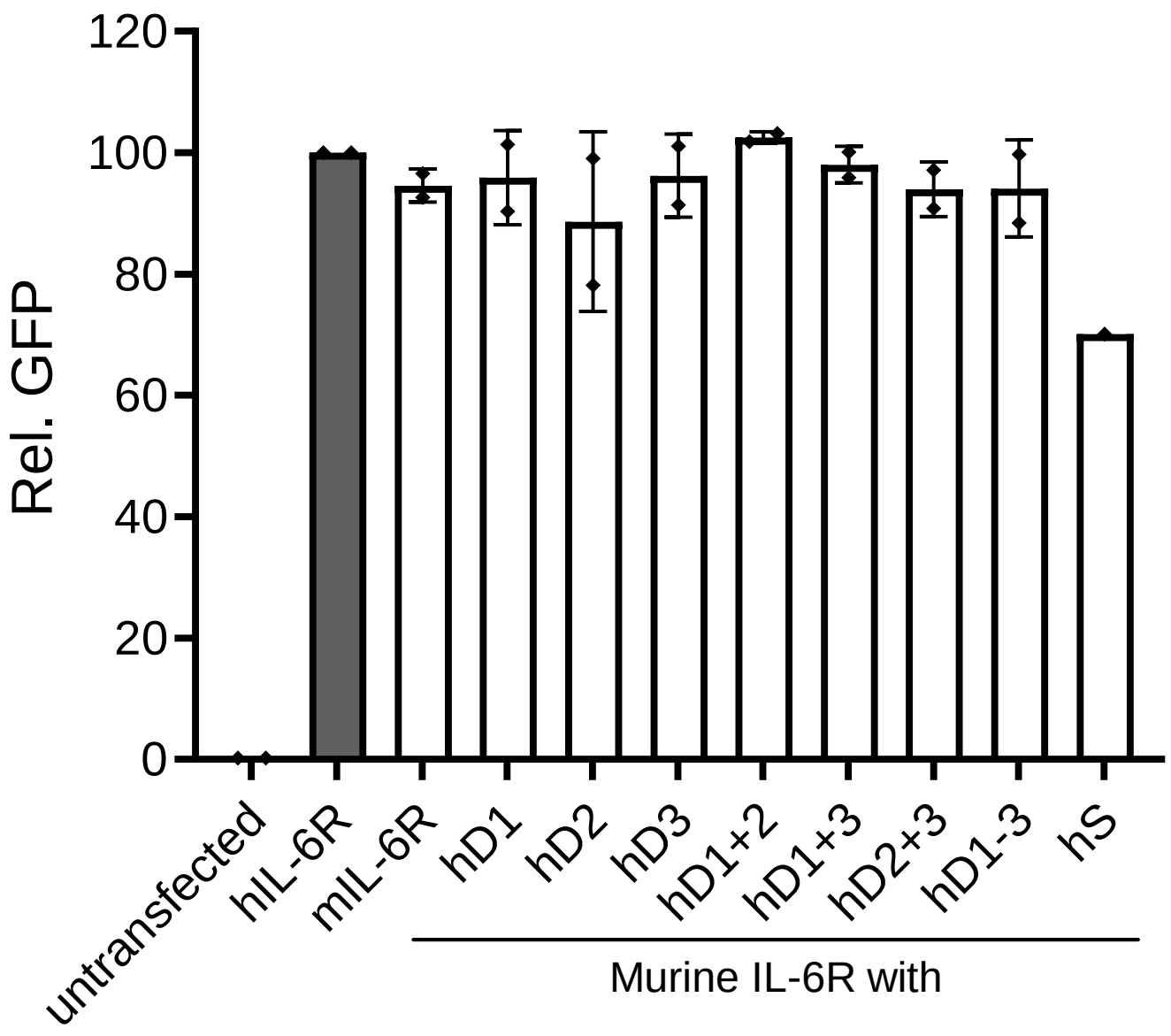

Supplementary Figure 7: Transfected cells express comparable levels of GFP

HEK293T cells were transiently transfected with the indicated expression vectors coding for chimeric human-murine IL-6R[4] (Fig. 7A) using lipofectamine2000 (Thermofisher Scientific, #11668027). 24 h later cells were detached with Accutase (Biowest, # 0950-100) and stained with anti-IL-6R C20 mAb (1:100 in 1 % BSA-EDTA buffer: 1 % BSA, 0.5 mM EDTA in PBS) and phycoerythrin (PE) coupled anti-human IgG Fc (Biolegend, #410708, 1:100 in 1 % BSA-EDTA buffer). As a transfection control, the cells were co-transfected with a GFP expression vector. PE and GFP fluorescence intensities were analysed by flow cytometry using a sequential gating strategy. First, live cells were identified based on side scatter area (SSC-A) and forward scatter area (FSC-A) parameters. From this population, single cells were gated using a forward scatter height (FSC-H) versus forward scatter width (FSC-W) plot to exclude doublets (Supplementary Fig. 5). The expression level of GFP is comparable in cells co-transfected with different chimeric IL-6R variants indicating that the different receptor variants are expressed equally well.

Cells were analysed on a BD FACS Canto II equipped with 3 lasers (405 nm, 488 nm, 663 nm) using BD FACS Diva Version 10.6.3 (BD). Data analyses was performed with FlowJo™ (BD, version: 10.6.1). Results of n= 2 independent experiments are shown.

Supplementary Figure 8

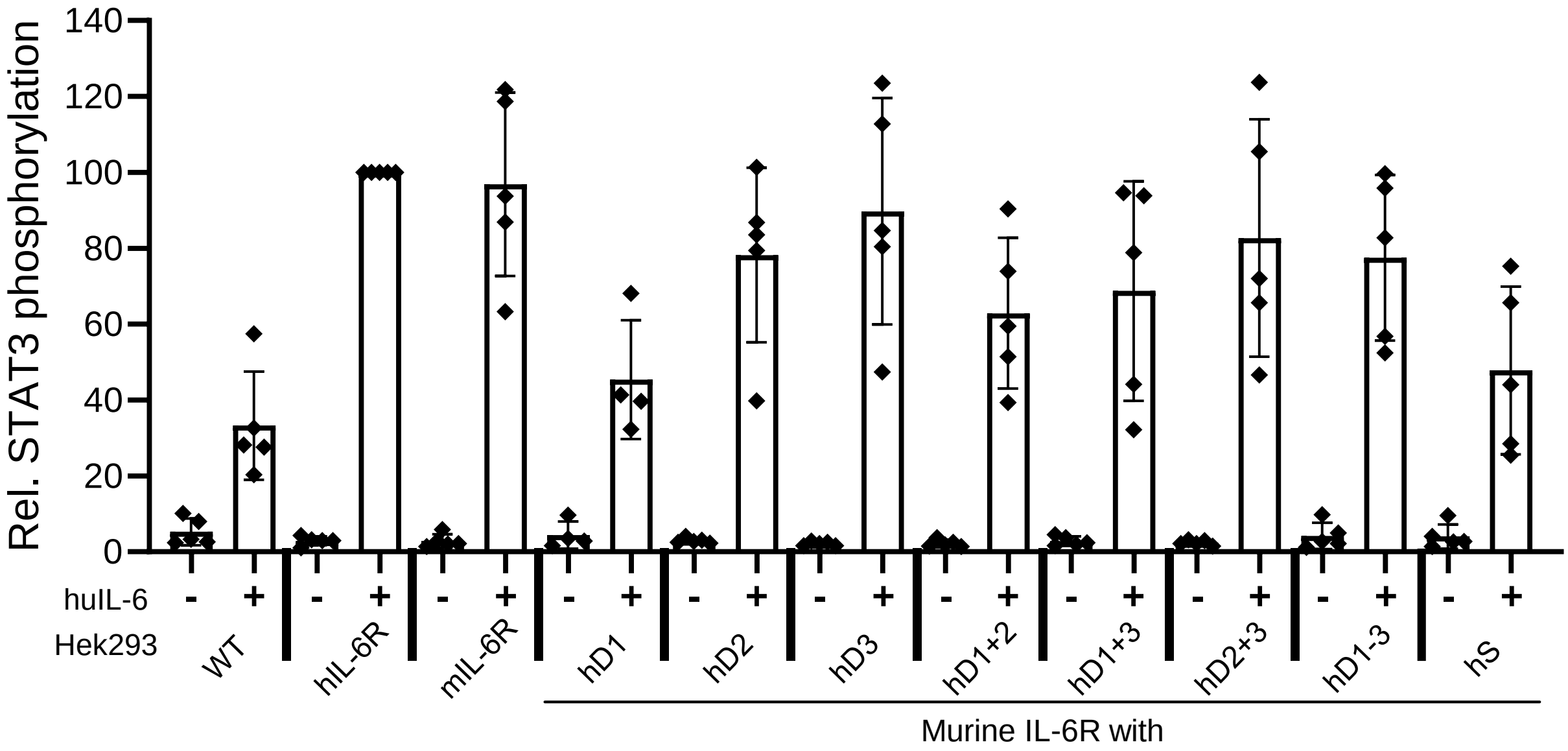

Supplementary Figure 8: Cells expressing chimeric murine/human IL-6R receptor variants are responsive to IL-6 stimulation

HEK293T cells were transiently transfected with the indicated expression vectors coding for chimeric human-murine IL-6R[4] (Fig. 7A) using lipofectamine2000 (Thermofisher Scientific, #11668027). Cells were starved for 2 h in serum free DMEM and then stimulated with human IL 6 (10 ng ml^{-1}) for 30 min. Subsequently, cells were detached with Accutase (Biowest, # 0950-100). STAT3 phosphorylation was evaluated by intracellular flow-cytometry using specific fluorophore-coupled antibodies against (p)Y705 STAT3. Live cells were identified based on side scatter area (SSC-A) and forward scatter area (FSC-A) parameters. From this population, single cells were gated using a forward scatter height (FSC-H) versus forward scatter width (FSC-W) plot to exclude doublets (Supplementary Fig. 5). STAT3 phosphorylation is comparable in cells transfected with different chimeric IL-6 receptor variants indicating that the different receptor variants are expressed equally well.

Cells were analysed on a BD FACS Canto II equipped with 3 lasers (405 nm, 488 nm, 663 nm) using BD FACS Diva Version 10.6.3 (BD). Data analyses was performed with FlowJo™ (BD, version: 10.6.1). Results of n= 2 independent experiments are shown.

.

Supplementary Figure 9

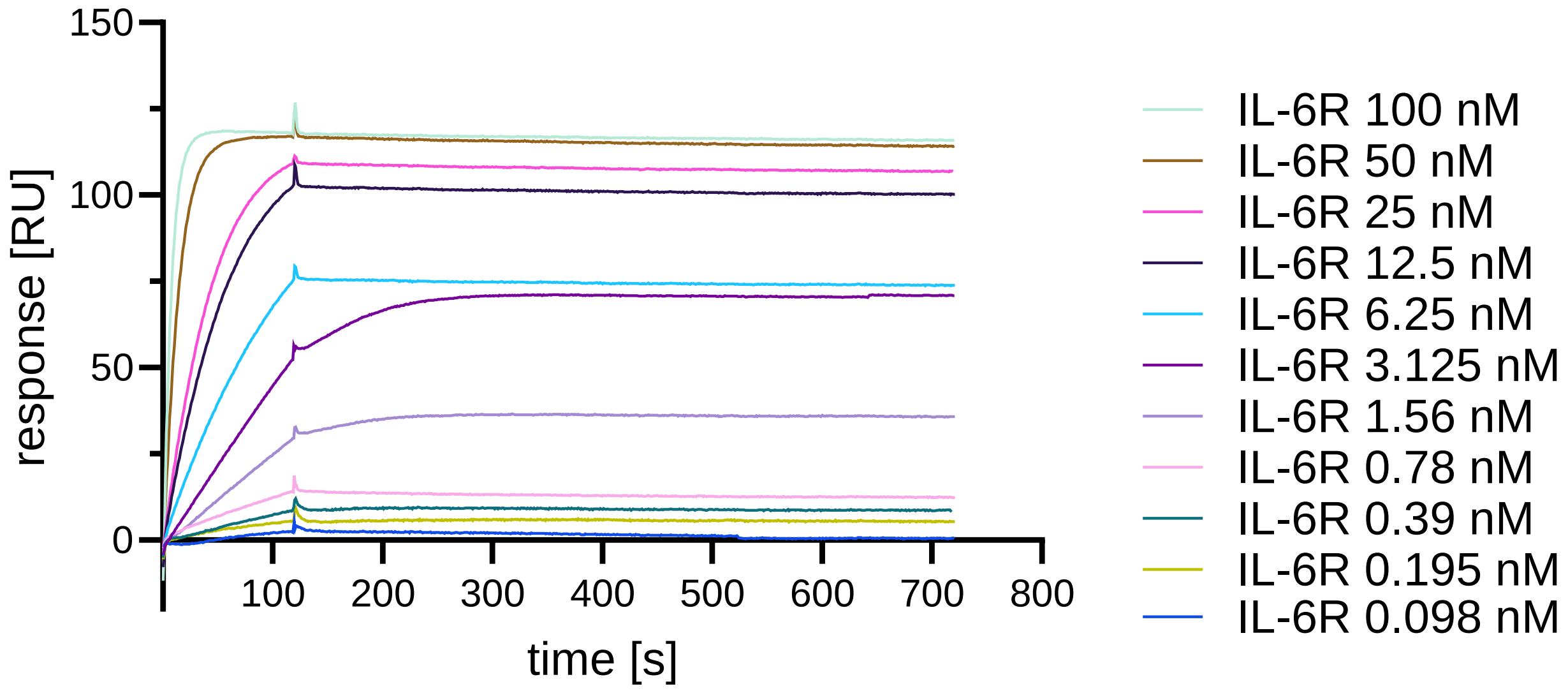

Supplementary Figure 9: Binding of C20 mAb to sIL-6R

SPR experiments were carried out at 25 °C using a BiacoreX 100 (Cytiva) in PBS containing 0.05 % surfactant P20 (Cytiva, #BR100054). Anti-IL-6R mAb C20 was immobilized onto a protein A sensor chip (Cytiva, #29127557, Lot #10349531) at a level of ~220 response units (RUs). Recombinant husIL-6R (R&D Systems, #227-SR-025/CF) was injected and flowed over the chip surface for 2 min at a flow rate of 30 µl min^{-1}. After each binding the sensor chip surface was washed and regenerated with 10 mM glycine-HCL (pH 2.1) for 2x 1 min at a flow rate of 30 µl min^{-1}. The data were analysed with Biacore X100 Control Software Version 2.1.0.201 (Cytiva). The graphs were plotted using GraphPad Prism (version 11.0.2, Dotmatics). The determined K_D values are presented in Supplementary Table 3.

Supplementary Figure 10

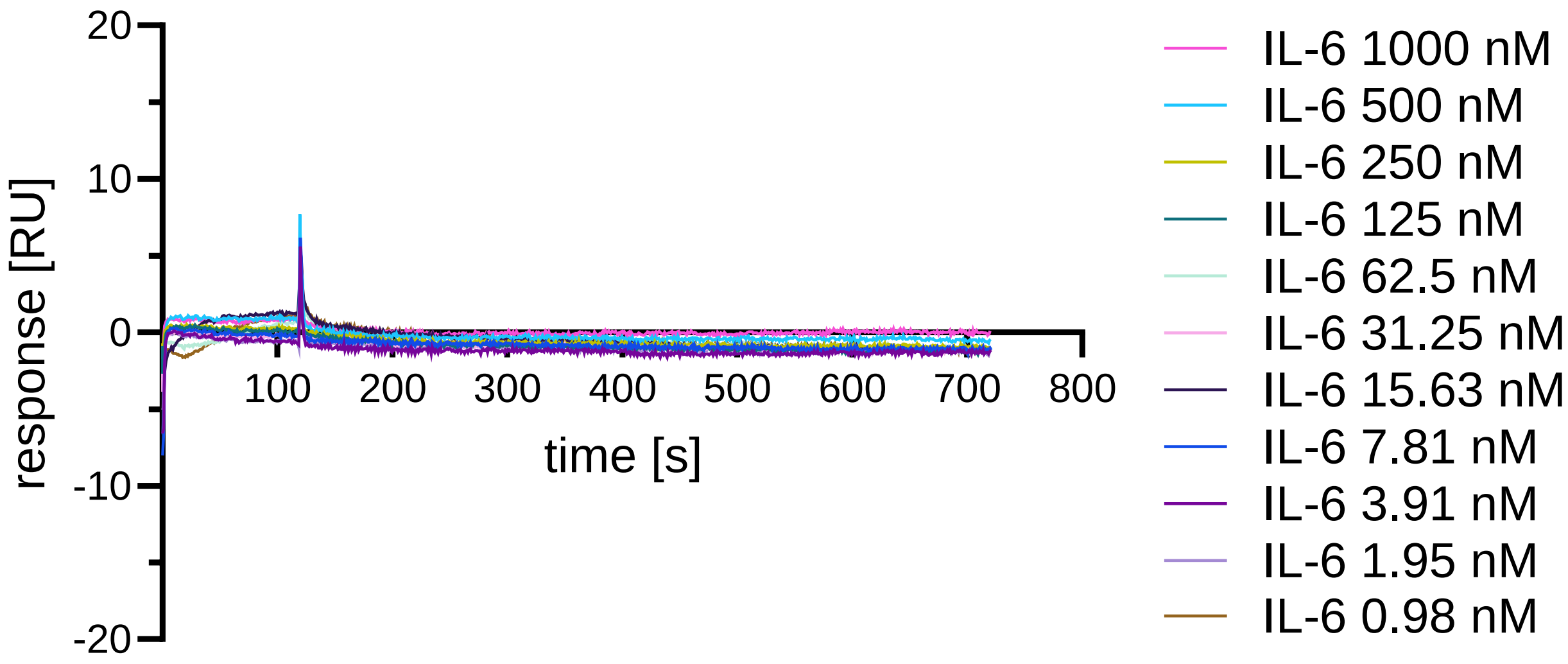

Supplementary Figure 10: C20 mAb does not bind to IL-6

SPR experiments were carried out at 25 °C using a BiacoreX 100 (Cytiva) in PBS containing 0.05 % surfactant P20 (Cytiva, #BR100054). Anti-IL-6R mAb C20 was immobilized onto a protein A sensor chip (Cytiva, #29127557, Lot #10349531) at a level of ~220 response units (RUs). Recombinant huIL-6[5] was injected and flowed over the chip surface for 2 min at a flow rate of 30 µl min^{-1}. After each binding the sensor chip surface was washed and regenerated with 10 mM glycine-HCL (pH 2.1) for 2x 1 min at a flow rate of 30 µl min^{-1}. The data were analysed with Biacore X100 Control Software Version 2.1.0.201 (Cytiva). The graphs were plotted using GraphPad Prism (version 11.0.2, Dotmatics). The determined K_D values are presented in Supplementary Table 3.

Supplementary Figure 11

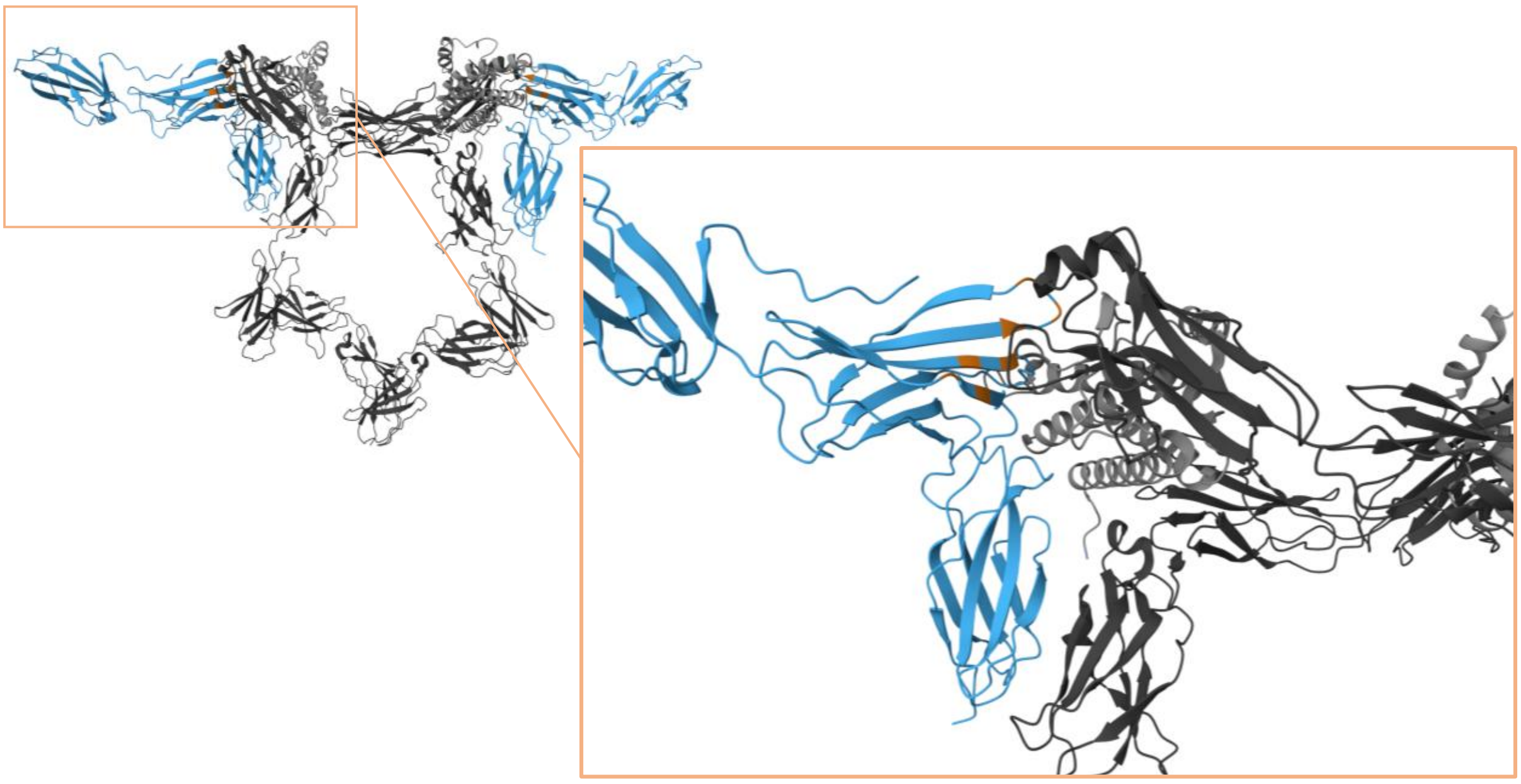

Supplementary Figure 11: 3D crystal structure of a hexameric IL-6 receptor

The presented structure is based on PDB ID: 8D82.[6]

Colour code: IL-6 (grey), gp130 (black), sIL-6R (blue), important amino acids for BS-IIIb (orange).

Supplementary Figure 12

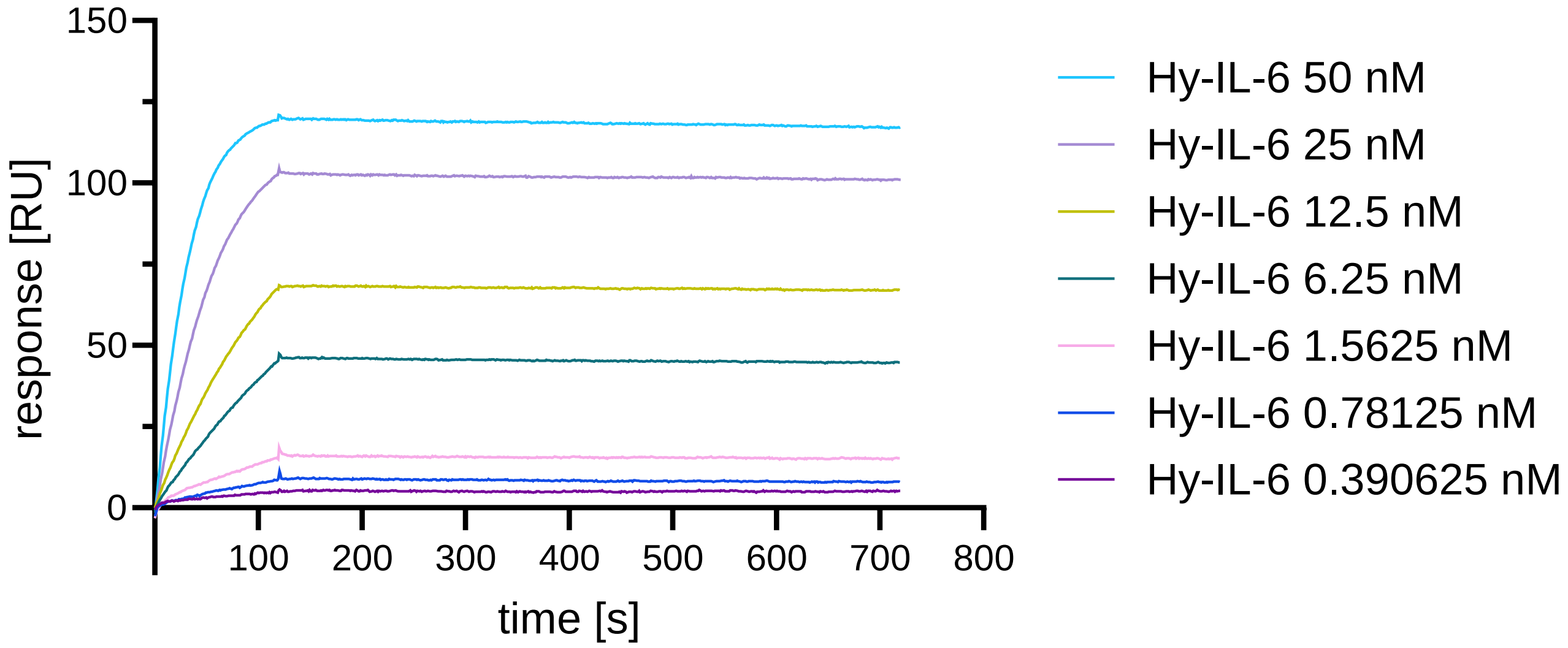

Supplementary Figure 12: Binding of C20 mAb to HyIL-6

SPR experiments were carried out at 25 °C using a BiacoreX 100 (Cytiva) in PBS containing 0.05 % surfactant P20 (Cytiva, #BR100054). Anti-IL-6R mAb C20 was immobilized onto a protein A sensor chip (Cytiva, #29127557, Lot #10349531) at a level of ~220 response units (RUs). Hy-IL-6[5] was injected and flowed over the chip surface for 2 min at a flow rate of 30 µl min^{-1}. After each binding the sensor chip surface was washed and regenerated with 10 mM glycine-HCL (pH 2.1) for 2x 1 min at a flow rate of 30 µl min^{-1}. The data were analysed with Biacore X100 Control Software Version 2.1.0.201 (Cytiva). The graphs were plotted using GraphPad Prism 9 (version 11.0.2, Dotmatics). The determined K_D values are presented in Supplementary Table 3.

Supplementary Figure 13

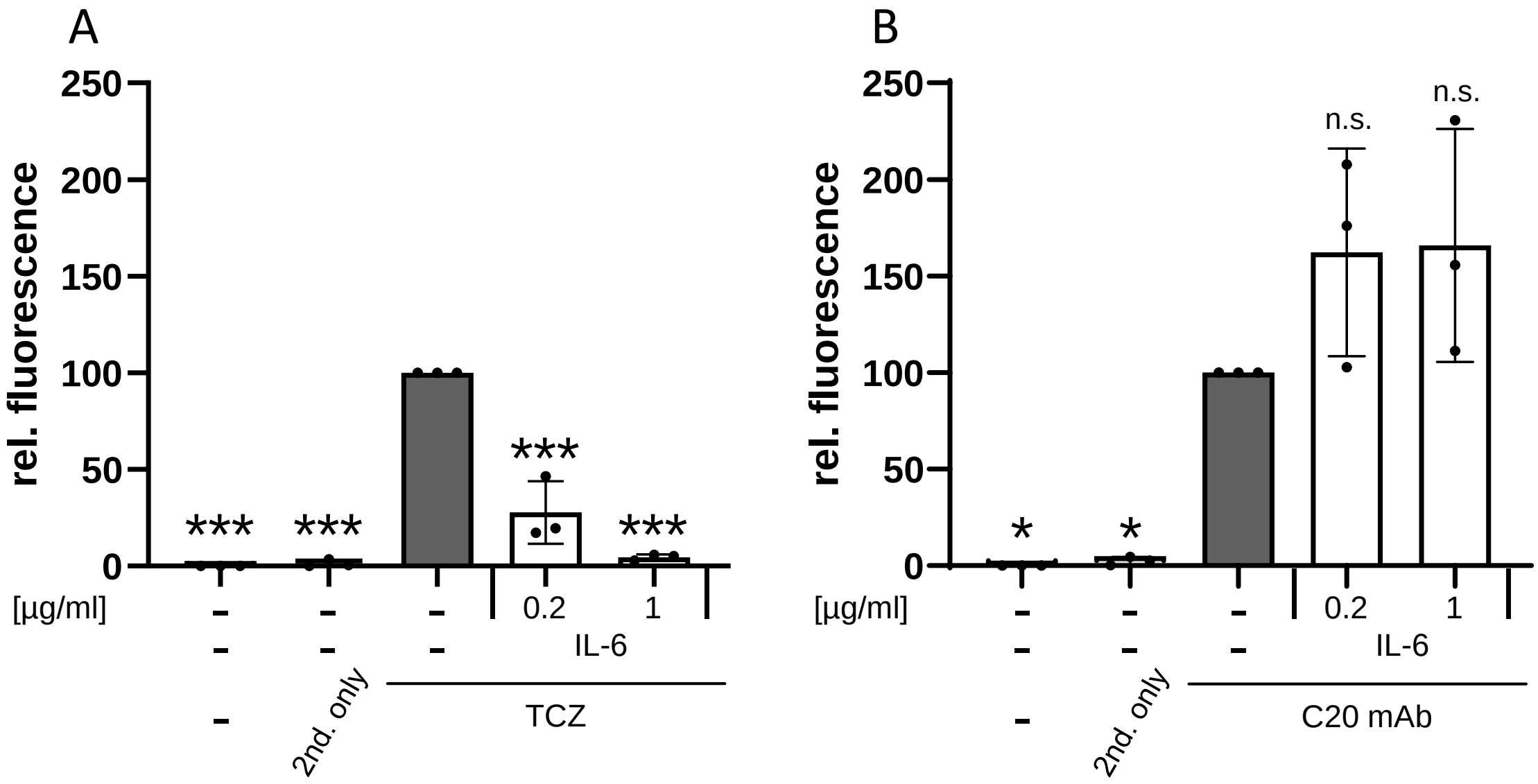

Supplementary Figure 13: Binding of Tocilizumab is reduced in the presence of huIL-6, while binding of C20 mAb is not reduced in the presence of huIL-6

200,000 Ba/F3_hugp130_huIL-6R cells were transferred into 5 ml polystyrene tubes (Fisher Scientific, #352052). The cells were centrifuged (230 g, 4 °C, 5 min) and then incubated at 4 °C for 30 min in 100 µl antibody solution:

(A) TCZ, 200 nM, 30 µg ml^{-1} (Thermo Fisher Scientific, #MA5-48061, Lot# ZA4166981)

(B) C20 mAb, 0.98 nM, 146 ng ml^{-1}

or in 1 % BSA-EDTA buffer in the absence or presence of huIL-6 (9.8 nM, 204 ng ml^{-1}or 48.8 nM, 1020 ng ml^{-1}).

As a control cells were left unstained. Cells were washed with 3 ml 1 % BSA-EDTA buffer and centrifuged (230 g, 4 °C, 5 min). Cells were incubated at 4°C for 30 min in the dark in antibody solution containing PE coupled anti-human IgG Fc (BioLegend, #410708, 1:100 in 1 % BSA-EDTA buffer) or in 1 % BSA-EDTA buffer. Cells were washed with 3 ml 1 % BSA-EDTA buffer before flow-cytometric analysis. Data was recorded on a BD FACS Canto II equipped with 3 lasers (405 nm, 488 nm, 663 nm) using BD FACS Diva Version 10.6.3 (BD). Data Analyses was performed with FlowJo™ (BD, version: 10.6.1). Mean and SD of n = 3 independent experiments are shown. One-way ANOVA with multiple comparisons: n.s. = non-significant, *p < 0.05, ***p < 0.001. P-values indicate differences to cells incubated without IL-6.

Supplementary Figure 14

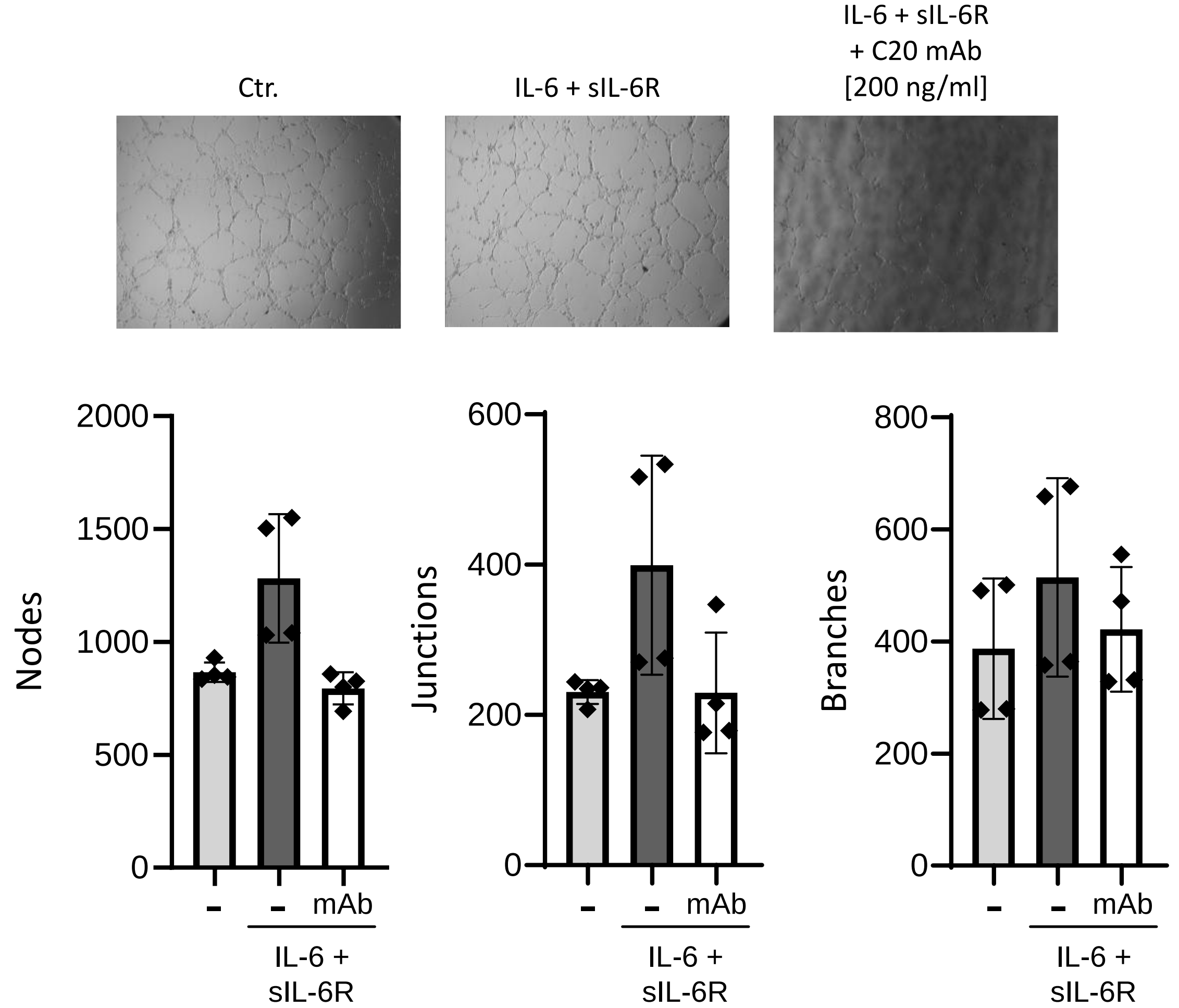

Supplementary Figure 14: C20 mAb reduces IL-6 trans-signalling-induced angiogenesis
HuARLT-2 cells were seeded for 48 h in endothelial growth medium with supplements and 2 µg ml^{-1} doxycycline and then stimulated for 24 h with huIL-6 (50 ng ml^{-1}) and husIL-6R (500 ng ml^{-1}) as indicated in the figures. µ-Slide 15 well angiogenesis slides (Ibidi, #81506) were coated with Matrigel (Corning, #354230). Matrigel was thawed slowly on ice before using, dispensed into wells (10 µl per well), and allowed to polymerize for 30 min at 37 °C. After stimulation, cells were detached with Trypsin, counted and seeded at a density of 7,800 cells per well in 50 µl of their respective conditioned supernatants onto µ-slide 15 well pre-coated with Matrigel. Cells were incubated at 37 °C for 6 h in a water saturated atmosphere containing 5 % CO_2, after which three representative images were taken per well using an AMG EVOS microscope (Thermo Fisher Scientific). Representative raw data from n=4 independent experiments are shown. Tube formation as represented by number of segments (Fig. 8D), nodes, junctions, and branches were analysed using Image J (version 1.54).[7]

Supplementary Figure 15

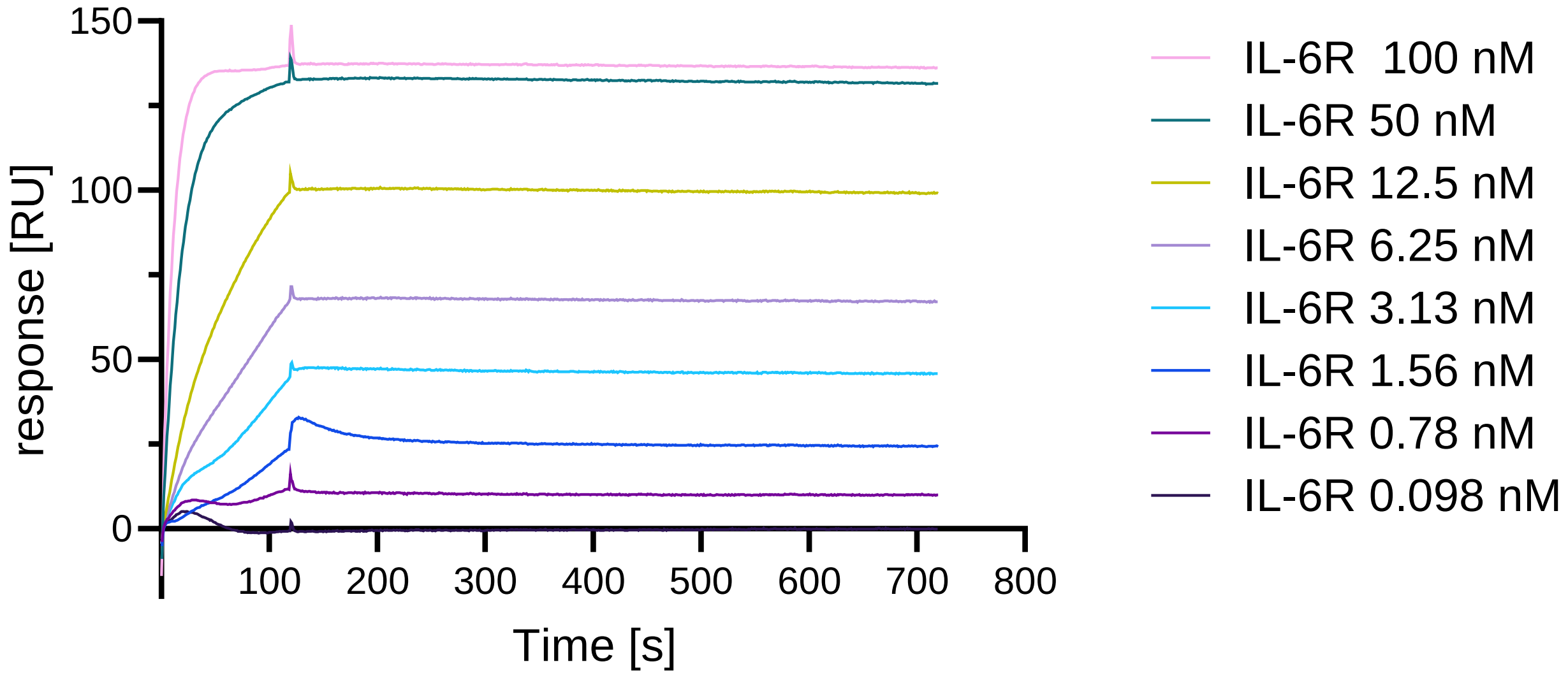

Supplementary Figure 15: Binding of C20 Fab to sIL-6R

SPR experiments were carried out at 25 °C using a BiacoreX 100 (Cytiva) in PBS containing 0.05 % surfactant P20 (Cytiva, #BR100054). Immobilization of anti-IL-6R Fab C20 onto a CM5 sensor chip (Cytiva, #BR100399, Lot #10295121) was performed according to the manufacturer's instructions of the Human Fab Capture Kit (Cytiva, #28958325) at a level of ~205 RUs. Recombinant husIL-6R (R&D Systems, #227-SR-025/CF) was injected and flowed over the chip surface for 2 min at a flow rate of 30 µl min^{-1}. After each binding the sensor chip surface was washed and regenerated with 10 mM glycine-HCL (pH 2.1) for 2x 1 min at a flow rate of 30 µl min^{-1}. The data were analysed with Biacore X100 Control Software Version 2.1.0.201 (Cytiva). The graphs were plotted using GraphPad Prism 9 (version 11.0.2, Dotmatics). The determined K_D values are presented in Supplementary Table 3.

Supplementary Figure 16

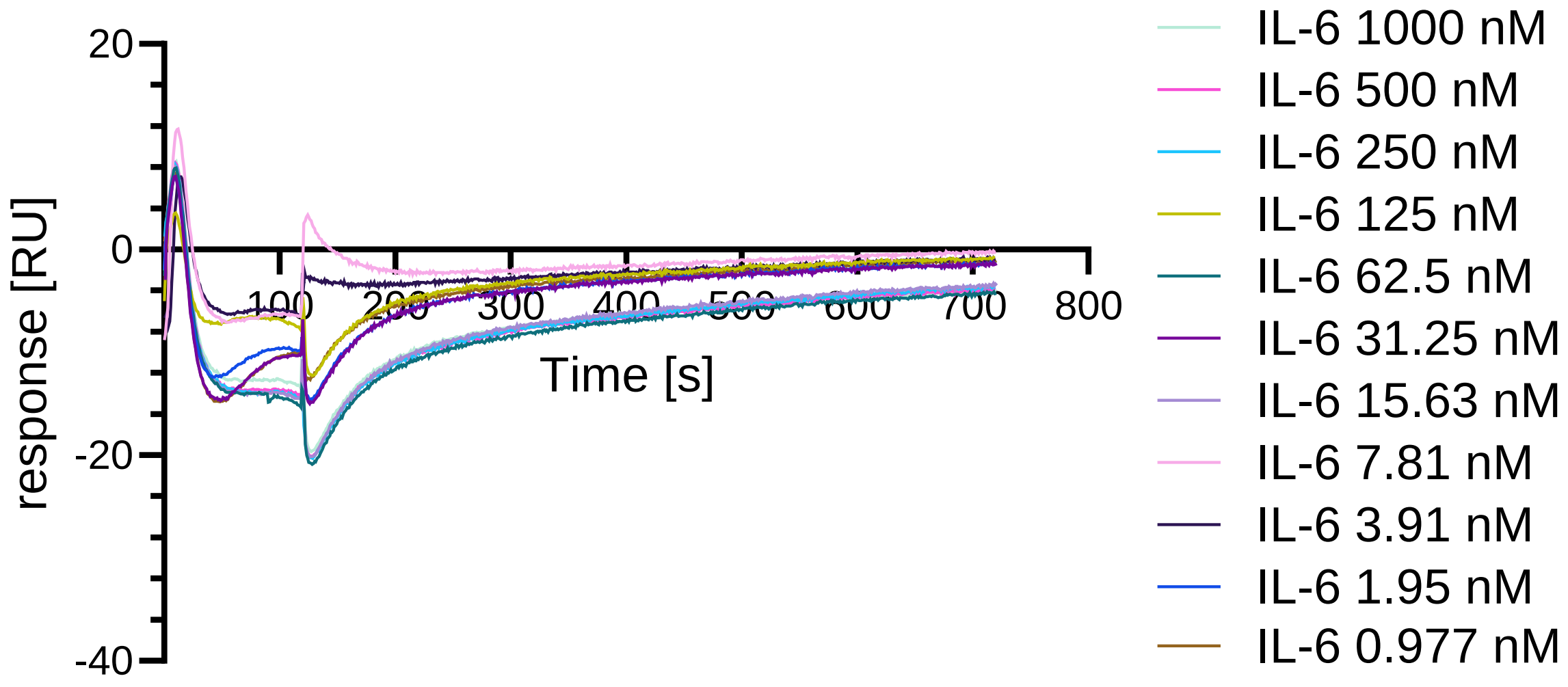

Supplementary Figure 16: C20 Fab does not bind to IL-6

SPR experiments were carried out at 25 °C using a BiacoreX 100 (Cytiva) in PBS containing 0.05 % surfactant P20 (Cytiva, #BR100054). Immobilization of anti-IL-6R Fab C20 onto a CM5 sensor chip (Cytiva, #BR100399, Lot #10295121) was performed according to the manufacturer's instructions of the Human Fab Capture Kit (Cytiva, #28958325) at a level of ~205 RUs. Recombinant huIL-6[5] was injected and flowed over the chip surface for 2 min at a flow rate of 30 µl min^{-1}. After each binding the sensor chip surface was washed and regenerated with 10 mM glycine-HCL (pH 2.1) for 2x 1 min at a flow rate of 30 µl min^{-1}. The data were analysed with Biacore X100 Control Software Version 2.1.0.201 (Cytiva). The graphs were plotted using GraphPad Prism 9 (version 11.0.2, Dotmatics). The determined K_D values are presented in Supplementary Table 3.

Supplementary Table 3: Summary of the affinities of C20 mAb and Fab to sIL-6R and Hy-IL-6

Target	C20	K_{on} [1/Ms]	K_{off} [1/s]	K_D [M]
sIL-6R	mAb	1.98 x10^{6}	5.08 x10^{-5}	2.6 x10^{-11}
	Fab	9.75 x10^{5}	2.98 x10^{-5}	3.06 x10^{-11}
Hy-IL-6	mAb	5.84 x10^{5}	3.78 x10^{-5}	6.48 x10^{-11}
	Fab	3.84 x 10^{5}	1.44 x10^{-7}	3.73 x10^{-13}

Supplementary Figure 17

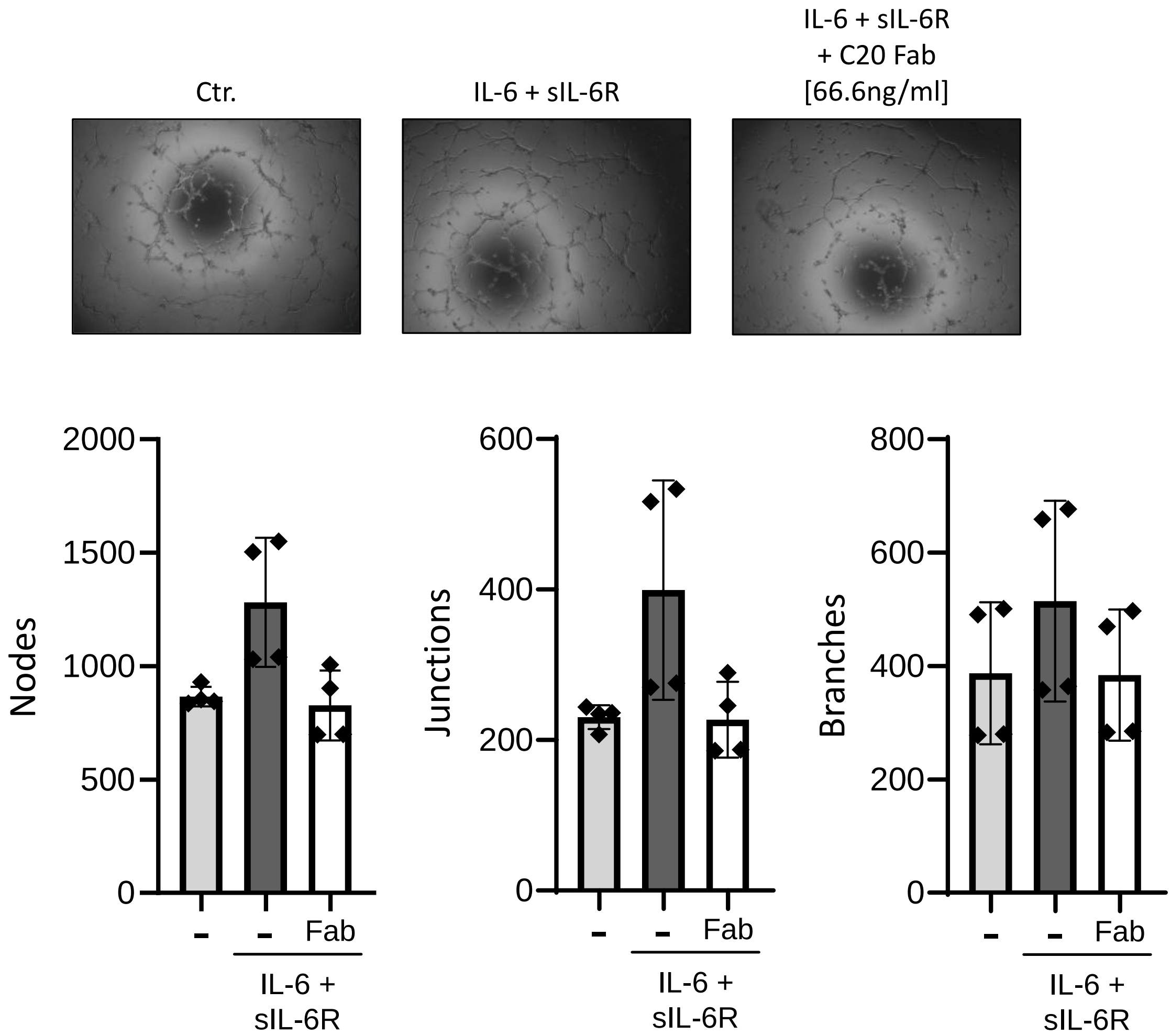

Supplementary Figure 17: C20 Fab reduces IL-6 trans-signalling-induced angiogenesis

HuARLT-2 cells were seeded for 48h in endothelial growth medium with supplements and 2 µg ml^{-1} doxycycline and then stimulated for 24 h with huIL-6 (50 ng ml^{-1}) and husIL-6R (500 ng ml^{-1}) as indicated in the figures. µ-Slide 15 well angiogenesis slides (Ibidi, #81506) were coated with Matrigel (Corning, #354230). Matrigel was thawed slowly on ice before using, dispensed into wells (10 µl per well), and allowed to polymerize for 30 min at 37 °C. After stimulation, cells were detached with Trypsin, counted and seeded at a density of 7,800 cells per well in 50 µl of their respective conditioned supernatants onto µ-slide 15 well pre-coated with Matrigel. Cells were incubated at 37 °C for 6 h in a water saturated atmosphere containing 5 % CO_2, after which three representative images were taken per well using an AMG EVOS microscope (Thermo Fisher Scientific). Representative raw data from n = 4 independent experiments are shown. Tube formation as represented by number of segments (Fig. 10D), nodes, junctions, and branches were analysed using Image J (version 1.54).[7]

Supplementary Note 4: Generation of anti-mesothelin mAb 1H7

Subtractive immunization was used to generate antibodies that only recognize membrane-bound mesothelin.[8] In brief, mice were immunized with recombinant full-length mesothelin, and then treated with cyclophosphamide. These mice were subsequently immunized with mesothelin-transfected P815 cells (P815-Meso). After 4 cycles of immunizations, mice were killed, total RNA was extracted from splenocytes, and Fab libraries were created by using phage display technology. Phage libraries were selected, against mesothelin-expressing Chinese hamster ovary (CHO) cells (CHO-Meso). A second round of panning was performed against P815-Meso cells. Non-transfected CHO and P815 cells were used as a control for panning to assess specific phage enrichment. After 2 rounds of panning for each library, Fab-containing periplasmic extracts of single colony cultures were collected and screened on CHO-Meso cells. All Fabs binding soluble mesothelin were eliminated. The anti-mesothelin Fab 1H7 was identified to selectively bind membrane-associated mesothelin. 1H7 was chimerized at RD Biotech with a human IgG1-expressing mammalian vector and transfected to CHO cells.

References

1 Crooks, G. E., Hon, G., Chandonia, J. M. & Brenner, S. E. WebLogo: a sequence logo generator. *Genome Res* **14**, 1188–1190 (2004). https://doi.org/10.1101/gr.849004

2 Fischer, M. *et al.* I. A bioactive designer cytokine for human hematopoietic progenitor cell expansion. *Nat Biotechnol* **15**, 142–145 (1997). https://doi.org/10.1038/nbt0297-142

3 Altschul, S. F. *et al.* Gapped BLAST and PSI-BLAST: a new generation of protein database search programs. *Nucleic Acids Res* **25**, 3389–3402 (1997). https://doi.org/10.1093/nar/25.17.3389

4 Garbers, C. *et al.* Species specificity of ADAM10 and ADAM17 proteins in interleukin-6 (IL-6) trans-signaling and novel role of ADAM10 in inducible IL-6 receptor shedding. *J Biol Chem* **286**, 14804–14811 (2011). https://doi.org/10.1074/jbc.M111.229393

5 Weitz, H. T. *et al.* Interleukin-11 receptor is an alternative alpha-receptor for interleukin-6 and the chimeric cytokine IC7. *FEBS J* **292**, 523–536 (2025). https://doi.org/10.1111/febs.17309

6 Zhou, Y. *et al.* Structural insights into the assembly of gp130 family cytokine signaling

7 Carpentier, G. *et al.* Angiogenesis Analyzer for ImageJ - A comparative morphometric analysis of "Endothelial Tube Formation Assay" and "Fibrin Bead Assay". *Sci Rep* **10**, 11568 (2020). https://doi.org/10.1038/s41598-020-67289-8

8 Asgarov, K., Balland, J., Tirole, C., Bouard, A., Mougey, V., Ramos, D., Barroso, A., Zangiacomi, V., Jary, M., Kim, S., Gonzalez-Pajuelo, M., Royer, B., de Haard, H., Clark, A., Wijdenes, J., and Borg, C. A new anti-mesothelin antibody targets selectively the membrane-associated form, MAbs **9**, 567–577 (2017). https://doi.org/10.1080/19420862.2017.1288770
