# Supplementary material for: Selective inhibition of IL-6 trans-signalling reveals distinct mechanisms of human classic and trans-signalling receptor-complex formation
Source: Commun Biol. 2026 Oct 14;9:1340. doi: 10.1038/s42003-026-11014-0 (PMC13669321; doi:10.1038/s42003-026-11014-0)
Supplement: Supplementary file 3 [file 42003_2026_11014_MOESM3_ESM.pdf]

Description of Additional Supplementary Files

File name: Supplementary Data 1

Description: . Source data underlying graphs.
